# Organometallic Triazoles with Phototunable Properties as Cytotoxic Agents for Potential Anticancer Applications

**DOI:** 10.3390/molecules31213932

**Published:** 2026-11-01

**Authors:** Lyudmila N. Telegina, Elena S. Kelbysheva, Aleksandr A. Chamkin, Anastasia S. Zemskaya, Pavel N. Solyev, Maxim S. Dashko, Anastasia A. Danshina, Alexey N. Rodionov

**Affiliations:** 1 A.N. Nesmeyanov Institute of Organoelement Compounds, Russian Academy of Sciences, Vavilova St. 28, Bld. 1, Moscow 119334, Russia; chalex@ineos.ac.ru (A.A.C.); dashko-03@mail.ru (M.S.D.); danshina.aa@phystech.edu (A.A.D.); 2 Engelhardt Institute of Molecular Biology, Russian Academy of Sciences, Vavilova St. 32, Moscow 119991, Russia; a.zemskaia@mail.ru (A.S.Z.); solyev@gmail.com (P.N.S.)

**Keywords:** 1,2,3-triazole, cymantrene, ferrocene, cytotoxic agent, photochemistry, photophysical properties

## Abstract

The creation of organometallic–organic hybrid molecules opens the way to the creation of biologically active molecules with a broad spectrum of activity. To simplify the creation of such compounds, it is necessary to establish structure–activity relationships and physicochemical property–activity relationships. 1,2,3-Triazoles containing a cymantrenylalkyl or ferrocenylalkyl moiety were investigated in this work. Studies of the photophysical properties revealed that fluorescence was most influenced by the viscosity of the medium and the formation of aggregates. The redox properties of 1,2,3-triazoles were determined by the organometallic moiety and its position in the heterocyclic ring. Experimental and theoretical studies showed that the organometallic moiety played a key role in varying the electronic properties and reactivity of 1,2,3-triazoles. Only cymantrene derivatives exhibited photochemical activity, and photolysis resulted in the formation of stable chelate complexes with a Mn–N2 bond in the heterocycle. It is worth noting that the chelates were the most reactive and underwent oxidation most readily. Studies on the antitumor activity of 1,2,3-triazoles showed that 1-cymantrenylethyl-4-phenyl-1,2,3-triazole (**4**) exhibited the highest cytotoxicity in the dark (A549 (CC50 = 9.0 ± 0.6 µM) and HCT116 (CC50 = 15.9 ± 2.2 µM)), whereas the effect of irradiation was inconsistent and depended on the incubation time. Thus, this study opens the way for the rational design of biologically active molecules based on phototunable organometallic compounds.

## 1. Introduction

Today, one of the main pharmacophoric groups among nitrogen-containing heterocycles is the 1,2,3-triazole ring, a five-membered ring containing three nitrogen atoms [1]. Triazoles can also act as linkers for binding to other pharmacophoric groups [2,3]. The important role of this group as a bioactive fragment is primarily due to the ease of its synthesis and modification. Thus, one of the main methods for synthesizing triazoles is the copper-catalyzed azide–alkyne cycloaddition (click reaction). In addition, 1,2,3-triazole can form various non-covalent interactions such as hydrogen bonds, van der Waals, and dipole–dipole interactions with enzymes, proteins, and receptors [4,5,6]. This property is one of the reasons why 1,2,3-triazole derivatives exhibit many promising biological properties, including antibacterial [7,8,9], antimalarial [10,11], antituberculosis [12,13], antiviral [14,15], antiparasitic [16], and antitumor [17,18] properties. Importantly, 1,2,3-triazoles can also exert anticancer effects [19,20,21,22], causing cell-cycle arrest and apoptosis in cancer cells. Triazoles are polydentate ligands that form stable complexes with metals, which also exhibit various biological activities [23], including high anticancer activity. One of the triazole-amine platinum complexes exhibits selective cytotoxicity against breast cancer cell lines [24]. The introduction of organometallic fragments into the triazole molecule also makes it possible to obtain target products with desired properties. Several mono- and heterobimetallic complexes with triazole moieties showed significant antitumor activity against colon cancer cells (CT26 and HT29), with ferrocene and dipyridylamine complexes demonstrating the best inhibitory activity, comparable to that of cisplatin [25]. Thus, targeted synthesis of new promising organometallic drugs containing a triazole fragment not only solves the problem of resistance to existing drugs but also opens the way to new promising medicinal products.

Ferrocene is a well-studied scaffold for obtaining promising molecular materials. Its derivatives are known to exhibit high biological activity, and the mechanisms of action of a number of these derivatives were well studied [26,27,28]. Ferrocene derivatives have proven to be effective, including as anticancer agents [29,30], with efficacy comparable to that of the well-known anticancer drug cisplatin [31,32]. A much less studied, but no less promising, group comprises cymantrene derivatives (cyclopentadienyl manganese tricarbonyl). Previously, it was shown [33] that cymantrene derivatives with various linkers attached to the sC18 peptide exhibited high cytotoxic activity against human breast adenocarcinoma cells (MCF-7) and human colon carcinoma cells (HT-29). Also, the introduction of the cymantrenyl fragment into various biologically active molecules increases the cellular permeability of the drug [33]. Manganese was found to accumulate in cancer cells, which meant that compounds containing the cymantrenyl group were transported across the cell membrane. Once inside a cell, such compounds can undergo photolysis upon exposure to light, resulting in the cleavage of a carbonyl group (releasing CO); this leads to the death of cancer cells or a reduction in their viability (depending on the concentration of the substance administered). Thus, this class of compounds is characterized by high phototoxicity. Such light-sensitive organometallic compounds are capable of generating ROS under low oxygen tension or functioning through alternative mechanisms that complement the ^1^O^2^ pathway. This ability allows for the effective treatment of hypoxic tumors [34]. Manganese carbonyl complexes can be classified within a broader group of potent anticancer agents known as PhotoCORMs, which exhibit high cytotoxicity against virtually all cancer cell lines [35,36,37,38]. Their potential is further underscored by the unique action of CO, which has also been shown to play a role in biological processes.

**Figure 1:**
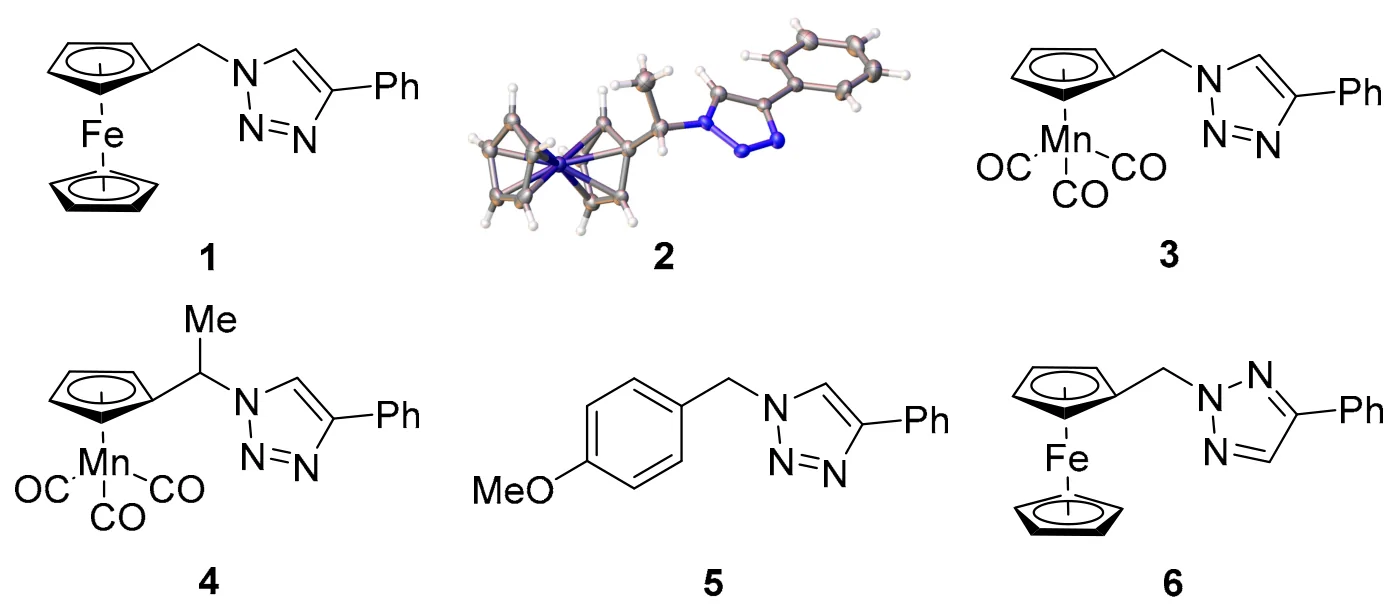
Structures of 1-ferrocenylalkyl-4-phenyl-1,2,3-triazoles (**1**, **2**), 1-cymatrenylalkyl-4-phenyl-1,2,3-triazoles (**3**, **4**), 2-ferrocenylmethyl-4-phenyl-1,2,3-triazole (**6**), and 1-*p*-methoxybenzyl-4-phenyl-1,2,3-triazole (**5**).

The search for new active agents for the development of anticancer drugs—including those intended for the treatment of currently incurable forms of cancer—is one of the most critical tasks in modern pharmaceutical chemistry [39,40,41,42]. The aim of this work was to develop an interdisciplinary approach for the creation of phototunable cytotoxic agents with variable and controllable photophysical and biological properties, which can be used, among other things, in cancer theranostics. Analysis and comparison of the results obtained by UV–visible and fluorescence spectroscopy, cyclic voltammetry, and non-covalent interaction calculations, as well as testing of cytotoxic biological activity in three cell lines—A549 (human non-small cell lung carcinoma), HCT116 (human colorectal cancer), and MCF7 (breast cancer)—under dark conditions and UV irradiation, open the way for the rational design of bioactive compounds and the prediction of their properties. To establish the influence of various metals in coordination compounds on the antitumor activity of triazoles in comparison with that of the organic analog, as well as the relationship between the structure of the complex and its activity, ferrocene- and cymantrene-containing derivatives of 1,2,3-triazoles, along with an organic analog, were synthesized, as shown in Figure 1. It is worth noting that the anticancer activity of the organic triazole **5** was studied previously [43], allowing compound **5** to be used as a negative benchmark for evaluating the efficacy of organometallic triazoles.

## 2. Results

### 2.1. Synthesis of 4-Phenyl-1,2,3-triazoles and X-Ray Crystallographic Study

The synthesis of triazoles **1**–**5** was carried out using our previously described copper-catalyzed azide–alkyne cycloaddition click reaction methodology [44]. The compound **2** was synthesized using our previously developed method, which enabled an increase in product yield. The synthesis of derivative **6** proceeded via migration of the organometallic substituent during alkylation of the triazole ring, a process that we have previously studied in detail [45]. The structure of all the obtained compounds was confirmed by spectroscopic methods, and, in the case of triazole **2**, X-Ray structural analysis was carried out on single crystals.

Single-crystal X-Ray diffraction (XRD) shows that the crystal packings of all 4 metal–organic triazole-containing complexes, including 1 newly synthesized complex, 3 reported previously **1** (**UCEDUE**) and **6** (**UCEDOY**) from [45], and **3** (**LAZDAU**) from [46], differ significantly; however, they are formed by similar interactions between similar groups.

Analysis of the crystal packing revealed that compounds **1** and **2** form dimers stabilized primarily by hydrogen bonds between the ferrocenecyclopentadienyl ring and the triazole ring (Figure S22). In the case of compound **2**, the dimers form chains that ultimately result in a Christmas-tree packing arrangement. The hydrogen bonds involved in the formation of the crystal packing could be classified into three types: weak hydrogen bond between the cyclopentadienyl (Cp) ring of the ferrocene and triazole ring C(2)–H(2)···N(2) and C(4)–H(4)···N(3) (the distances of C···N and N···H were 3.611(9), 3.451(10) and 2.701(6), 2.727(6) Å, respectively), hydrogen bond between two triazole rings C(12)–H(12)···N(3) (the distances of C···N and N···H were 3.621(10) and 2.698(6) Å, respectively) and hydrogen bond between triazole ring and methyl group C(20)–H(20C)···N(2) (the distances of C···N and N···H were 3.652(10) and 2.673(6) Å, respectively). In contrast to the 1,4-disubstituted triazoles **1**–**2**, compound **6** formed stacks along the b-axis due to strong stacking interactions. The packing of the molecular stacks was ladder-like, with a dihedral angle of 0° between the planes of the phenyl and triazole rings.

In the case of replacement of ferrocene with cymantrene (from **1** to **3**), the crystal packing changed and turned into a 3D hydrogen-bonded framework (Figure S22). It was formed by C–H···O–C interactions between CO groups and CH_2_, – C(9)–H(9A)···O(1) and C(9)–H(9A)···O(3) (the distances of C···O and O···H were 3.9529(15), 3.6911(15) and 3.0931(10), 2.8825(9)Å, respectively) or phenyl ring C(15)–H(15A)···O(2) (the distances of C···O and O···H were 3.652(10) and 2.673(6) Å, respectively), Cp-ring of cymantrene and triazole-ring C(4)–H(4A)···N(2), C(5)–H(5A)···N(2), C(5)–H(5A)···N(1) and C(4)–H(4A)···N(3) (the distances of C···N and N···H were 3.5235(18), 3.3805(17), 3.8231(16), 3.7947(18) and 2.9124(12), 2.4542(12), 2.9306(10), 2.9189(12) Å, respectively), triazole–triazole rings C(10)–H(10A)···N(1) (the distances of C···N and N···H were 3.5740(16) and 2.6915(12) Å, respectively), triazole–phenyl rings C(17)–H(17A)···N(3) (the distances of C···N and N···H were 3.8032(19) and 3.0467(11) Å, respectively) and triazole–CH_2_-group C(9)–H(9B)···N(2) (the distances of C···N and N···H were 3.9371(16) and 3.1601(12) Å, respectively).

To quantify the resulting intermolecular interactions in the obtained crystals, the Hirshfeld surfaces were analyzed [47], partitioning the space into “molecular” domains, mapping intermolecular interactions by the distances to the closest atom inside (di) and outside (de) of these domains, and quantifying their occurrence by the 2D fingerprint plots [48].

**Figure 2:**
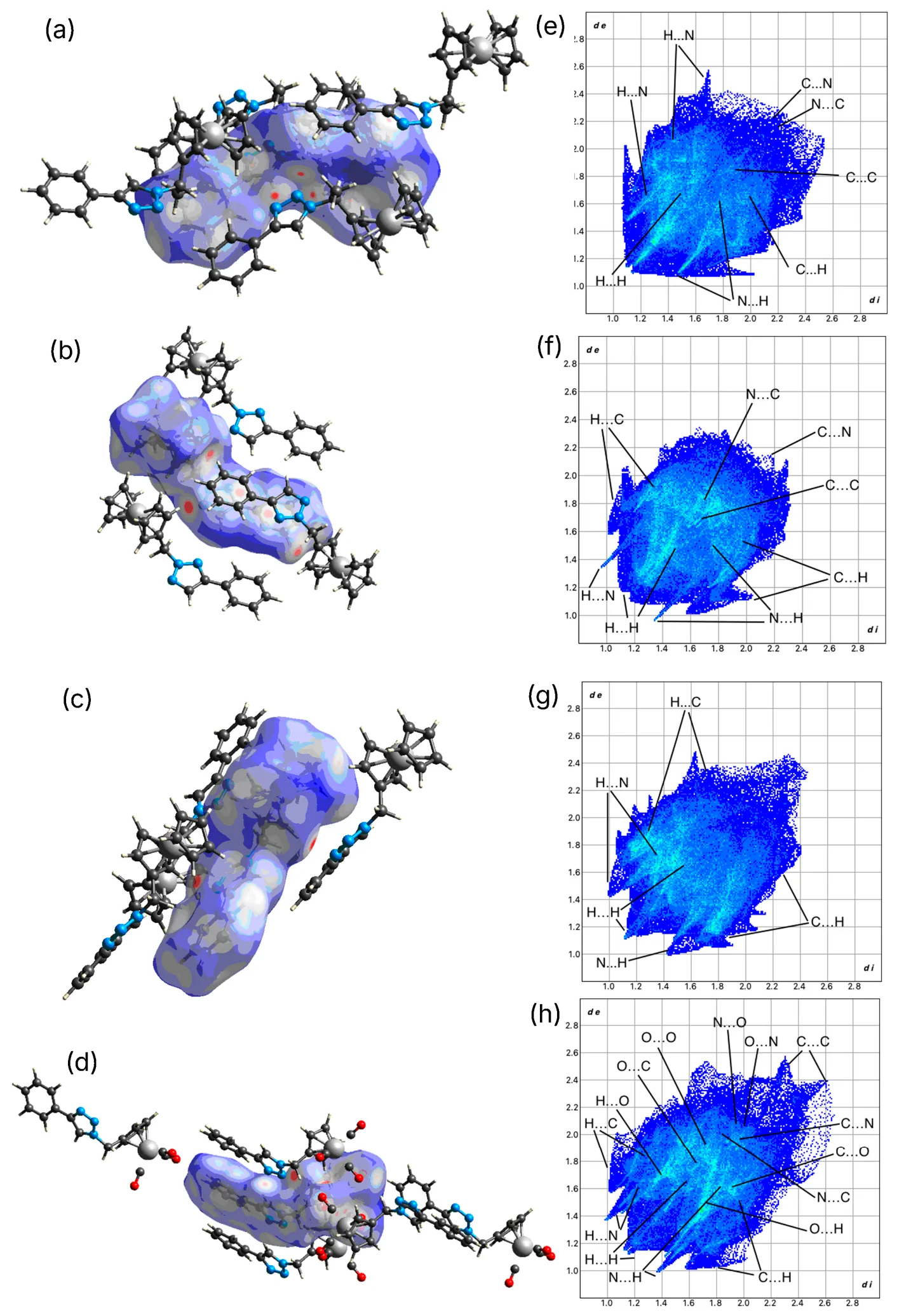
Hirshfeld surfaces of organometallic triazoles: (**a**) **2**, (**b**) **6** [45], (**c**) **1** [45], and (**d**) **3** [46]; the corresponding 2D “fingerprint” plots (**e**–**h**) were generated using Crystal Explorer software (version 17) [49]. For surfaces (**a**–**c**), intermolecular contacts are shown in red, white, and blue, representing cases where the interatomic distances are, respectively, less than, equal to, or greater than the sum of the corresponding van der Waals radii. In the scatter plots (**e**–**h**), regions with high and low densities of points representing pairs (di, de) are highlighted in green and blue, respectively.

The bright red spots on the Hirshfeld surfaces of compounds **1** [45], **2**, **3** [46], and **6** [45] resulted from N···H–C interactions between the triazole ring and the CH group of the triazole ring, CH_3_ group, Cp ring, or phenyl group of neighboring molecules of the corresponding complex (Figure 2a–d). They appeared on the 2D fingerprint plots as sharp spikes (Figure 2e–h), accounting for up to 8.5% (for **1**), 7.7% (for **2**), 7.7% (for **3**), and 5.5% (for **6**) of the corresponding Hirshfeld surfaces. For the complex **3**, the CO-groups were additionally involved in O…C interactions with the CO-groups of the cymantrene moiety of the other molecule (4.2% and 5.0% for the reciprocal C…O contacts), while weaker red features corresponding to Cp…C interactions between the Cp-ring and the CO-groups were obscured by the more abundant C-H…C contacts.

All other types of intermolecular interactions were summarized in Table S1. The most numerous were H…H contacts, which accounted for the majority of the Hirshfeld surfaces in all structures (Table S1); their contribution varied depending on the molecular packing and the presence of specific donor/acceptor groups capable of forming stronger directional interactions.

### 2.2. Photophysical Properties of Triazoles ***1***–***6***

**Figure 3:**
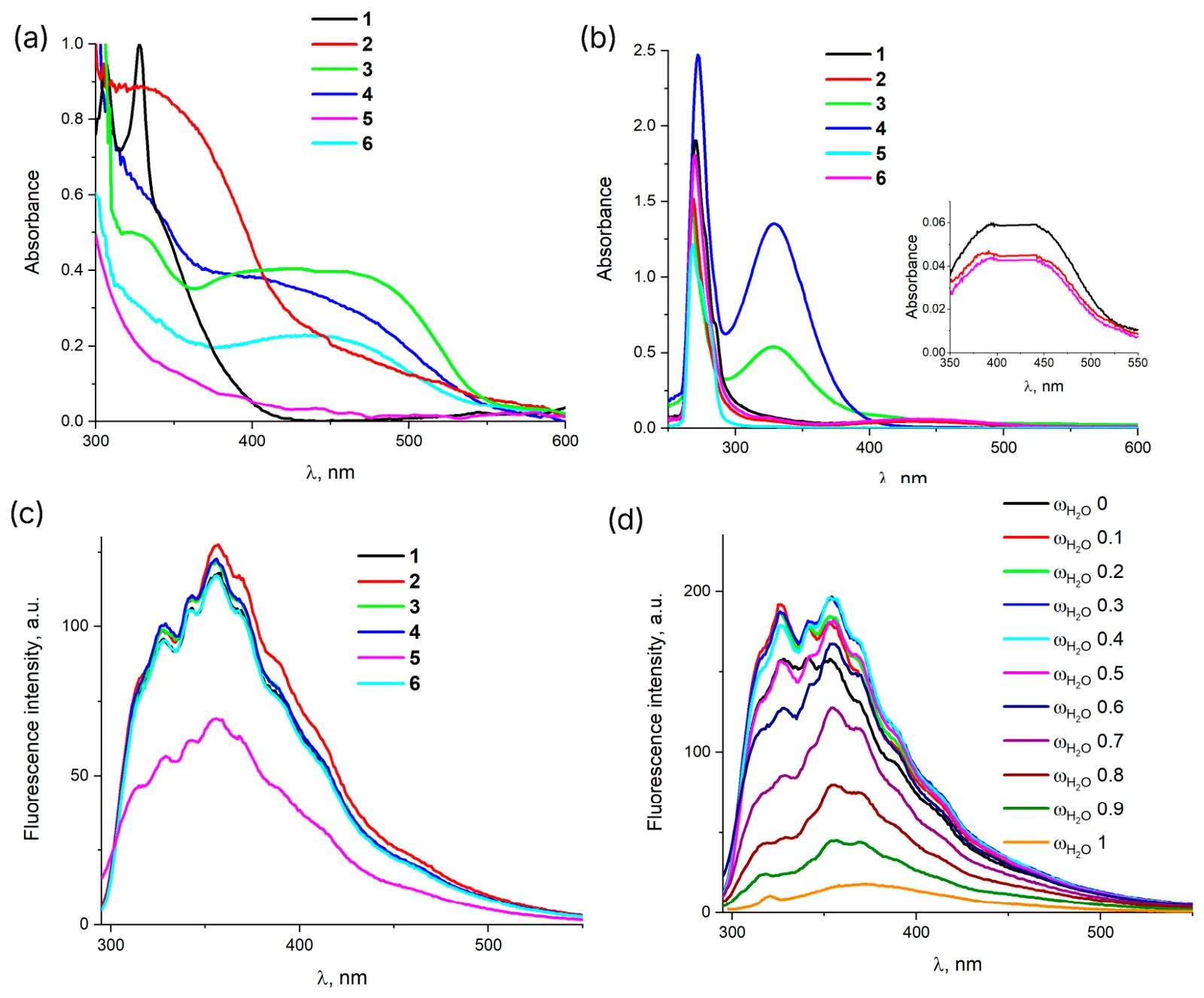
(**a**) UV–visible spectra of **1**–**6** recorded in the solid phase. (**b**) UV–visible spectra of **1**–**6** (C = 5 × 10^−4^ M) in ethanol. (**c**) Fluorescence spectra of **1–6** (C = 2 × 10^−5^ M) in ethanol. (**d**) Fluorescence spectra of **4** (C = 2 × 10^−5^ M) recorded in aqueous ethanol solutions with different water contents.

To determine and compare the reactivity of 1,2,3-triazoles, compounds **1**–**6** were studied using solid-phase UV–visible spectroscopy. The UV spectra of **3** and **4** were shown to be similar, as the observed bands are due to d–d* transitions in the organometallic moiety (Figure 3a, Table 1) [50,51]. A similar type of spectrum was observed for derivatives **1**–**2** and **6**, whose absorption bands in the region of 350–500 nm are associated with d–d* transitions in the ferrocenyl group [52,53]. However, a number of peculiarities can be identified that were observed when varying the substituents at the α-position of the Cp ring and changing the position of the organometallic fragment in the triazole ring. For example, for triazole **3**, unlike **4**, splitting of the absorption band in the 300–350 nm region was observed, which may be due to a change in the vibrational structure of the molecules resulting from the replacement of the hydrogen atom in **3** with the bulky, electron-donating methyl group in **4**. Differences in the absorption band characteristics were also observed for the ferrocenyl derivatives. The half-height width of the absorption band at 450 nm decreases in the order **1** < **6** < **2**, which was likely also due to changes in the vibrational structure of the molecules during their structural modification. For the organic analog **5**, the absorption maximum was not determined, since in the 300–600 nm region only the edge of the absorption band was observed due to the π–π* transition in the aromatic system involving the triazole ring. Determination of the optical energy gap (E_g_^opt^) showed that it decreased in the series **5** > **3** > **4** > **1** > **6** ≈ **2** (Table 1). Thus, the introduction of a methyl group at position *1* of the side substituent instead of a hydrogen atom had a more significant effect on the photophysical and electronic properties of cymantrenyl derivatives. The greatest decrease in the difference between the highest occupied molecular orbital (HOMO) and the lowest unoccupied molecular orbital (LUMO) was observed when the substituent at one of the nitrogen atoms of the triazole ring was changed from an organic group to a cymantrenyl group and then to a ferrocenylalkyl group. Consequently, reactivity increased in the order **5** > **3** > **4** > **1** > **6** ≈ **2**.

When studying the absorption spectra in the range of 250–800 nm in ethanol (Figure 3b, Table 1), it was shown that compounds **1**–**4** and **6** have absorption bands characteristic of organometallic fragments (ferrocenyl or cymantrenyl) and caused by d–d* transitions, in the region of 330 nm for **3**–**4** and 420 nm for **1**–**2** and **6**. It is worth noting that the extinction values for the derivatives with ferrocenyl groups turned out to be close both to one another and to that of ferrocene itself, although an increase in extinction is usually observed upon the introduction of substituents [54]. The extinction ratio for **4** and **3** was 1.5, and it increased when moving from ethanol to benzene [46]. For all organometallic compounds, another band was observed at 270 nm, the extinction of which was, in all cases, around 3000. This band is attributed to π–π* transitions within the aromatic systems of molecules **1**–**4** and **6**, involving both the organometallic moiety (the Cp ring) and the heterocyclic components (the triazole and phenyl rings) [55]. For the organic analog **5**, three bands were observed at 268, 274, and 281 nm, which were apparently associated with π–π* transitions in the aromatic system of the triazole and phenyl rings; the extinction of the band at 268 nm was equal to 1900.

Fluorescence studies of **1**–**6** in ethanol revealed similar spectra upon excitation with light at λ_Ex_ = 285 nm (Figure 3c, Table 1). The fluorescence maximum was observed at 355 nm, and the quantum yields (Φ) for **1**–**4** and **6** were similar, varying in the range of 0.3–0.4, while Φ for **5** decreased to 0.24, and the Stokes shifts ranged from 73 to 90 nm for all triazoles (Table 1). The absorption and emission spectra were clearly not mirror images of each other, which could be due to several factors. First, since the emission spectra were independent of the nature of the substituent on one of the nitrogen atoms, the emission occurred upon excitation of a conjugated system containing a triazole ring and a phenyl substituent at the *4*-position. We assume that excited-state energy transfer between the organometallic moieties in **1**–**4** and **6** and the triazole rings likely occurred nonradiatively because the absorption and emission bands of the organometallic groups at 270 nm overlapped with those of the triazole systems, allowing efficient energy transfer (Figure 3b). This energy transfer may explain the approximately 1.5-fold increase in the fluorescence quantum yields of triazoles **1**–**4** and **6** (sensitized fluorescence) compared with that of the organic analog **5**. The lack of mirror symmetry, especially in the case of **5**, can also be attributed to the fact that the emitting triazole system apparently contains several nearby excited states with different oscillator strengths. This was confirmed by a rough convolution of the absorption bands, with the energy splitting for all triazoles being approximately 0.09–0.15 eV.

The intrinsic fluorescence of the triazole system arose from planar charge transfer between the phenyl substituent at position *4* and the heterocycle. The substituent at N1 or N2 was not involved in conjugation and was out of the plane of the triazole [45,46] and, therefore, did not participate in charge transfer or affect the fluorescence of **1**–**6**, in contrast to examples previously reported in the literature [56]. Thus, the dihedral angle between the triazole ring and the phenyl substituent in **3** was 14.46°. Consequently, altering the structure of triazole derivatives will alter the optical properties of the molecules, opening the way for monitoring possible transformations and the mechanism of action of triazoles in biological environments, including possible cleavage of the metal–cyclopentadienyl ring bond and the organometallic fragment–triazole ring bond.

The effect of aggregation on the fluorescence properties was studied using sample **4** in aqueous ethanol solutions with varying water content (from 0 to 1) (Figure 3d), as 1,2,3-triazoles are highly soluble in alcohol but poorly soluble in water. Initially, an increase in fluorescence intensity was observed as the water content in the solution increased to 0.3. Further increases in water content in the solution led to fluorescence quenching. The intensity of the emission bands declined non-symmetrically, with the intensity of the bands at 326 nm decreasing faster than that of the bands at 355 nm. Furthermore, changing the water content from 0.3 to 1.0 resulted in a shift in the emission maximum from 355 to 375 nm. Thus, based on the data obtained, we put forward a hypothesis that the initial increase in emission was apparently related to the influence of solution viscosity, as a low water content in ethanol leads to the formation of hydrates and an increase in the strength of hydrogen bonds, leading to an increase in the viscosity of water–ethanol mixtures. The maximum viscosity of the solutions is observed at a water content of approximately 0.3. Further increases in water content led to a decrease in viscosity, which in turn led to a decrease in emission intensity. Another factor in the shift in the emission maximum and fluorescence quenching in solutions with a high content of water was the increased aggregation of triazole **4**. The maximum fluorescence is also observed at 0.3, which indirectly confirms our assumption about the influence of solution viscosity on the emission intensity. Therefore, probably up to ω(H_2_O) = 0.3 in an aqueous–ethanol solution, the viscosity of the solution and aggregation were competing factors influencing the intensity of the fluorescence bands; viscosity could be the more significant factor. With increasing water content, a synergistic effect of changes in solvent viscosity and aggregation of **4** was likely observed. The change in fluorescence due to aggregation opens the way to monitoring intermolecular interactions of triazoles and predicting their aggregation behavior in various systems.

### 2.3. Electrochemical Behavior of Triazoles ***1***–***6***

**Figure 4:**
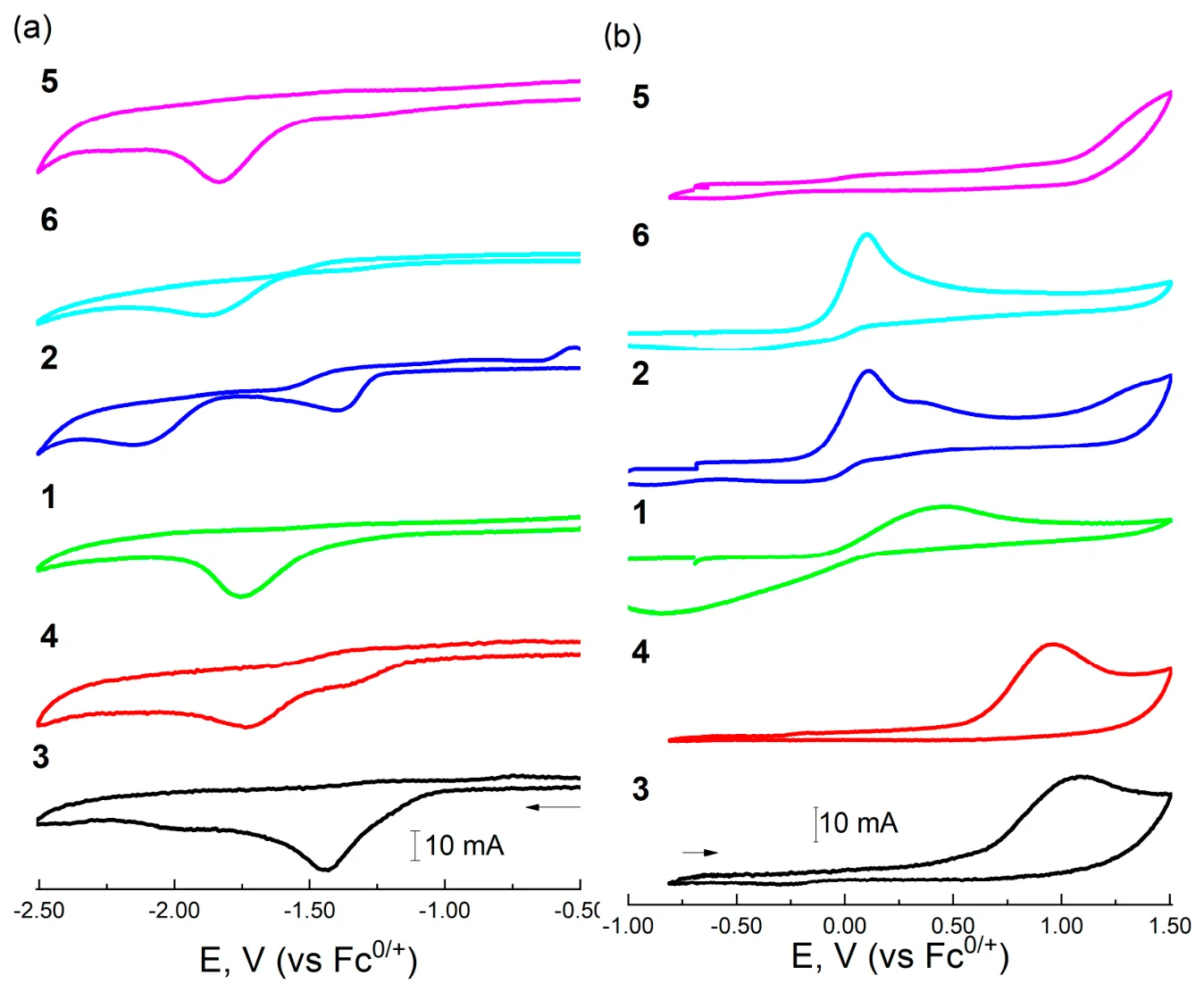
(**a**) Cyclic voltammograms of the reduction branch of **1**–**6** in a 0.1 M tetraethylammonium tetrafluoroborate solution in ethanol. Scan rate: 100 mV/s. (**b**) Cyclic voltammograms of the oxidation branch of **1**–**6** in a 0.1 M tetraethylammonium tetrafluoroborate solution in ethanol. Scan rate: 100 mV/s.

The electrochemical behavior of organometallic compounds **1**–**4** and **6**, containing a substituted 1,2,3-triazole fragment in the side chain of the Cp ring, was studied by cyclic voltammetry and compared with the electrochemical parameters of the organic analog **5**. In the reduction branch, in the case of compounds **1**–**3** and **5**, only one irreversible reduction wave with the transfer of two electrons is observed, as established by comparison of the current with that of the Fc^0/+^ standard and by the Randles–Sevcik equation for reversible and irreversible processes (Figure 4a, Table 2):

$$\left|I_{k}\right|=0.446{\left(\frac{F}{R\cdot T}\right)}^{\frac{1}{2}}{\alpha^{\frac{1}{2}}n}^{\frac{3}{2}}\mathrm{FS}D_{0}^{\frac{1}{2}}C_{0}v^{\frac{1}{2}}\;\mathrm{or}\;\left|I_{k}\right|=A\alpha^{\frac{1}{2}}n^{\frac{3}{2}}SD_{0}^{\frac{1}{2}}C_{0}v^{\frac{1}{2}}$$

where I_k_ is the peak cathodic current, A; F is the Faraday constant, 96,485 C·mol-eq^−1^; R is the universal gas constant, 8.314 J·mol^−1^·K^−1^; T is the temperature, 298 K; n is the number of electrons transferred; S is the electrode surface area, cm^2^; D is the diffusion coefficient, cm^2^·s^−1^; C_0_ is the molar concentration of the compound under study, mol·cm^−3^; C_0_ is between 1.0 × 10^−3^ mol·cm^−3^ and 1.0 × 10^−3^ mol·cm^−3^, v is the potential scan rate, V·s^−1^, A is 2.69 × 10^5^ for reversible waves or 2.99 × 10^5^ for irreversible waves, α is charge transfer coefficient, α is 1 for reversible waves or 0.5 for irreversible waves. The diffusion coefficient for organometallic compounds was taken to be 4.7 × 10^−6^ cm^2^·s^−1^ based on literature data [57]. The lower potential at which electron transfer occurred for **1** than for organic analog **5** was likely due to intramolecular interactions between the nitrogen atoms of the triazole ring and iron, which led to a redistribution of electron density within the molecule. When moving from 1,4-substituted triazole **1** to 2,4-substituted triazole **6**, a significant increase in the reduction potential was observed, which was associated with changes in the intramolecular interactions between the heterocycle and iron and consequently led to a redistribution of electron density. When the hydrogen atom at position *1* of the Cp ring side chain was replaced by a methyl group, two reduction waves were observed, with the sequential transfer of one electron in each step. This change was apparently due to the facilitation of the addition of the second electron by the introduction of additional steric bulk, rather than to a change in the donor–acceptor properties of the substituent, as we previously demonstrated for phthalimide derivatives containing a cymantrenyl fragment [58]. In the case of derivatives **2** and **4**, the initial reduction potentials were close, which was most likely due to intramolecular interactions between the iron atom and the triazole ring in the ferrocene-containing derivative **2**. Such interactions can facilitate the reduction process by reducing the electron density in the heterocyclic ring. The transfer of the second electron was easier for the cymantrenyl analog than for the ferrocenyl derivative, and the difference in reduction potentials was 0.43 V. This showed that, after the first reduction, the interactions that facilitated the reduction of **4** were significantly weakened, and the donor–acceptor properties of the organometallic substituents began to play a major role.

Oxidation of the organometallic derivatives proceeded with the transfer of only one electron in all cases (Figure 4b, Table 2). The ferrocenyl fragment was oxidized at approximately 0.1–0.4 V, and the cymantrenyl group at approximately 1 V. However, only the oxidation processes of **1** and **2** were quasi-reversible; in the case of **2**, a second oxidation wave was observed, which was apparently associated with the possible coordination of the BF_4_^−^ anion. The introduction of a methyl moiety in place of the hydrogen atom resulted in a decrease in the potential, indicating that the methyl group acted as a sigma donor to the organometallic moiety. The 2,4-disubstituted triazole **6** was also more readily oxidized than **1**, possibly due to reduced interactions between the triazole ring and the iron atom, consistent with the ease with which triazoles **1** and **6** were reduced. Thus, based on the CV, compounds **2** and **6** were the most easily oxidized, making them candidates for further investigation of their antioxidant properties [59]. Furthermore, because compound **6**, unlike compound **2**, undergoes irreversible oxidation, it appears to be the most promising candidate.

Calculation of the electronic parameters showed that the ionization potential for **3** and **4** was the highest, while the IPs for **1** and **6** were similar and 0.13 eV lower than the IP for **2**. This indicated that, in addition to the donor–acceptor properties of the substituents, the IP value was also determined by intramolecular interactions. The introduction of a methyl group had a weak effect on the cymantrenyl derivatives, but for the ferrocenyl derivatives, its influence on the ionization potential was more noticeable. When examining the electron affinity, it was clear that in all cases, the introduction of a donor methyl group and a change in the position of the ferrocenyl group in the triazole ring led to a decrease in EA. Thus, the energy gap varied in the following order: **6** < **2** < **1** < **4** < **3** << **5**; this order correlates with that obtained from the optical E_g_ calculations. It can be noted that structural changes in triazole derivatives had the greatest impact on the E_g_ value in the case of ferrocenylalkyl substituents.

### 2.4. DFT Calculations of Triazoles ***1***–***6***

Optimized structural parameters for the triazole 3 under study were calculated using the TPSSh method and are presented in the Supporting Information (Table S3). Bond lengths were compared with experimental XRD data, which are also provided in the Supporting Information. The TPSSh method was shown to yield a small error (less than 0.01 Å); therefore, the geometries of compounds **1**–**2** and **4**–**6** were also calculated using the TPSSh method.

**Figure 5:**
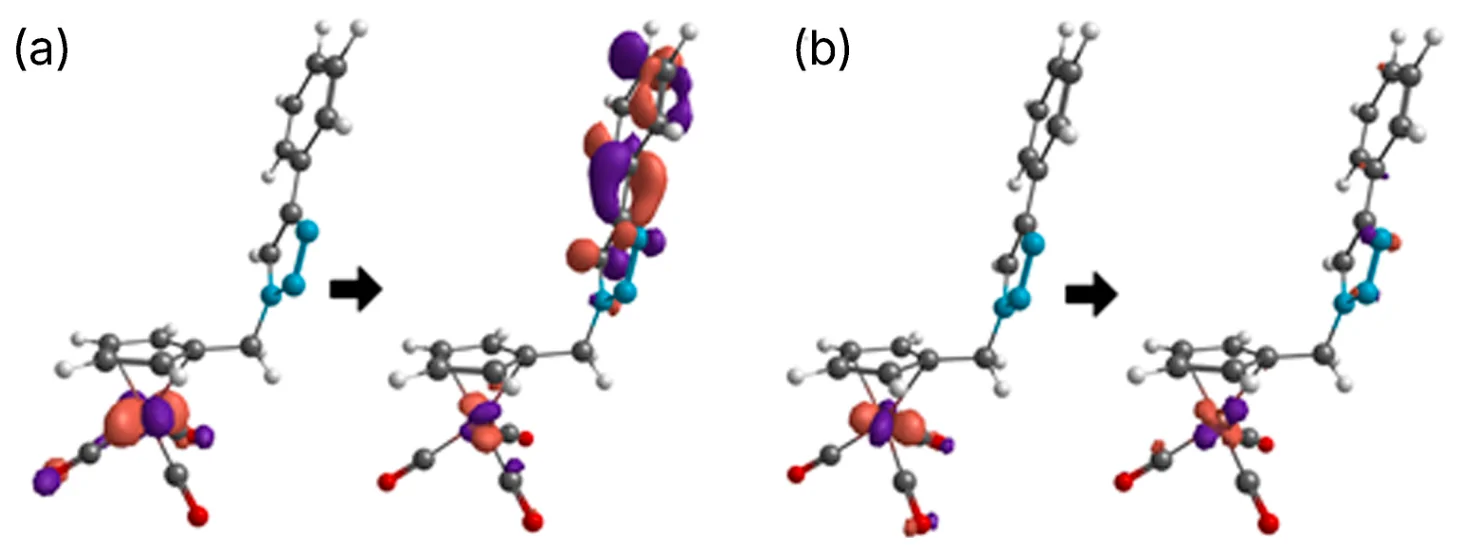
NTO analysis of **3**: (**a**) transition at 310.7 nm; (**b**) transition at 307.3 nm.

Calculations of the electronic absorption spectrum of the cymantrenyl derivative **3** (Table S9) revealed two absorption bands in the 200–400 nm range—at 255 and 310 nm—which are in qualitative agreement with the experimental absorption spectrum of **3**. It is worth noting that, according to TD-DFT calculations based on the TPSSh-D3BJ/def2-TZVPP geometry, the band at 310 nm consisted of two significant transitions at 310.7 and 307.3 nm (Table S9). Natural transition orbital (NTO) analysis revealed that the first transition (310.7 nm) consisted of 81.3% transfer of electron density from the manganese *d* orbital to the triazole ligand π* orbital (Figure 5a, Table S10); that is, the absorption arose from metal-to-ligand charge transfer (MLCT). The second transition (307.3 nm) comprised two degenerate transitions within the metal *d* shell, contributing 63.4% and 31.8% (95.2% in total; Figure 5b, Table S11). It is worth noting that MLCT is uncharacteristic of tricarbonyl cymantrene derivatives [51,60], which may be attributed to the fact that the triazole heterocycle can exhibit either donor or acceptor properties depending on the nature of its substituent.

Frontier molecular orbitals (FMOs), such as HOMO and LUMO, play a crucial role in determining the chemical stability of a molecule [61]. FMOs indicate a molecule’s ability to donate and accept electrons and provide insight into its reactivity, allowing the active site to be predicted based on the distribution of the frontier orbitals. The energy gap(E_g_) between HOMO and LUMO helps characterize the chemical reactivity, kinetic stability, and bioactivity of the molecule, which are influenced by intramolecular charge transfer. The calculated HOMO, LUMO, and E_g_ values are listed in Table 3 and Table S4; the order of the energy gaps—**6** < **2** < **1** < **4** < **3** << **5**—is consistent with the values experimentally determined via UV–Vis spectroscopy and CV.

Based on the HOMO and LUMO energy values, several general reactivity parameters were also determined, such as absolute electronegativity (χ = (E_HOMO_ + E_LUMO_)/2), absolute chemical hardness (η = (E_LUMO_ − E_HOMO_)/2), absolute chemical softness (σ = 1/η), chemical potential (μ = −(E_HOMO_ + E_LUMO_)/2), global softness (ζ = 1/2η), global electrophilicity (ω = μ^2^/2η), and the maximum additional charge transfer index (ΔN_max_ = −μ/η), which were listed in Table 3. As the data show, cymantrene derivatives exhibited the highest electronegativity and, consequently, the strongest acceptor properties. However, replacing a hydrogen atom with an electron-donating methyl group had a smaller effect on reducing electron attraction than migrating the organometallic moiety from the *1*-position to the *2*-position of the heterocyclic ring. Based on global softness ζ, it was shown that compound **6** exhibited the highest reactivity and polarizability, whereas the organic analog **5** displayed the lowest reactivity. Global electrophilicity indicated that cymantrenyl derivatives were more prone to react with soft nucleophiles, and the introduction of a methyl group should facilitate the reaction. Positive values of the maximum additional charge transfer index indicated that all the triazoles acted as acceptors, with the highest electron-accepting ability observed for compounds **3**, **4**, and **6**. Thus, it can be hypothesized that ferrocenyltriazole **6** might exhibit the highest biological activity.

Molecular electrostatic potential (MEP) analysis is a reliable method for investigating the chemical reactivity of molecular systems, hydrogen-bonding potential, and electrophilic and nucleophilic regions. Molecular electrostatic potential serves as an indispensable visual tool for analyzing the active binding site of a molecule through color-coding [61]. The method also enables a broader understanding of the relationship between the structure and activity of bioactive molecules. Different colors on the MEP map correspond to varying contributions to the electrostatic potential values on the molecular surface. Red indicates regions with the most electronegative (attractive) electrostatic potential, which are preferred sites for electrophilic attack; blue indicates regions with the most positive (repulsive) electrostatic potential, which are favorable for nucleophilic attack; and green indicates regions with zero (neutral) potential. The MEP surfaces of the molecules named in the title were generated using the TPSSh method.

**Figure 6:**
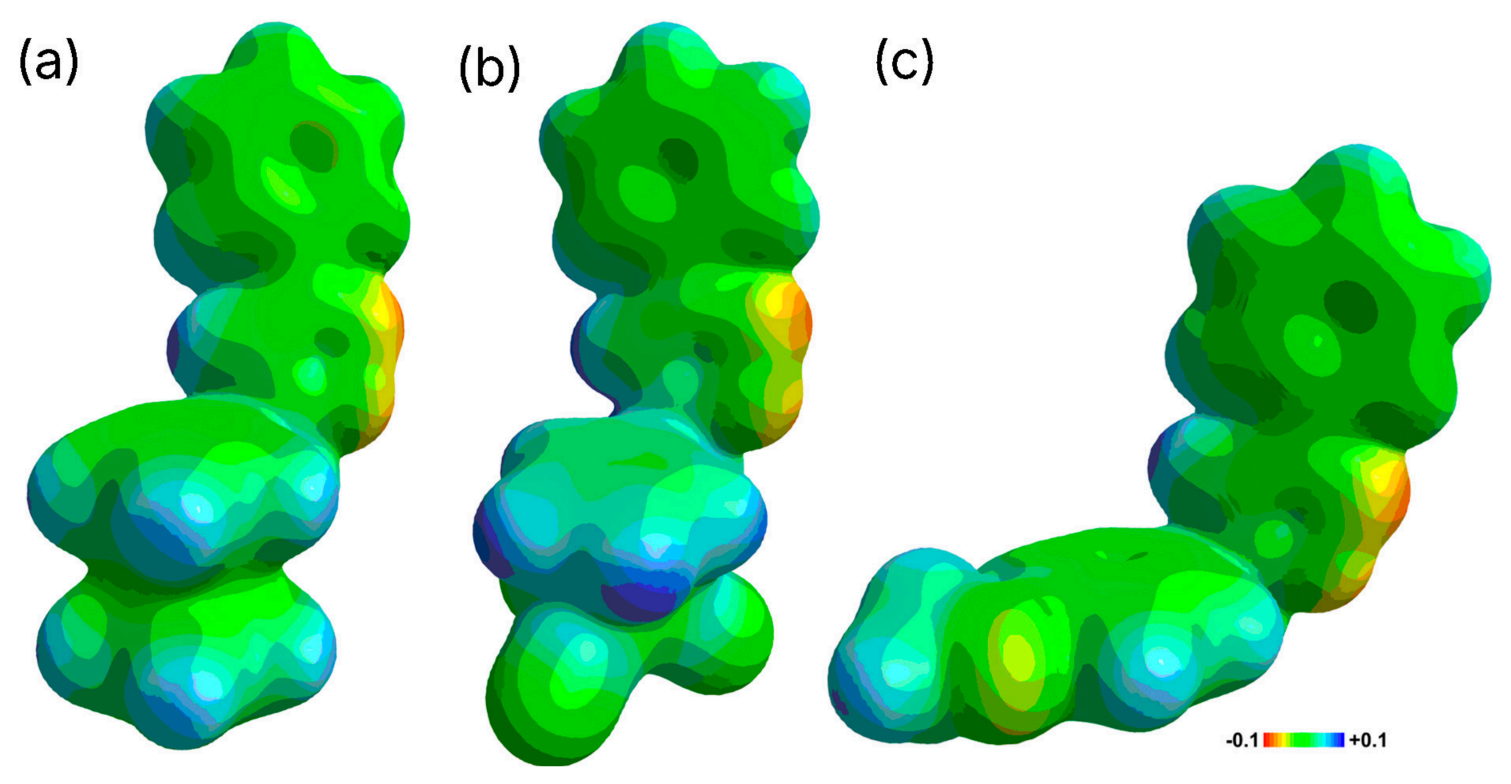
MEP map for connections **1** (**a**), **3** (**b**), and **5** (**c**).

Figure 6 shows that the positive (blue) region favorable for nucleophilic attack was located around the hydrogen atoms of the methyl or ethyl linker connecting the aromatic substituent (organometallic (**1**–**4** and **6**) or organic (**5**)) to the triazole ring. It is worth noting that, for cymantrenyl derivatives **3** and **4**, the positive electrostatic potential of the linker was the highest, which is likely due to the electron-withdrawing properties of the organometallic moiety. In cases **3** and **4**, the carbon atoms of the CO ligands also served as favorable sites for nucleophilic attack, indicating the possibility of ligand exchange at the manganese atom. For all triazoles **1**–**6**, the maximum negative charge (red) was concentrated around the nitrogen atoms in the heterocyclic ring. In the case of 1,2,3-triazoles, the electron density was shifted—as expected—toward the *N3* position, making this site the most likely target for electrophilic attack. Compound **6** exhibited the lowest MEP value at the nitrogen atoms; consequently, its reactivity was reduced compared to the organometallic derivatives, a factor that may influence its biological activity. Thus, the 1,2,3-triazoles we obtained can act as electrophiles or nucleophiles, demonstrating the potential to develop anticancer agents capable of inhibiting cancer cell growth through multiple mechanisms of action [21].

### 2.5. Photochemical Properties of Triazoles ***3*** and ***4***

**Scheme 1:**
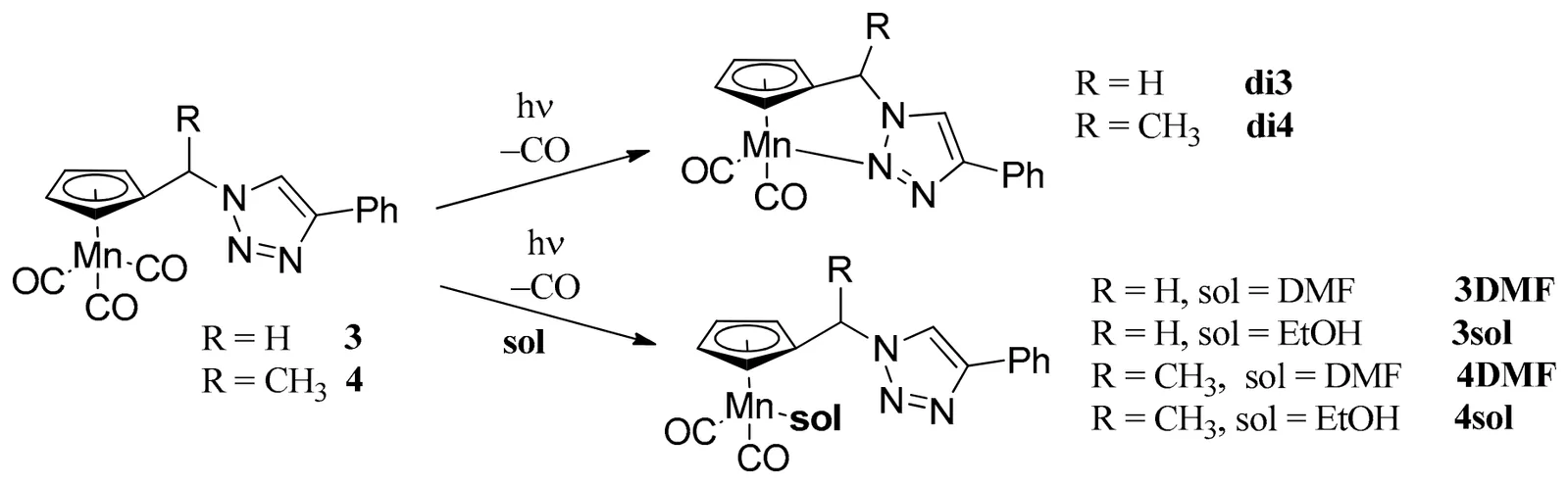
Products of photolysis of triazoles **3** and **4** in different solvents.

We have previously shown [46] that compounds **3** and **4** underwent a photoinduced ligand exchange reaction when irradiated with a mercury lamp at λ = 365 nm in several solvents with different abilities to form complexes with the manganese atom of cymantrene derivatives (benzene, THF, DMF, and cyclohexane). It was established that, upon elimination of the CO ligand, two types of complexes can be formed: a chelate complex with anMn–2N bond to the triazole ring ([1-(η^5^-cyclopentadienyl)methyl-4-phenyl-κ2N-1,2,3-triazole](dicarbonyl)manganese (**di3**) and {1-[1-(η^5^-cyclopentadienyl)ethyl]-4-phenyl-κ2N-1,2,3-triazole}(dicarbonyl)manganese (**di4**)) and an intermolecular dicarbonyl complex in which the solvent molecule is coordinated to manganese ([1-(η^5^-cyclopentadienyl)methyl-4-phenyl-1,2,3-triazole](dicarbonyl)(DMF)manganese (**3DMF**) and {1-[1-(η^5^-cyclopentadienyl)ethyl]-4-phenyl-1,2,3-triazole}(dicarbonyl) (DMF)manganese (**4DMF**)) (Scheme 1). The ratio of the complexes immediately after irradiation depended on the n-donor capacity of the solvent and on the nature of the substituent in the α-position of the Cp ring; in a number of cases, we did not observe the formation of intermolecular complexes, and spectroscopic signals from only the chelate complex were observed in the reaction mixture. However, whereas photolysis yielded two types of complexes in the reaction mixture, during the thermal reaction, the intermolecular dicarbonyl complexes were converted into chelate complexes, which were isolated and characterized by X-Ray diffraction. Therefore, to understand the photochemical processes occurring in physiological environments, a study of the photolysis of triazoles **1**–**6** in aqueous–alcohol mixtures was conducted.

Irradiation of aqueous ethanol solutions of triazoles **1**–**2** and **5**–**6** with monochromatic light at λ = 365 nm for up to 10 min did not result in any changes in the IR, UV–vis, or fluorescence spectra or in the cyclic voltammograms. Therefore, **1**–**2** and **5**–**6** were found to be photoinactive molecules.

**Figure 7:**
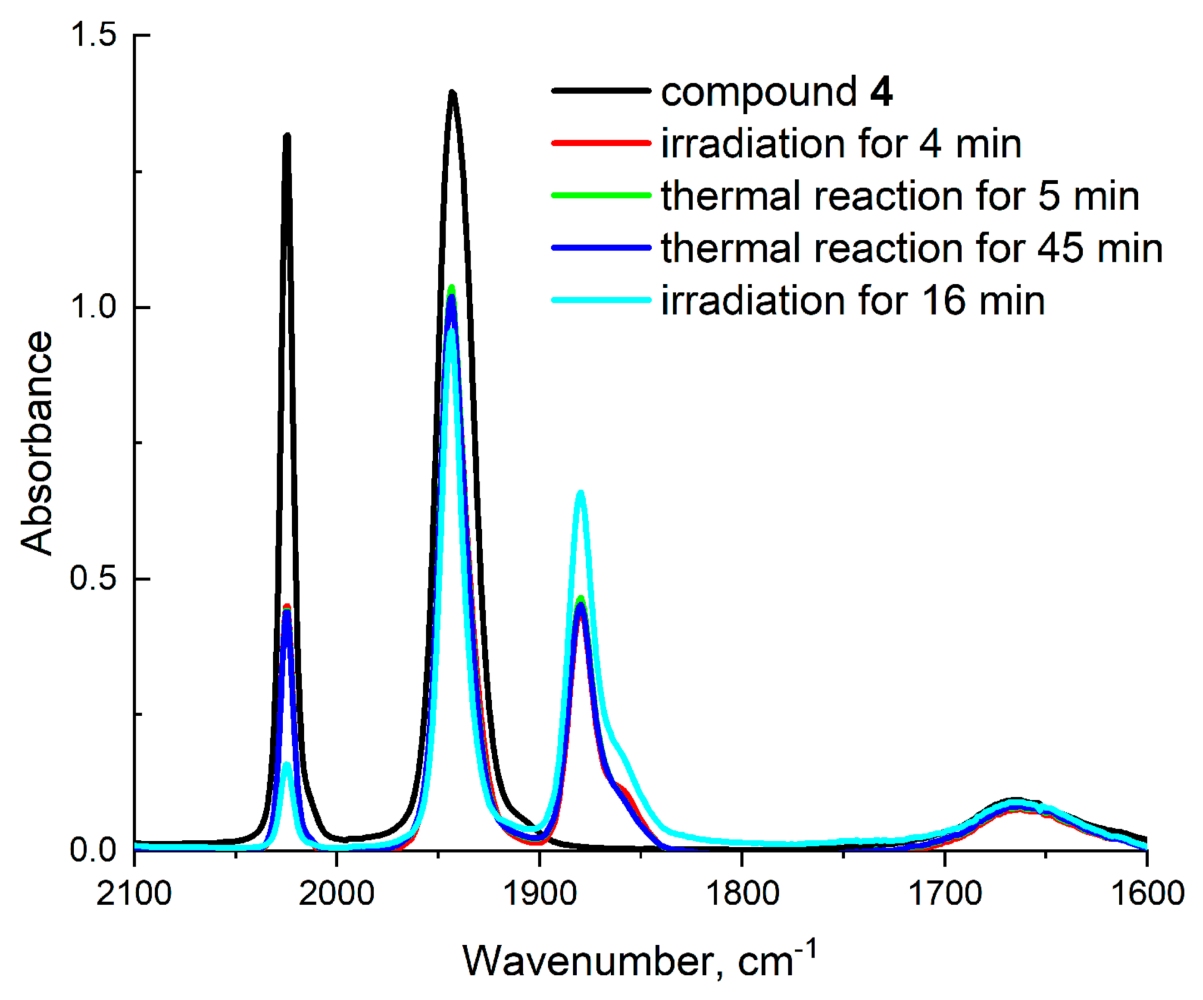
Monitoring the irradiation and thermal reaction of triazole **4** in a 1:1 water–ethanol mixture.

Monitoring the irradiation of cymantrenyl derivatives **3** and **4** in a1:1 water–ethanol mixtures by IR spectroscopy showed that monochromatic light caused, as we had previously shown [46], a decrease in the intensity of the ν(MCO) at 2024 and 1943 cm^−1^ and the appearance of two sets of new bands associated with the CO vibrations of the ligand, at 1944 and 1880 cm^−1^ and at 1931 and 1958 cm^−1^, respectively (Figure 7).

The band assignment was made based on literature data [46,62], and it was shown that the bands at 1944 and 1880 cm^−1^ belong to the chelate complexes with Mn-2N(triazole) bond **di3** and **di4**, while the set of ν(MCO) at 1931 and 1958 cm^−1^ is associated with the dicarbonyl complex with the Mn–O bond of the solvent (alcohol or water)([1-(η^5^-cyclopentadienyl)methyl-4-phenyl-1,2,3-triazole](dicarbonyl)(solvent)manganese (**3sol**) and {1-[1-(η^5^-cyclopentadienyl)ethyl]-4-phenyl-1,2,3-triazole}(dicarbonyl) (solvent)manganese (**4sol**)) (Scheme 1). It is worth noting that the positions of the bands did not depend on the nature of the cymantrenylalkyl fragment, but the introduction of a methyl group reduced the amount of dicarbonyl complex formed with the Mn–solvent bond. In both cases, after just 5 min of the thermal reaction, the bands from vibrations associated with **3sol** and **4sol** were practically unobservable, while the intensities of the **di3** and **di4** bands increased during this time and then remained practically unchanged even after 45 min at room temperature in the dark. Therefore, all subsequent spectroscopic data for irradiated solutions were recorded 5 min after photolysis of the aqueous–ethanol solutions. Increasing the irradiation duration led to a further decrease in the ν(MCO) bands of the tricarbonyl complex and also changed the ratio of the bands for the dicarbonyl complexes **di3**, **di4**, **3sol**, and **4sol**. Thus, in case **3**, after 2 min of irradiation, the intensity ratio of the **di3** and **3sol** bands was 1.2, and with increasing photolysis time, it rose to 1.6, which may be due to accelerated ligand exchange for **3sol** during irradiation, leading to an additional increase in the amount of **di3** in the reaction mixture. Thus, the irradiation of cymantrenylalkyl triazole derivatives proceeded through the elimination of one CO ligand to form a 16-electron manganese center, which could then be stabilized by coordination with both the solvent and the heterocycle nitrogen atom.

Ligand exchange then occurred at the manganese center, leading to the thermodynamically more stable chelates. It should be noted that upon irradiation of 11.7 µmol of tricarbonyl complexes **3** and **4** in a1:1 water–ethanol mixture for 4 min, the estimated minimum amounts of CO released were 8.0 and 7.7 µmol, respectively.

The structures of the chelate complexes **di3** and **di4** were further examined using electrospray ionization mass spectrometry. Spectra were obtained in positive-ion mode after 2 and 4 min of irradiation (Figures S19 and S20). In contrast to the spectra of the initial triazoles **3** and **4** ([M + H]^+^), the principal ions detected after irradiation were [M – 2CO + CH_3_CN]^+^ and [M – 2CO]^+^, originating from the dicarbonyl complexes **di3** and **di4** with an Mn–N(triazole) bond.

**Figure 8:**
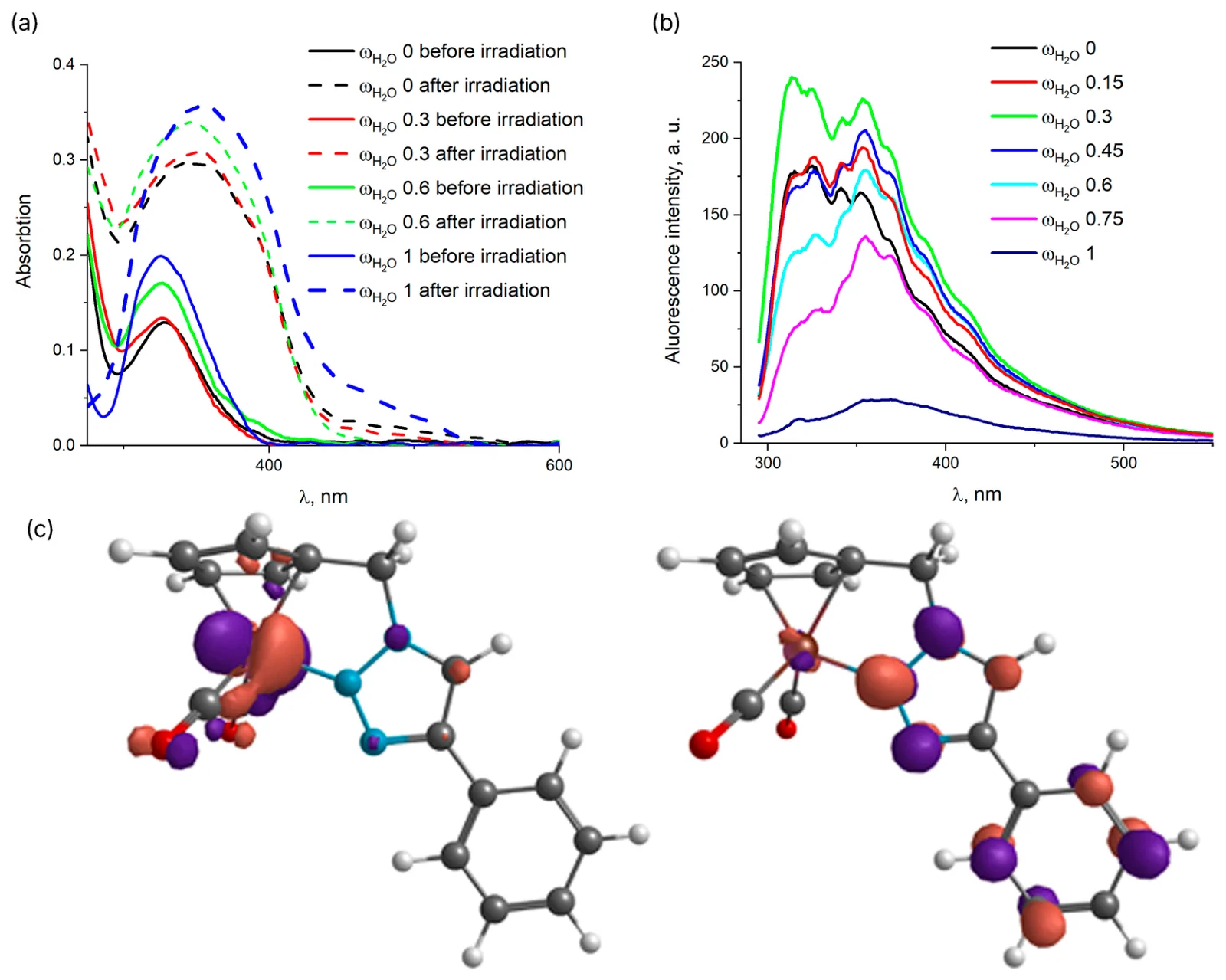
(**a**) UV–vis spectra of **3** before (solid line) and after (dashed line) at different water contents. (**b**) Fluorescence spectra of **di3** at different water contents. (**c**) NTO analysis of **di3**.

A UV–visible spectroscopic study of the photolysis of aqueous ethanol solutions of compounds **3** and **4** revealed that the position of the first low-frequency absorption band at 327 nm depended weakly on the ethanol-to-water ratio. However, the extinction increased by a factor of 1.6 upon changing the solvent from pure ethanol to water, which may be due to an increase in the oscillator strength of a possible MLCT transition in a polar medium, as discussed previously in Chapter 2.4 (Figure 8a). When solutions **3** and **4** were irradiated, a new absorption band emerged at 349 nm. Their extinctions also depended on the ethanol:water ratio, but to a lesser extent than those of the tricarbonyl complexes. It is worth noting that the absorption maxima of the **di3** and **di4** bands were weakly dependent on the proportion of water in the solution. For the 349 nm band, a 5 nm shift toward lower energies was observed upon moving from pure ethanol to water, which may be due to an increase in solvent polarity. This confirmed our earlier conclusion [46] that charge-transfer complexes are formed during irradiation. This assumption was also confirmed by quantum chemical calculations using the TPSS method. The geometries of the dicarbonyl chelate complex **di3** were optimized, UV–visible spectra were calculated using TD-DFT (Figure S23, Tables S6 and S7), and NTO (Figure 8c, Table S8) analysis was performed. Analysis showed that 96.8% of the absorption band at 393.4 nm (corresponding to the experimental band at 349 nm) consists of electron transfer from an orbital where the electron density is primarily located in the manganese *d* orbital to the delocalized triazole-phenyl π* orbital (Figure S23).

Upon irradiating aqueous–ethanol solutions of triazoles **3**–**4** and forming the dicarbonyl chelates **di3**–**di4**, the fluorescence spectra remained unchanged compared to those of the tricarbonyl complexes. However, the band intensity decreased by a factor of 1.1 in the 295–600 nm range at all ethanol:water ratios (Figure 8b). Consequently, the formation of the dicarbonyl complexes did not result in the emergence of new energy levels or changes in existing energy levels from which emission processes could occur. The formation of the charge-transfer complex apparently resulted in partial energy transfer from the triazole ligand to the metal atom and subsequent nonradiative recombination, which was responsible for the decrease in fluorescence intensity. Thus, fluorescence spectroscopy can be used to monitor the chemical and biochemical transformations of triazoles containing the cymantrenyl moiety under the influence of various external factors, including radiation.

**Figure 9:**
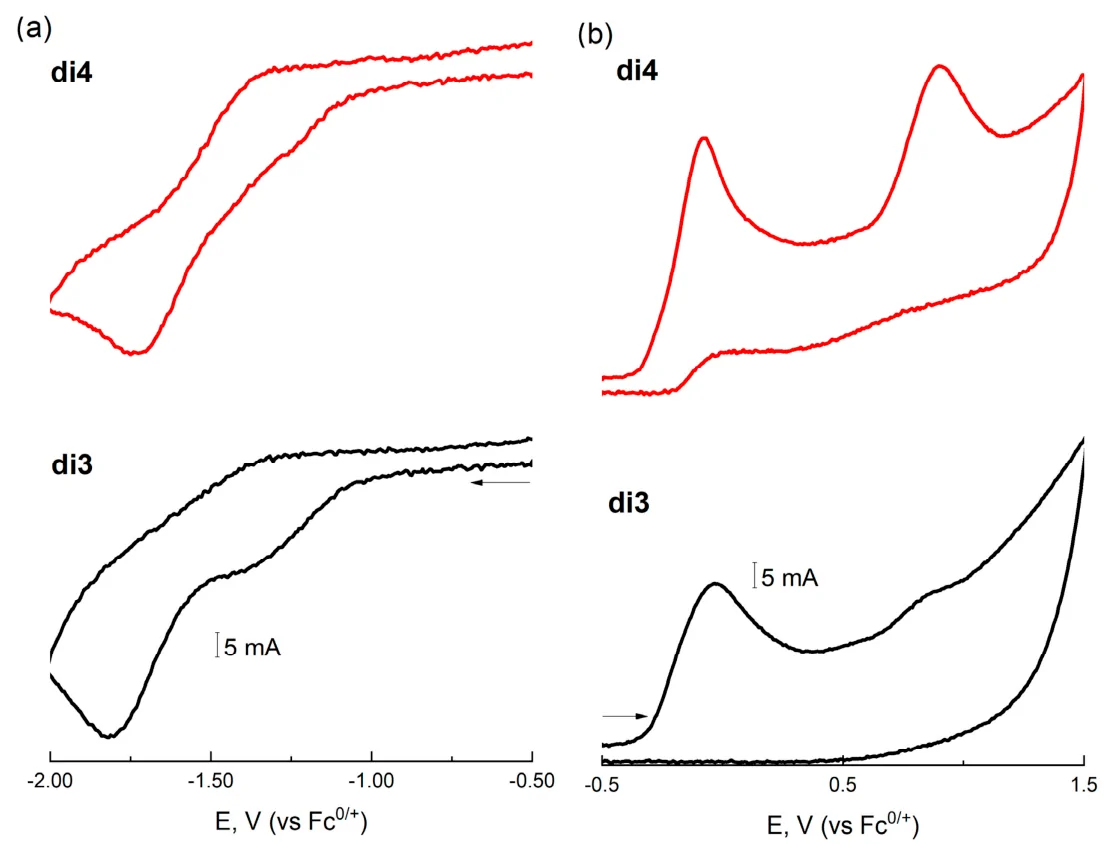
Cyclic voltammograms of **di3** and **di4** in a 0.1 M tetraethylammonium tetrafluoroborate solution in ethanol: (**a**) oxidation branch and (**b**) reduction branch. Scan rate: 100 mV/s.

Photolysis monitoring of solutions **3** and **4** revealed the emergence of new irreversible single-electron oxidation peaks at −0.04 V and −0.08 V, respectively (Table 2 and Figure 9a). This change in the oxidation branch was associated with the formation of chelate complexes involving the Mn–N bond in the triazole ring. Sharp changes in the reduction branch were also observed. Thus, the chelate complex **di3** exhibited two consecutive irreversible reduction peaks with a total transfer of two electrons, the first reduction being more rapid than that of the tricarbonyl complex **3** (Figure 9b). In the case of chelate **di4**, two reduction peaks were also observed, the first irreversible and the second quasi-reversible. At the same time, the energy gaps of the chelates **di3** and **di4** decreased sharply to 0.73 and 0.71, respectively, compared with those of **3** and **4**. Thus, the transition from tricarbonylcymantrene complexes to dicarbonyl complexes yielded compounds with lower oxidation potentials and, overall, enhanced reactivity. In terms of ease of oxidation, the chelates **di3** and **di4** were superior to the ferrocenyltriazoles **1**–**2** and **6**. Irradiation is likely an effective method for regulating the properties of cymantrene derivatives.

### 2.6. Anticancer Activity of Triazoles ***1**–**6***

The cytotoxicity of compounds **1**–**6** was studied in three cell lines: A549 (human non-small-cell lung carcinoma), HCT116 (human colorectal cancer), and MCF-7 (breast cancer), using the MTT method. Initially, the concentration-dependent activity of triazoles **1**–**6** was studied under dark conditions (Table 4). The lack of a reference cytotoxin and of a non-malignant control should be stated openly as limitations. Cytotoxicity analysis of triazole-class compounds on A549, HCT116, and MCF7 cell lines revealed that compound **4** exhibited the most pronounced activity, effectively inhibiting the growth of A549 (CC50 = 9.0 ± 0.6 µM) and HCT116 (CC50 = 15.9 ± 2.2 µM) cells. Compounds **2** and **5** also demonstrated significant, albeit cell-line-specific, toxicity: compound **5** was most active against A549 cells (CC50 = 12.0 ± 1.5 µM), whereas compound **2** was most active against MCF7 cells (CC50 = 18.3 ± 5.8 µM). In contrast, compounds **1** and **3** did not exhibit significant cytotoxicity against any of the cell lines (CC50 > 300 µM), whereas compound **6** demonstrated differential activity among several cancer cell lines toward HCT116 (CC50 = 37.0 ± 13.3 µM). The effects of UV irradiation and incubation time were inconsistent: UV irradiation enhanced the toxicity of compounds **5** and **6** against HCT116 cells, while it reduced the toxicity of compound **5** against A549 and MCF7 cells and compound **2** against MCF7 cells. The obtained data allow us to recommend compound **4** as the most promising for further research and compounds **2**, **5**, and **6** as candidates for targeted therapy of the corresponding tumor cell lines.

Thus, physicochemical properties are not the sole determinants of the biological activity of compounds. This is primarily because simple systems—in which electronic, optical, and electrochemical properties are studied—cannot reflect the full range of processes occurring in complex biological objects. Based on assessments of its primary capabilities, compound **6** should exhibit biological activity against A549 and MCF7 cell lines in the absence of light, yet it showed no cytotoxicity toward these cell lines. This behavior may be due to the fact that the biomolecules responsible for cellular homeostasis either cannot form stable bonds with triazole **6** or form such conjugates without inhibiting their biological functions. However, based on an analysis of the obtained data, it can be suggested that biological activity against A549 and MCF7 of organometallic compounds correlates, to some extent, with the ease of reduction in the triazoles. Unlike organometallic derivatives, compound **5**—despite its low reduction potential—inhibits the growth of A549 cells in the dark owing to a different mechanism of action. Furthermore, one cannot rule out the possibility that the reduction potential decreases, thereby enhancing the compound’s ability to accept electrons, as a result of the formation of intermolecular interactions with biomolecules. For the photoinactive molecules **2**, **5**, and **6**, an effect of UV irradiation is observed, which may be attributed to the formation of complex compounds with various biomolecules. The influence of intermolecular interactions is also evidenced by the fact that the biological activity of the photoactive triazoles **3** and **4** depends on the incubation time before irradiation. It should also be noted that the introduction of a methyl group at the *1* position of the side chain of the Cp-ring has the greatest impact on biological activity. Investigating the role of naturally occurring metals (Mn or Fe) in the cytotoxicity of triazoles is a complex task that requires further research. Therefore, to correlate the properties of organometallic compounds with their biological activity and to predict their mechanism of action, it is necessary to consider intermolecular interactions with various biomolecules and the resulting changes in their reactivity. This hypothesis requires further careful study.

## 3. Materials and Methods

^1^H and ^13^C NMR spectra (internal standard—solvent, δ from Me_4_Si, acetone—2.05, chloroform—7.26) were recorded on an Avance 400 spectrometer (Bruker, Karlsruhe, Germany), 400.13 and 100.61 MHz, respectively. High-resolution mass spectra were obtained using a hybrid quadrupole time-of-flight mass spectrometer (LCMS-9030, Shimadzu, Kyoto, Japan) equipped with an electrospray ionization (ESI) source. All spectra of compounds **1**–**6** were recorded under identical conditions: samples were dissolved in 100% methanol (C = 5 × 10^−4^ M) and introduced into the ESI source via direct injection using an LC-40 Nexera (Shimadzu, Kyoto, Japan) HPLC system. The mobile phase was a mixture of 100% methanol; the flow rate was set to 0.4 mL/min. Mass spectra were acquired in positive ion mode over the *m*/*z* range of 100–500. The following ESI source parameters were used: capillary voltage, 4.5 kV; interface temperature, 300 °C; desolvation line (DL) temperature, 250 °C; heat block temperature, 300 °C. N_2_ flows were set to 3.0 L/min for the nebulizing gas and 10.0 L/min for both the drying and heating gases. The mass spectrometer was calibrated using a solution of sodium iodide in methanol/water. Data acquisition and instrument control were managed using LabSolutions software (version 5.114, Shimadzu). UV–vis absorption spectra were recorded on a Carry 300 spectrophotometer (Agilent, Santa Clara, CA, USA). Solid-state UV–visible spectra were recorded from solid powder samples diluted in vaseline oil and placed between two quartz glass plates, each 0.1 cm thick. Fluorescence spectra were recorded on a Carry Eclipse (Agilent, Santa Clara, CA, USA). Fluorescence quantum yields were determined using fluorescein in 0.1 N NaOH as a standard (Φ = 0.90). The relative quantum yield was determined. Quantum yields were corrected using the instrument software. IR vibrational spectra of the solutions were recorded at room temperature (24 °C) using an FTIR absorption spectrometer (SHIMADZU IRTracer-100, Kyoto, Japan). Each sample spectrum was recorded from 400 to 4000 cm^−1^ with a spectral resolution of 2 cm^−1^ and averaged over 32 signal scans. Electrochemical studies were conducted in a standard glass cell with interchangeable electrodes, using an IPC-PRO potentiostat (Volta, Saint-Petersburg, Russia) connected to a personal computer. The working electrode was a glassy carbon disk with an area of 0.03 cm^2^, a larger platinum plate served as the counter electrode, and potentials were measured against a saturated silver chloride electrode. Measurements were performed in a 0.1 M ethanol solution of tetraethylammonium tetrafluoroborate, using the Fc/Fc^+^ pair in accordance with the standard. Photochemical transformations were performed using a TQ 150 immersion mercury lamp (Heraeus, Hanau, Germany) equipped with an S49 glass jacket for cooling. Monochromatic radiation with a wavelength of 365 nm was obtained by passing the lamp light through UVS-6 and BS-7 filters. The reaction progress and purity of the obtained compounds were monitored by analytical thin-layer chromatography on Silufol UV-245 plates (Kavalier, Votice, Czech Republic). Silica gel 60 (Merck, Darmstadt, Germany) was used for column chromatographic separation. Commercially available organic solvents of analytical grade were used in the work. The synthesis of 4-substituted 1,2,3-triazoles **1** and **3**–**6** was described previously [31,32].

**General procedure for synthesis of triazoles 1–5**. To a suspension of CuSO_4_·5H_2_O (5 mmol) and sodium ascorbate (15 mmol) in distilled water (10 mL) was added t-BuOH (20 mL), and the resulting mixture was stirred at room temperature under argon for 15 min. The dark red solution turned sandy in color. Solutions of the corresponding azide (20 mmol) in acetonitrile (10 mL) and phenylacetylene (30 mmol) in acetonitrile (10 mL) were then successively added, and the reaction mixture was stirred at room temperature for 24 h. Then the reaction mixture was passed through a silica gel layer. The filter cake was washed with ethyl acetate, and the filtrate was concentrated. The residue was crystallized from acetonitrile.

**1-(1-Ferrocenylethyl)-4-phenyl-1H-1,2,3-triazole (2)** [63]. Yield 85%. Light yellow powder, mp 165–166 °C. ^1^H NMR (400 MHz, CDCl_3_, δ, ppm, J/Hz): 1.93 (d, J = 7.0, 3H, CH_3_), 4.18 (s, 5H, Fc), 4.20 (m, 2H, Fc), 4.24 (m, 1H, Fc), 4.32 (m, 1H, Fc), 5.77 (q, J = 7.0, 1H, CH), 7.30 (t, J = 7.2, 1H, Ph), 7.38 (t, J = 7.5, 2H, Ph), 7.55 (s, 1H, CH), 7.77 (d, J = 7.1, 2H, Ph). ^13^C NMR (101 MHz, CDCl_3_/(CD_3_)_2_CO, δ, ppm): 21.2 (CH_3_), 56.4 (CH), 65.9 (Cp), 67.7 (Cp), 68.1 (Cp), 68.7 (Cp), 68.8 (Cp), 87.3 (C_i_-Cp), 117.4 (Trz), 125.4 (Ph), 127.7 (Ph), 128.5 (Ph), 130.7 (Ph), 147.0 (Trz). HRMS (ESI): C20H19FeN3, *m*/*z* calcd for [M]^+^ 357.0923, found 357.0935. Found, %: C 67.04, H 5.26, N 11.90, Fe 15.8 for C20H19FeN3. Calculated, %: C 67.24, H 5.36, N 11.76, Fe 15.63.

**General method of spectral studies of photochemical reactions of triazoles 1–6.** Solutions of compounds **1**–**6** in water–ethanol solutions with varying water content (C = (2–4) × 10^−3^ mol/L were placed in IR or UV cells under an argon atmosphere and irradiated with light at λ = 365 nm from a mercury lamp at room temperature. The lamp was switched on 2 min before irradiation, and the spectra were recorded every 2–4 min. The samples were irradiated until 50–100% conversion of the tricarbonyl complexes was achieved.

**General procedure for determining the amount of CO released.** Compounds **3** and **4** (11.7 µmol) were dissolved in 0.2 mL of a 1:1 water–ethanol mixture. The solution was then placed in a cuvette, and its IR spectrum was recorded. The solution was irradiated with 365 nm light for 4 min in the IR cuvette, and the IR spectrum was recorded again. Based on the IR spectra obtained and the Beer–Lambert–Bouguer law, the extinction coefficients and the amounts of unreacted starting compounds were determined. The minimum amount of CO released was equal to the amount of compound **3** or **4** that had undergone photolysis over the 4 min period.

**X-Ray crystallography.** Single crystals suitable for X-Ray structural analysis were grown by slow evaporation of a chloroform solution of the complex. Data for compound **2** were collected at 120 K on a Bruker APEXII DUO CCD diffractometer using Mo-Kα radiation (λ = 0.71073 Å) passed through a graphite monochromator. The structures were solved using the Olex2 software package (version 1.5) [64] with intrinsic phasing method in the ShelXT program [65]. Subsequently, the structures were refined using the XL program [66] via the least-squares method based on the squares of the structure amplitudes (F^2^), with an anisotropic approximation applied to all non-hydrogen atoms. The coordinates of the hydrogen atoms were determined computationally, and their refinement was carried out isotropically using a riding model. Crystallographic parameters and structure refinement data are presented in Table S2. Additional information regarding the crystallographic data obtained in this work has been deposited with the Cambridge Crystallographic Data Center (CCDC) under deposition number 2586079 for compound **2**.

**Computational Methods.** Geometry optimizations were carried out without symmetry constraints using the TPSSh [67,68] density functional and tight convergence criteria as implemented in the ORCA 5.0.4 program package [69]. Tight SCF convergence criteria and the default tight “DEFGRID3” integration grid were used. The Karlsruhe orbital basis set def2-TZVPP was utilized [70]. The RIJCOSX approximation for the Hartree-Fock exchange [71] was used together with the appropriate auxiliary basis set def2/J [72]. The atom-pairwise dispersion correction to the DFT energy with Becke-Johnson damping (D3BJ) was applied [73,74]. Analytical frequency calculations were performed to confirm the nature of the stationary points and to yield no imaginary frequencies.

The choice of the TPSSh functional was motivated by the geometry benchmark for the first-row transition metal complexes [75]. With the bond lengths mean absolute deviations of 0.005 Å and 0.017 Å for **3** and **di3**, respectively, the chosen approach provides structures of the quality comparable to those from the classic hybrid [75] or recently developed methods like r2SCAN-3c [76] (Table S3).

The energy evaluation was performed in a single-point fashion on the obtained geometries using the TPSS density functional [67] and RI approximation. The HOMO-LUMO gaps and electrostatic potentials were obtained from this evaluation. The basis sets (def2-TZVPP and def2/J), SCF convergence criteria, and integration grid were chosen the same as for geometry optimizations. The electrostatic potential was analyzed using the Multiwfn program package (development version 3.8 of 5 May 2025) [77,78] and visualized with ChemCraft software (version 1.8) [https://www.chemcraftprog.com].

Absorption spectra were computed within the TD-DFT formalism with the Tamm-Dancoff approximation. TPSS/def2-TZVPP level was chosen. Forty roots were requested in all cases. All virtual orbitals were included in the calculations. Only singlet excited states were considered. The solvent corrections were considered using the SMD solvation model [79] and ethanol as a solvent. The spectra were modeled by the convolution of the absorptions with a Gaussian function with specific parameters. Natural transition orbitals were obtained and visualized for selected absorptions in order to analyze the change in electronic structure between states.

Absorption spectrum of di3 was additionally computed in benzene with a set of density functionals (M06-L [80], r2SCAN [81], DSD-PBEP86 [82], SCAN [83], M06 [84], TPSSh, BHANDHLYP [85], CAM-B3LYP [86], B2BLYP [87], B3LYP [88], PBE0 [89], mPW2PLYP [90], TPSS0 [91], PBE0-DH [92], and PBE-QIDH [93]) and the r2SCAN-3c composite method. The calculated MLCT wavelengths were compared with the experimental value in benzene (467 nm) (Table S6). Among the tested methods, TPSS gave the smallest deviation from this data point and was therefore used for the qualitative assignment of the absorption bands in the remainder of the work. This benchmark, however, relies on one compound and one experimental band, and the remaining errors are substantial: TPSS places the band in benzene at 406 nm (ca. 0.4 eV blue-shifted relative to experiment). In ethanol, the same approach assigns the experimental band at 349 nm to a state calculated at 393 nm (ca. 0.4 eV red-shifted). The experimentally observed solvatochromic shift between benzene and ethanol is therefore not reproduced. The computed excitation energies should be regarded as a qualitative guide to the character of the transitions, and not as quantitatively predictive. The relatively better performance of local functionals compared to double hybrids (with the exception of DSD-PBEP86) is consistent with reports for other organometallic complexes [94], but, given the above limitations, we do not draw more general conclusions from it.

**Cell Culture.** A549, MCF7, and HCT116 cells were grown in the DMEM:F12 (2:1) medium supplemented with 10% FBS (BioSera, Cholet, France), 2 mM L-glutamine, 100 U/mL penicillin, and 100 μg/mL streptomycin in the presence of 5% CO2 at 37 °C. Cells were re-seeded every three days at a ratio of 1:3 or 1:5. STR profiling was performed in 2024; cell lines were tested for mycoplasma contamination before use.

**Compound testing.** Stock solutions were diluted in DMSO (BioReagent for Molecular Biology grade, Sigma, St. Louis, MO, USA). After 24 h incubation, the studied compounds at different concentrations were added to the culture medium. After addition to the cells, the lidded plate was exposed to 365 nm UV light at a distance of 20 cm for 2 min. Incubation prior to UV exposure was carried out for the indicated time intervals. The final concentration of DMSO was 1%; the vehicle control was 1% DMSO in medium. Cell viability was then evaluated using the MTT test.

**MTT test.** After the incubation at 37 °C in 5% CO2 for 72 h, a freshly prepared solution of thiazolyl blue tetrazolium bromide at a final concentration of 0.4 mg/mL was added to each well of the plate. Incubation was performed at +37 °C for 2 h. The medium containing unreacted MTT was removed by aspiration. To dissolve the formazan formed by the reaction, 100 µL of isopropanol was added to each well of the plate. The result of the analysis was evaluated using a BioradiMark spectrophotometer (absorbance at 595 nm). There were three technical replicates and one biological replicate (two for non-UV experiments to check reproducibility); errors were presented as mean ± SD. Irradiation was performed using a Vilber Lourmat VL-215.LC UV lamp (15 W per light source). The irradiance of the lamp at a distance of 20 cm was approximately 1 mW/cm^2^. CC50 values were fitted by calculating the crossing point between the curve and the 50% line.

## 4. Conclusions

During the study, the structural, photophysical, electrochemical, and electronic properties of 4-phenyl-1,2,3-triazoles containing an organometallic substituent at position 1 were systematically investigated, and a comparison was made with an organic analog and a 2,4-substituted derivative. It was shown that the introduction of the organometallic moiety led to an increase in fluorescence intensity compared to the organic analog. The fluorescence of triazole derivatives depended on medium viscosity and on molecular aggregation in solution. Electrochemical processes, such as oxidation and reduction, were determined not only by the nature of the organometallic fragment but also by its position within the heterocyclic fragment and the possibility of intramolecular interactions between the metal atom and the nitrogen atom of the heterocycle. Determination of the electronic properties using experimental and computational methods revealed that ferrocenyl derivatives were the most reactive compounds. However, reactions with soft nucleophiles were facilitated by the introduction of a cymantrenylalkyl substituent. A study of the photochemical behavior of 1,2,3-triazoles revealed that only cymantrene derivatives were capable of photochemical reactions involving ligand exchange at the manganese atom. When irradiated in a water–ethanol mixture of cymantrenyl derivatives, the formation of two types of complexes was observed: chelates with a Mn-N triazole bond and dicarbonyl complexes with a Mn-solvent bond. The chelate complex is more thermodynamically stable. The formation of chelate complexes led to changes in the photophysical and electrochemical properties, as well as an increase in the reactivity and oxidant potentials of the cymantrene alkyl derivatives. The biological activity of organometallic triazoles against three cancer cell lines—A549, HCT116, and MCF7—depended on the reduction potentials. Thus, among all the compounds studied, 1-(cymantrenylethyl)-4-phenyl-1,2,3-triazole (**4**) exhibited the highest cytotoxicity (A549 (CC50 = 9.0 ± 0.6 µM) and HCT116 (CC50 = 15.9 ± 2.2 µM)). The effect of UV irradiation was ambiguous and requires further, more thorough investigation to elucidate the mechanism of action. For the first time, the water–ethanol fluorescence, electrochemical, electronic, and anticancer properties of cymantrenylalkyl and ferrocenylalkyltriazoles were studied and confirmed by DFT calculations. It was shown that cytoactivity changes under UV irradiation.

Their mechanism of action, as well as the influence of various biomolecules, requires further thorough study. To construct an accurate predictive model of biological activity and determine the structure–activity relationship, it is necessary not only to thoroughly study the intermolecular interactions between triazoles and the main biomolecules in cancer cells, as well as the effects of binding on the physicochemical properties of these biomolecules, but also to expand the range of compounds under study. Thus, it is of interest to study the influence of the nature of the substituents at position 1 of the side chain or on the phenyl ring at position 4 of the 1,2,3-triazole ring.

## Figures and Tables

**Table 1:** Photophysical properties of triazoles **1**–**6**.

Compound	λ_Abs_^solid^	λ_Abs_^EtOH^	λ_Em_^EtOH^	Stokes Shift (nm)	Φ	E_g_^opt^, eV
**1**	438	270, 418	356	86	0.32	2.57
**2**	444	269, 418	356	87	0.36	2.55
**3**	307, 328	268, 329	358	90	0.33	3.34
**4**	333	272, 329	356	84	0.37	3.27
**5**	<300	283, 305, 318	356	73	0.24	3.92
**6**	419	271, 419	356	85	0.34	2.55

**Table 2:** Electrochemical parameters for compounds **1**–**6**.

**Compound**	**E_red1_, V**	**E_red2_, V**	**E_ox1_, V**	**E_ox2_, V**	**IP ^1^, eV**	**EA ^1^, eV**	E_g_ ^1^, eV
**1**	−1.75	-	0.46 (0.98)	-	5.51	4.21	1.30
**2**	−1.39	−2.15	0.10 (0.61)	0.35	5.56	4.34	1.22
**3**	−1.44	-	1.09	-	5.92	4.54	1.38
**4**	−1.36	−1.72	0.96	-	5.87	4.51	1.36
**5**	−1.83	-	1.38	-	6.27	4.05	2.22
**6**	−1.89	-	0.10	-	5.46	4.21	1.25
**di3**	−1.39	−1.81	−0.04		4.78	4.05	0.73
**di4**	−1.24	−1.72 (0.6)	−0.08		4.77	4.06	0.71

^1^ IP = |e|(E_Oxonset_ + 5.1) [51]; EA = |e|(E_Redonset_ + 5.1) [51]; E_g_ = IP − EA, where e is the elementary electric charge.

**Table 3:** Calculated reactivity parameters of triazoles **1**–**6**.

Compound	1	2	3	4	5	6
E_g_	3.1725	3.1647	3.7154	3.6715	4.0152	2.9154
χ	3.1506	3.1461	3.8151	3.8106	3.4195	3.0475
η	1.5863	1.5824	1.8577	1.8358	2.0076	1.4577
σ	0.6304	0.6320	0.5383	0.5447	0.4981	0.6860
μ	−3.1506	−3.1461	−3.8151	−3.8106	−3.4195	−3.0475
ζ	0.3152	0.3160	0.2692	0.2724	0.2491	0.3430
ω	3.1288	3.1275	3.9175	3.9549	2.9122	3.1856
ΔN_max_	1.9862	1.9882	2.0537	2.0757	1.7033	2.090

**Table 4:** CC50 (μM) of triazoles **1**–**6** after 72 h of incubation.

**Compound, A549 Cell Line**	**Incubation with the Compound Prior to UV Irradiation, min**				
	**Without UV**	**0**	**2**	**4**	**10**
**1**	>300	>300	>300	>300	>300
**2**	205.0 ± 2.0	22.0 ± 1.7	24.8 ± 1.1	17.3 ± 3.7	81.0 ± 3.0
**3**	>300	>300	>300	57.2 ± 31.9	>300
**4**	9.0 ± 0.6	11.7 ± 1.5	27.0 ± 9.6	15.2 ± 3.3	11.0 ± 2.8
**5**	12.0 ± 1.5	49.0 ± 1.3	51.5 ± 1.7	42.5 ± 6.0	53.8 ± 2.5
**6**	>300	61.7 ± 16.9	40.8 ± 14.9	27.5 ± 4.3	73.0 ± 24.9
**Compound, HCT116 Cell Line**	**Incubation with the Compound Prior to UV Irradiation, min**				
	**Without UV**	**0**	**2**	**4**	**10**
**1**	>300	>300	>300	>300	>300
**2**	122.0 ± 8.0	91.8 ± 5.7	119.3 ± 1.8	110.6 ± 28.3	147.3 ± 15.7
**3**	>300	>300	>300	>300	>300
**4**	15.9 ± 2.2	11.8 ± 1.2	10.3 ± 1.8	13.1 ± 2.7	11.1 ± 3.0
**5**	273.0 ± 18.0	24.0 ± 7.6	172.2 ± 5.4	146.5 ± 61.5	156.5 ± 34.7
**6**	37.0 ± 13.3	13.5 ± 0.7	16.0 ± 3.2	17.3 ± 6.2	19.3 ± 7.1
**Compound, MCF7 Cell Line**	**Incubation with the Compound Prior to UV Irradiation, min**				
	**Without UV**	**0**	**2**	**4**	**10**
**1**	>300	>300	>300	>300	>300
**2**	18.3 ± 5.8	16.9 ± 1.1	23.4 ± 2.4	149.5 ± 6.5	177.3 ± 17.8
**3**	>300	209.3 ± 17.6	100.0 ± 9,9	>300	>300
**4**	146.1 ± 28.3	23.7 ± 6.1	40.2 ± 1.7	83.5 ± 28.3	123.6 ± 37.8
**5**	60.9 ± 15.1	41.8 ± 7.2	20.2 ± 8.1	103.0 ± 23.3	165.0 ± 12.0
**6**	>300	>300	290.5 ± 10.0	>300	>300

## Data Availability

All data and samples are available from the authors.

## Supplementary Materials

The following supporting information can be downloaded at: https://www.mdpi.com/article/10.3390/molecules31213932/s1. The Experimental part, which describes the characteristics compounds **1**–**6**, **di3** and **di4**. The ^1^H and ^13^C NMR spectra (Figures S1–S12). The Mass spectra (Figures S13–S20). The Additional XRD Data (Tables S1–S5; Figures S21–S22). The Additional computational data (Tables S6–S11; Figures S23–S34).
