# Supplementary material for: Organometallic Triazoles with Phototunable Properties as Cytotoxic Agents for Potential Anticancer Applications
Source: Molecules. 2026 Nov 1;31(21):3932. doi: 10.3390/molecules31213932 (PMC13765030; doi:10.3390/molecules31213932)
Supplement: Supplementary file 1 [file molecules-31-03932-s001.zip › molecules-4620284-supplementary.pdf]

Supporting Information

Table of Content

Experimental part 2
NMR spectra...4
Mass spectra 10
Additional XRD Data 13
Additional Computational Data...16

Experimental part

1-(Ferrocenylmethyl)-4-phenyl-1,2,3-triazole (**1**): ^{1}H NMR (600 MHz, acetone-d_6, δ, ppm, J/Hz): 4.21 (m, 2 H, Cp), 4.23 (s, 5 H, Cp), 4.42 (m, 2 H, Cp), 5.42 (s, 2H, CH_2), 7.30 (m, 1 H, Ph), 7.40 (m, 2 H, Ph), 7.87 (d, J = 8.7 Hz, 2 H,Ph), 8.30 (s, 1 H, CH). ^{13}C NMR (154 MHz, acetone-d6, δ, ppm): 50.35 (1 C, CH_2), 69.45 (2 C, Cp), 69.63 (5 C, C_5H_5), 69.72 (1 C, Cp),83.36 (1 C, C-1 of Cp), 120.84 (1 C, 5-Trz), 126.19 (2 C, 3,5-Ph), 128.52(1 C, 4-Ph), 129.58 (2 C, 2,6-Ph), 132.37 (1 C, 1-Ph), 147.83 (1 C, 4-Trz). MS (APCI): m/z = 344 [M + H]+ . C19 H17 FeN3 (343.21): calcd.C 66.49, H 4.99, Fe 16.27, N 12.24; found C 66.27, H 5.13, Fe 15.9, N12.09. HRMS (EI): $C_{19}H_{17}FeN_3$, *m/z* calcd for $[M]^+$ 343.0766, found 343.0777.

1-(1-Ferrocenylethyl)-4-phenyl-1,2,3-triazole (2). ^{1}H NMR (400 MHz, $CDCl_3$, δ, ppm, J/Hz): 1.93 (d, 3H, CH_3, J = 7.02), 4.18 (s, 5H, Cp), 4.21 (m, 2H, Cp), 4.24 (m, 1H, Cp), 4.33 (m, 1H, Cp), 5.79 (q, 1H, CH, J = 7.01), 7.31 (t, 1H, Ph, J = 7.23), 7.38 (t, 2H, Ph, J = 7.47), 7.55 (s, 1H, CH), 7.78 (d, 2H, Ph, J = 7.65). ^{13}C NMR (101 MHz, $CDCl_3$/acetone-d_6, δ, ppm): 21.2 (CH_3), 56.4 (CH), 65.9 (Cp), 67.7 (Cp), 68.1 (Cp), 68.7 (Cp), 68.8 (Cp), 87.3 (C_i-Cp), 117.4 (Trz), 125.4 (Ph), 127.7 (Ph), 128. 5 (Ph), 130.7 (Ph), 147.0 (Trz). HRMS (EI): $C_{20}H_{19}FeN_3$, *m/z* calcd for $[M]^+$ 357.0923, found 357.0935.

1-(Cymantrenylmethyl)-4-phenyl-1,2,3-triazole (3)^{1}H NMR (400 MHz, acetone-d_6, δ, ppm, J/Hz): 4.96 (m, 2H, Cp),5.30 (m, 2H, Cp), 5.37 (s, 2H, CH_2), 7.32 (tt, 1H, Ph, J1 = 7.4 Hz, J2 = 1.2 Hz), 7.43 (tm, 2H, Ph, J = 7.32 Hz), 7.89 (m, 2H, Ph), 8.47 (s, 1H, CH). ^{13}C NMR (154 MHz, acetone-d6, δ, ppm): 47.80 (1 C, CH_2), 83.67 (2 C, Cp), 86.14 (2 C, Cp),99.30 (1 C, Ci-Cp), 121.36 (1 C, 5-Trz), 126.27 (2 C, 3,5-Ph), 128.70(1 C, 4-Ph), 129.63 (2 C, 2,6-Ph), 132.10 (1 C, 1-Ph), 148.07 (1 C, 4-Trz), 225.55 (3C, CO). HRMS (EI): $C_{17}H_{12}MnN_3O_3$, *m/z* calcd for $[M+H]^+$ 362.0332, found 362.0349.

1-(1-Cymantrenylethyl)-4-phenyl-1,2,3-triazole (4): 1H NMR (400 MHz, acetone-d_6, δ, ppm, J/Hz): 1.94 (d, J = 7.1 Hz, 3 H, CH_3), 4.89 (m,1 H, Cp), 4.92 (m, 1 H, Cp), 5.21 (m, 1 H, Cp), 5.37 (m, 1 H, Cp), 5.73(q, J = 7.1 Hz, 1 H, CH), 7.32 (t, J = 7.4 Hz, 1 H, Ph), 7.42 (t, J =7.4 Hz, 2 H, Ph), 7.90 (d, J = 8.3 Hz, 2 H, Ph), 8.48 (s, 1 H, CH).^{13}C NMR (154 MHz, acetone-d6, δ, ppm): 21.52 (1 C, CH_3), 54.91 (1 C,CH), 81.84 (1 C, Cp), 83.60 (1 C, Cp), 85.37 (1 C, Cp), 85.66 (1 C, Cp),104.70 (1 C, Ci-Cp), 119.90 (1 C, 5-Trz), 126.20 (2 C, 3,5-Ph),128.61 (1 C, 4-Ph), 129.55 (2 C, 2,6-Ph), 132.06 (1 C, 1-Ph), 147.73 (1C, 4-Trz), 225.50 (3 C, CO).). HRMS (EI): $C_{18}H_{14}MnN_3O_3$, *m/z* calcd for $[M+H]^+$ 376.0488, found 376.0507.

1-(4-Methoxybenzyl)-4-phenyl-1,2,3-triazole (**5**).^{1}H NMR (400 MHz, acetone-d_6, δ, ppm, J/Hz): 3.79 (s, 3H, CH3), 5.59 (s, 2H,CH_2), 6.94 (d,J =8.7 Hz, 2H, Ph), 7.32 – 7.48 (m, 5H, Ph), 7.88 (d, J =8.7 Hz, 2H, Ph), 8.32 (s, 1H, Trz-5). ^{13}C NMR (154 MHz, acetone-d6, δ, ppm): 53.86 (C, CH_3), 55.56 (1 C, CH_2), 115.00 (2C, Ph), 121.13 (1 C, 5-Trz), 126.17 (2 C, Ph), 128.56(1 C, Ph), 128.83 (1 C, Ph), 128.85 (1C, Ph), 129.57 (2C, Ph), 130.00 (1C, Ph), 130.45 (2 C, 1-Ph), 132.23 (1C, Ph), 148.15 (1 C, 4-Trz). HRMS (EI): $C_{16}H_{15}N_3O$, *m/z* calcd for $[M+H]^+$ 266.1288, found 266.1303.

2-(Ferrocenylmethyl)-4-phenyl-1,2,3-triazole (**6**): A solution of the 1,2,3-triazole**1**(0.5 g, 1.45 mmol) and methyl iodide (0.9 mL, 14.5 mmol) in acetonitrile was heated at 75 °C for 48 h. To monitorthe reaction, an aliquot of the reaction mixture was concentratedand analyzed by NMR spectroscopy and mass spectrometry. Uponcompletion, the solvent was evaporated from the reaction mixture.Purification of the crude product by columnchromatography (petroleum

ether/ethyl acetate, 2:1) and subsequent crystallization (ethyl acetate) afforded **6** (0.35 g, 70 %yield); m.p. 95–96 °C.1H NMR (400 MHz, acetone-d_6, δ, ppm, J/Hz):4.16 (m,2 H, Cp), 4.17 (s, 5 H, Cp), 4.36 (m, 2 H, Cp), 5.39 (s, 2 H, CH_2), 7.35(m, 1 H, Ph), 7.45 (m, 2 H, Ph), 7.88 (d, J = 8.1 Hz, 2 H, Ph), 8.07 (s,1 H, CH). ^{13}C NMR (154 MHz, acetone-d6, δ, ppm): 55.04 (1 C,CH_2), 69.07 (2 C, Cp), 69.49 (2 C, Cp), 69.53 (5 C, C5 H5), 83.65 (1 C,Ci-Cp), 126.51 (2 C, 3,5-Ph), 129.06 (1 C, 4-Ph), 129.71 (2 C, 2,6-Ph), 131.57 (1 C, 5-Trz), 131.68 (1 C, 1-Ph), 148.12 (1 C, 4-Trz). HRMS (EI): $C_{19}H_{17}FeN_3$, *m/z* calcd for $[M]^+$ 343.0766, found 343.0786.

[1-(η5-cyclopentadienyl)methyl-4-phenyl-κ2N-1,2,3-triazole](dicarbonyl)manganese (di3). ^{1}H NMR (400 MHz, C_6D_6, δ, ppm, J/Hz): 3.30 (s, 2H, CH_2), 3.58 (m, 2H, Cp), 4.80 (m, 2H, Cp), 6.04 (s,1H, CH), 7.05 (t, 1H, Ph, J = 7.3 Hz), 7.12 (t, 2H, Ph, J = 7.8 Hz), 7.52 (d, 2H, Ph, J J = 7.7 Hz). IR, cm-^{1} (ethanol) 1944, 1880. HRMS (EI): $C_{16}H_{15}MnN_4$, *m/z* calcd for $[M\text{-}2CO+CH_3CN]^+$ 318.0677, found 318.0709.

{1-[1-(η5-cyclopentadienyl)ethyl]-4-phenyl-κ2N-1,2,3-triazole}(dicarbonyl)manganese (di4). ^{1}H NMR (400 MHz, C6D6, δ, ppm, J/Hz): 0.76 (d, 3H, CH3, J = 6.6 Hz), 3.57 (m, 1H, Cp), 3.65 (m, 1H, Cp), 3.76 (q,1H, CH, J = 6.6 Hz), 4.83 (m,1H, Cp), 4.87 (m, 1H, Cp), 6.41 (s, 1H, CH), 7.07-7.23 (m, 3H, Ph), 7.54 (d, 2H, Ph, J = 7.4 Hz). IR, cm-^{1} (ethanol) 1944, 1880. HRMS (EI): $C_{17}H_{17}MnN_4$, *m/z* calcd for $[M\text{-}2CO+CH_3CN]^+$ 332.0834, found 332.0851.

NMR spectra

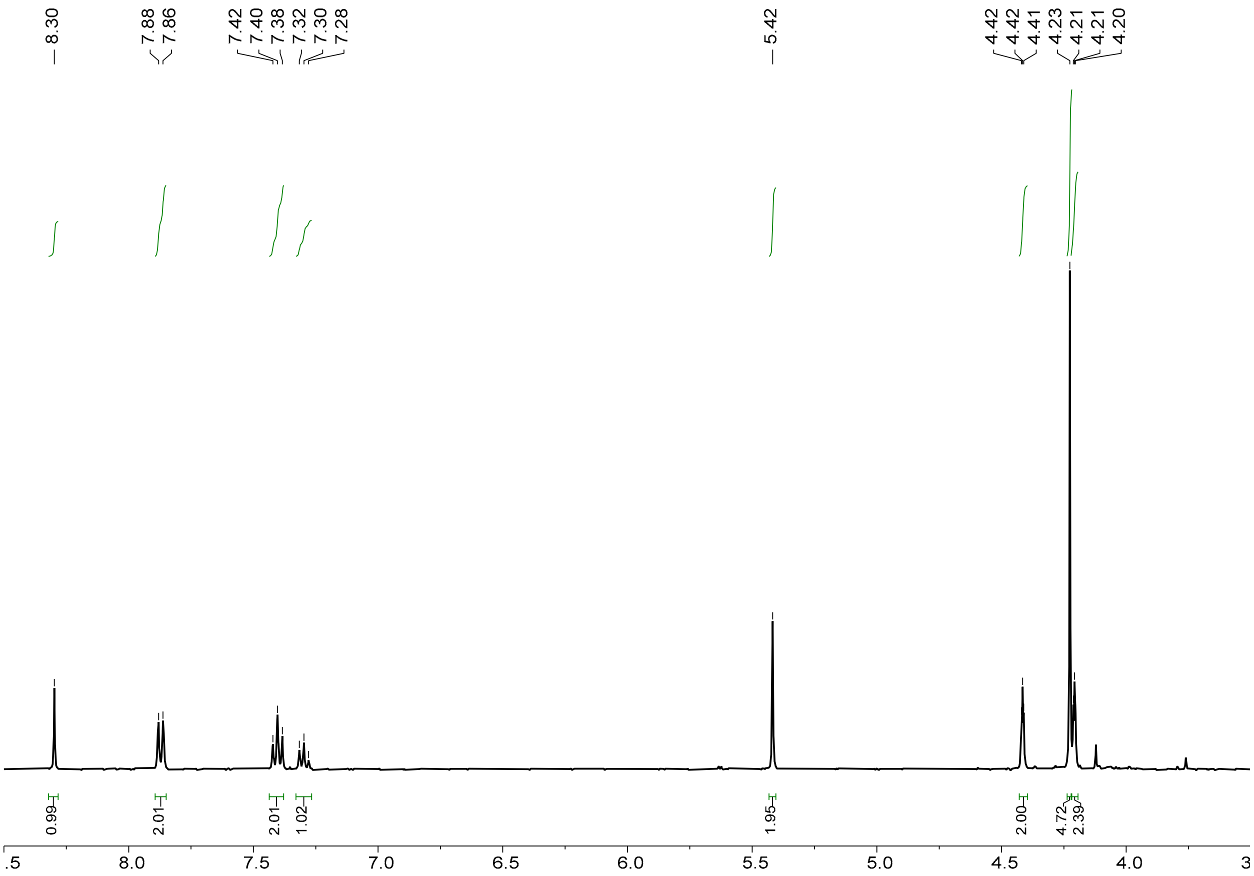

Figure S1.^{1}H NMR spectrum of **1** in ac-d6.

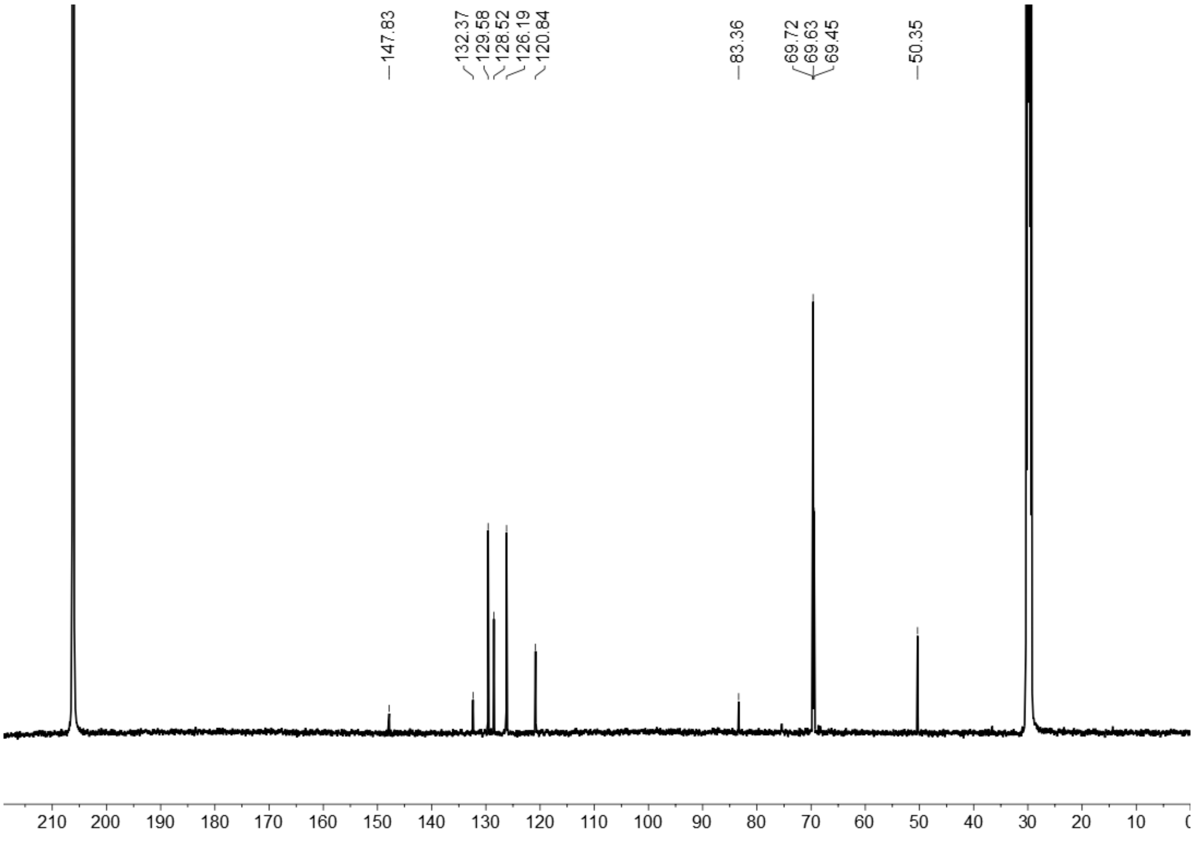

Figure S2.^{13}C NMR spectrum of **1** in ac-d6.

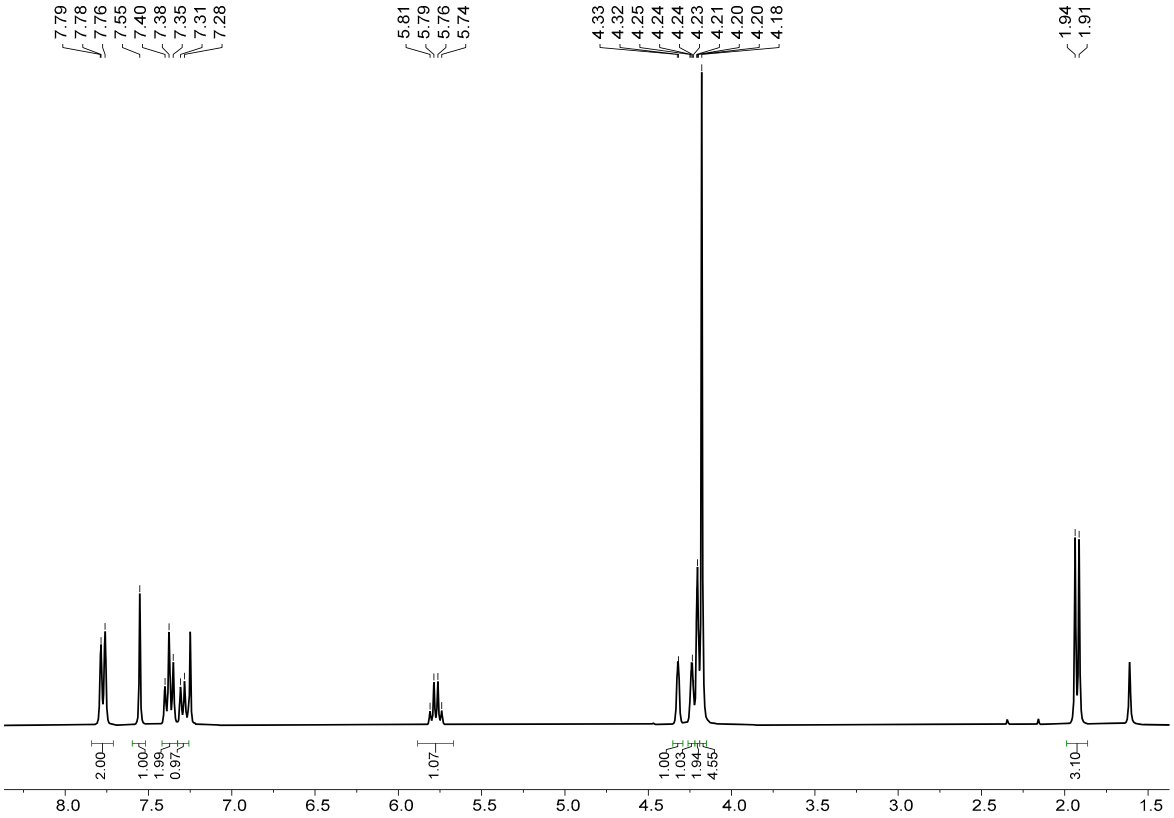

Figure S3. ^{1}H NMR spectrum of **2** in $CDCl_3$.

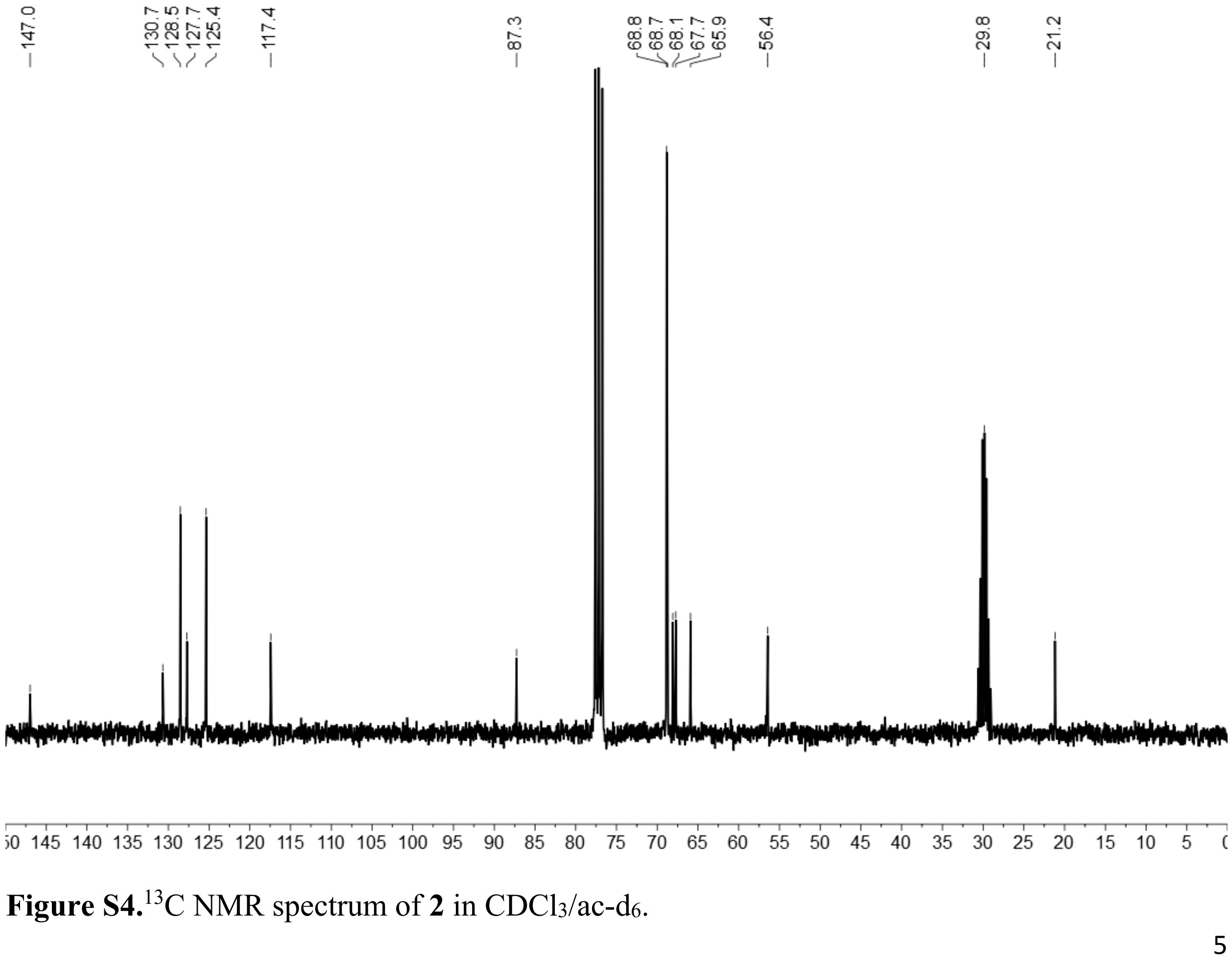

Figure S4. ^{13}C NMR spectrum of **2** in $CDCl_3$/ac-d_6.

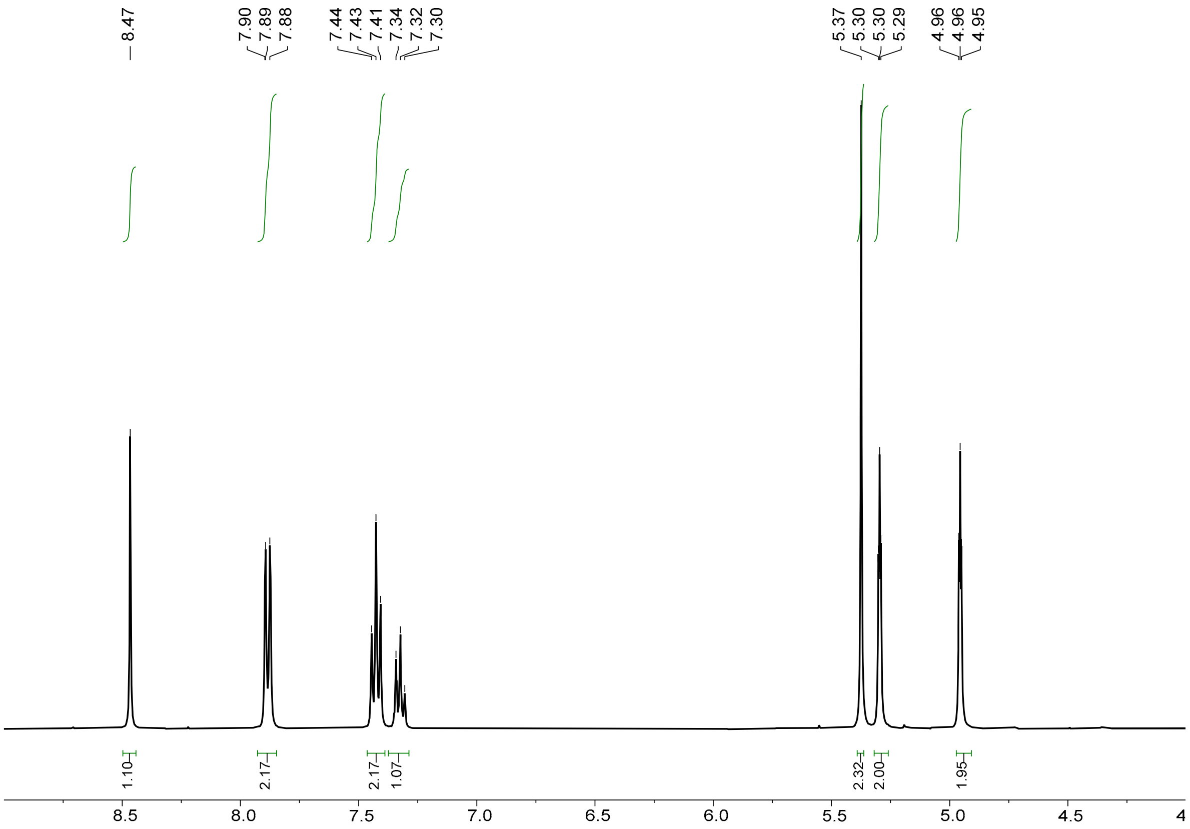

Figure S5. ^{1}H NMR spectrum of **3** in ac-d_6.

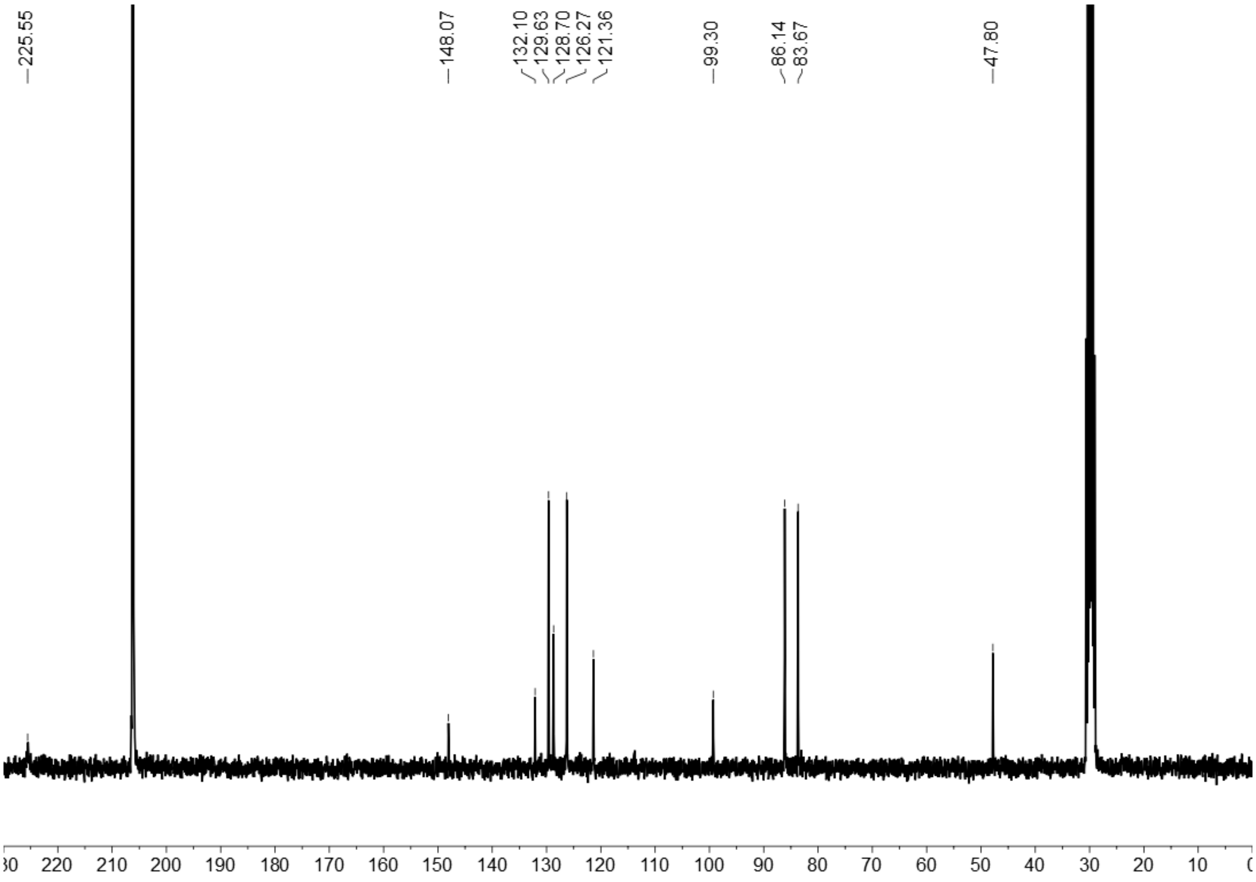

Figure S6. ^{13}C NMR spectrum of **3** in ac-d_6.

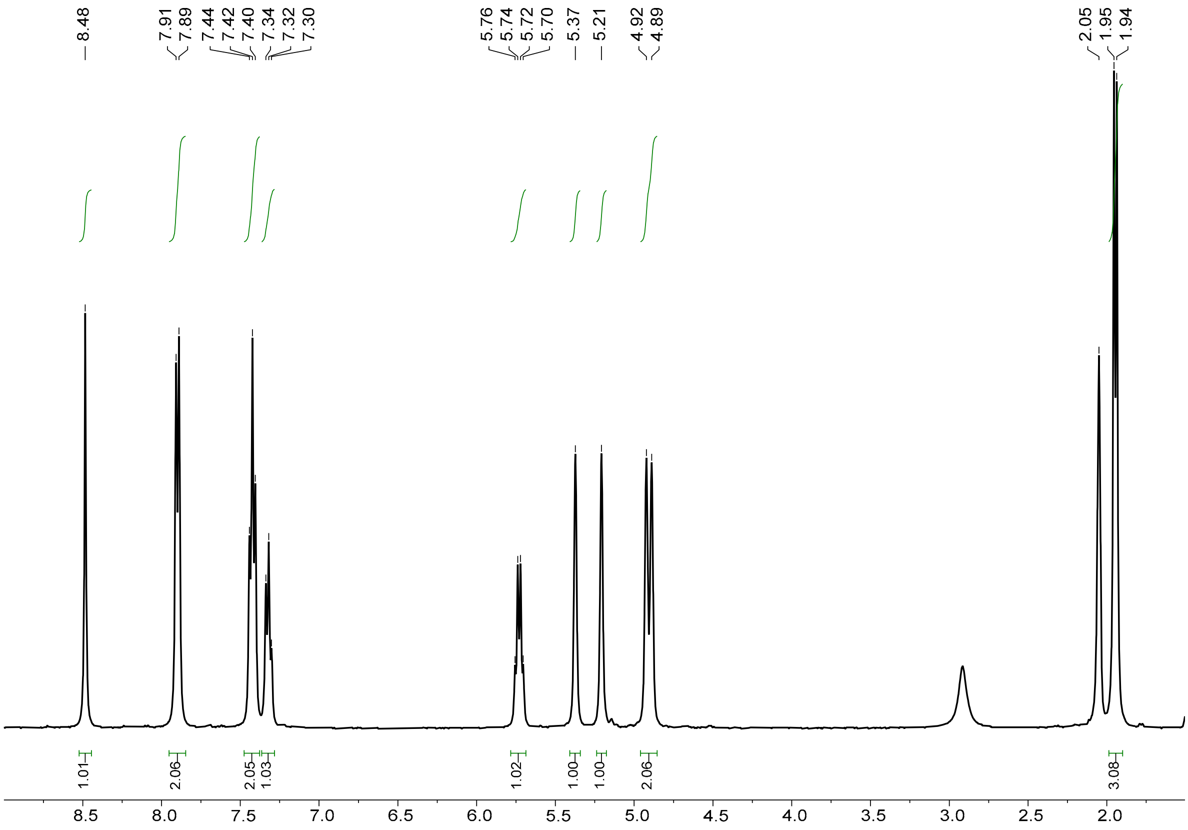

Figure S7. ^{1}H NMR spectrum of **4** in ac-d_6.

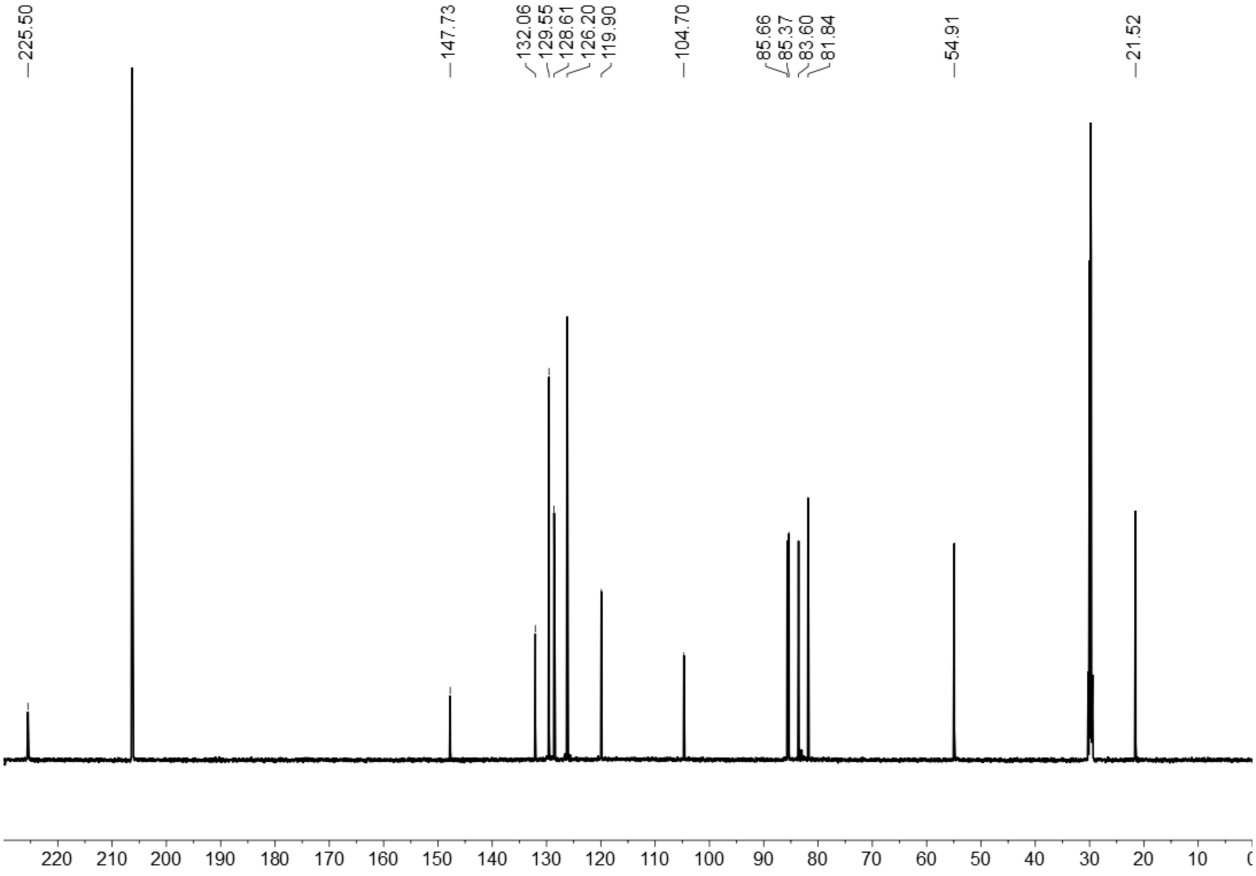

Figure S8. ^{13}C NMR spectrum of **4** in ac-d_6.

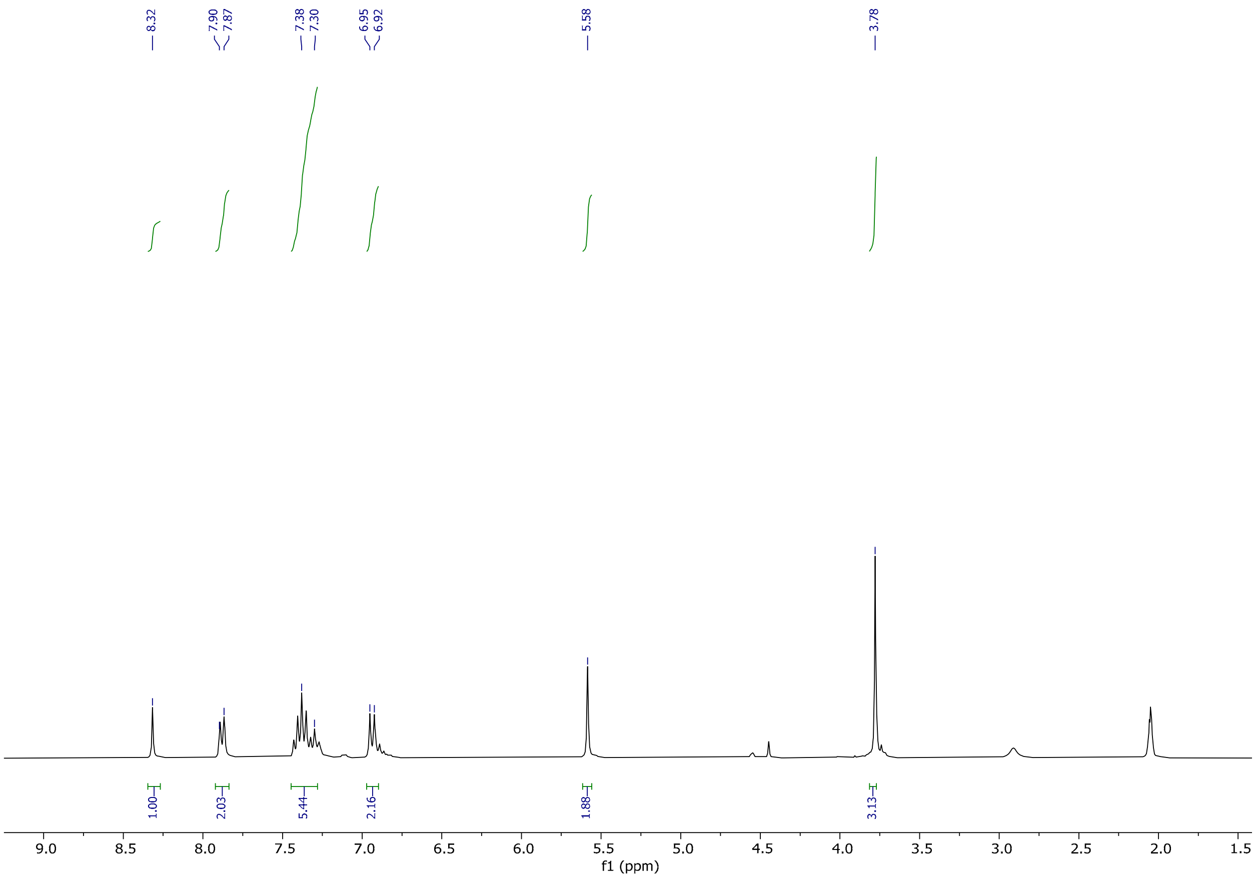

Figure S9.^{1}H NMR spectrum of **5** in ac-d6.

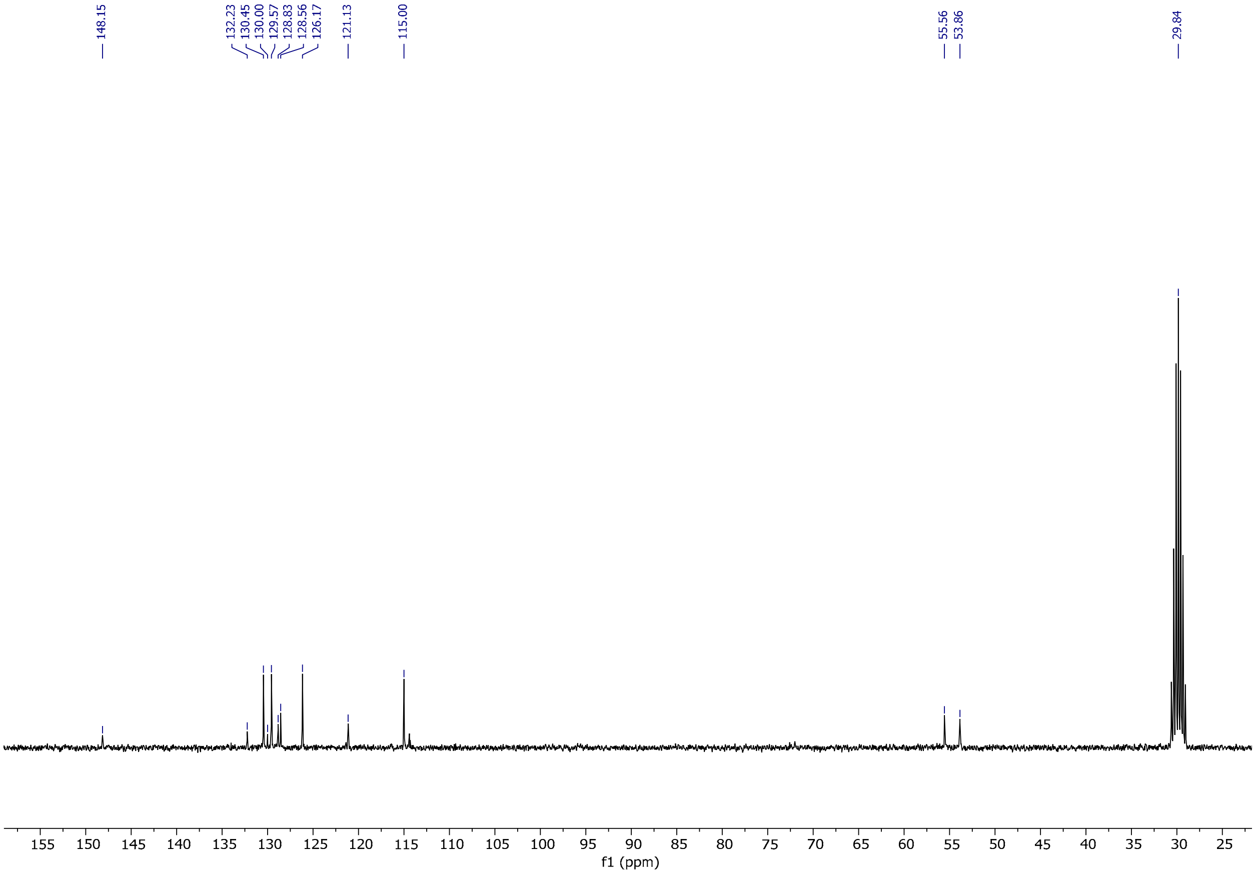

Figure S10.^{13}C NMR spectrum of **5** in ac-d6.

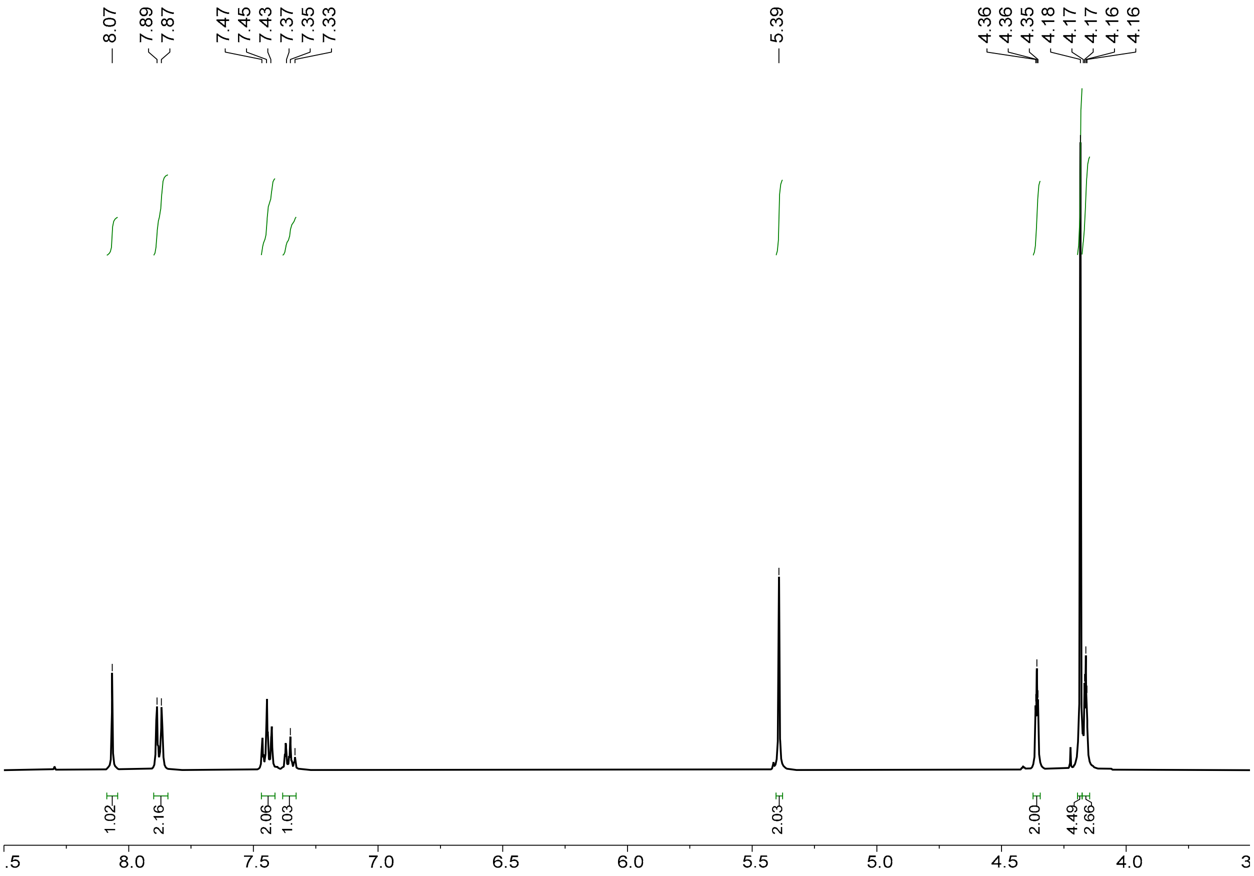

Figure S11.^{1}H NMR spectrum of **6** in ac-d6.

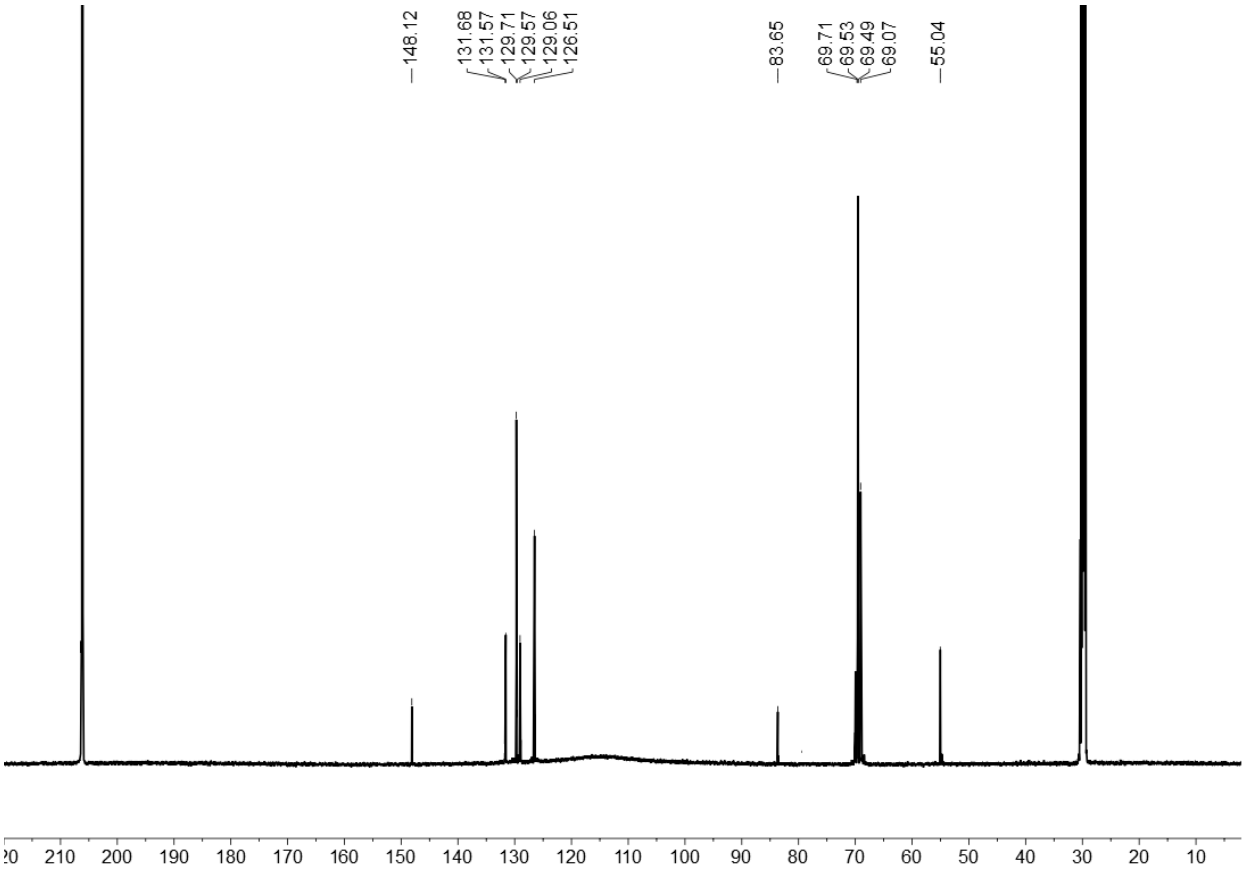

Figure S12.^{13}C NMR spectrum of **6** in ac-d6.

<u>Mass spectra</u>

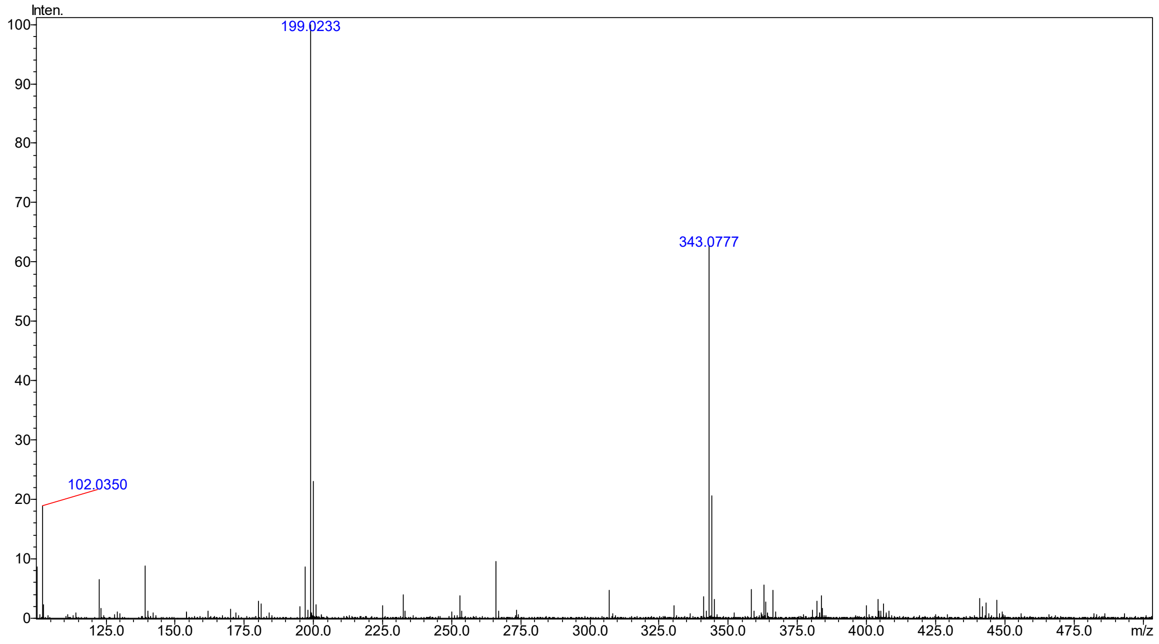

Figure S13. ESI-MS spectra (positive ions) of **1**.

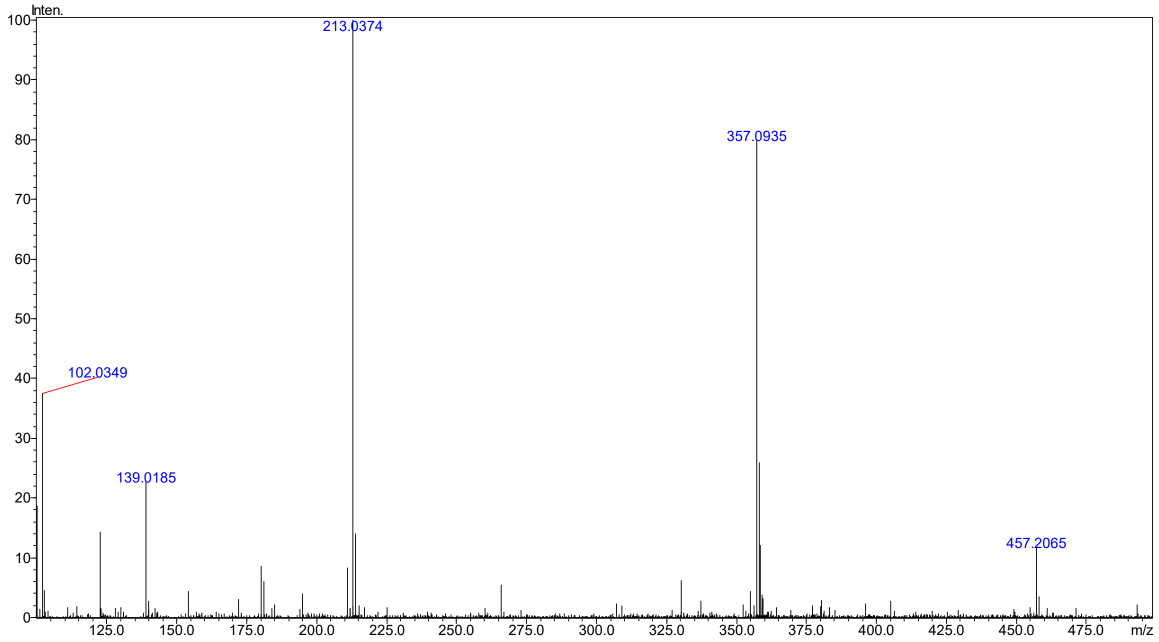

Figure S14. ESI-MS spectra (positive ions) of **2**.

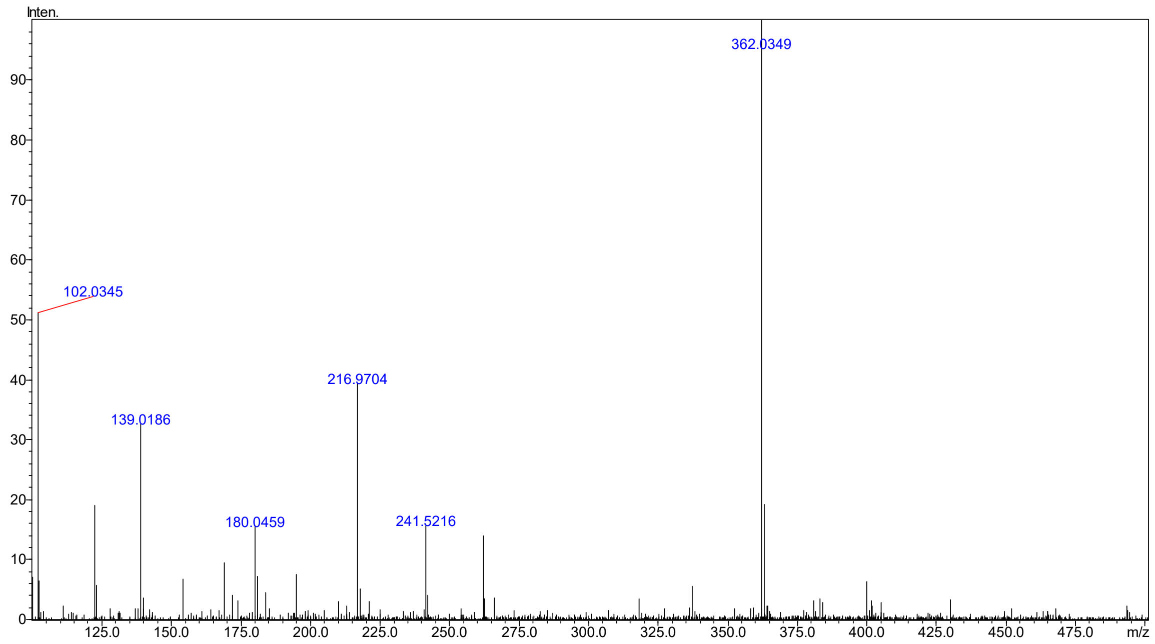

Figure S15. ESI-MS spectra (positive ions) of **3**.

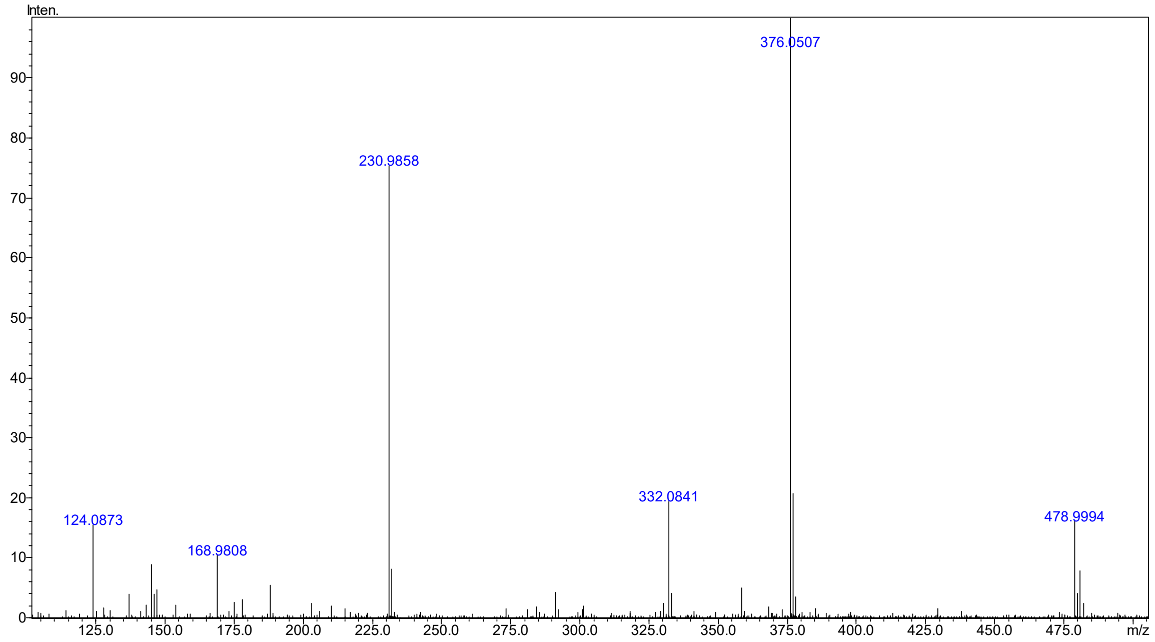

Figure S16. ESI-MS spectra (positive ions) of **4**.

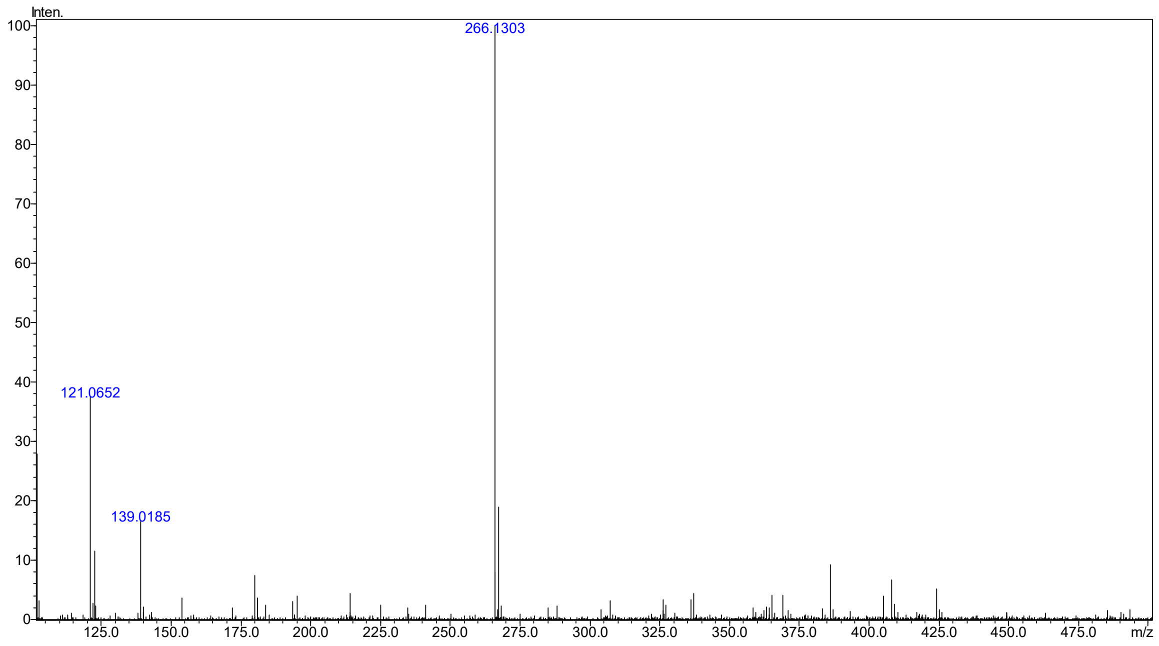

Figure S17. ESI-MS spectra (positive ions) of **5**.

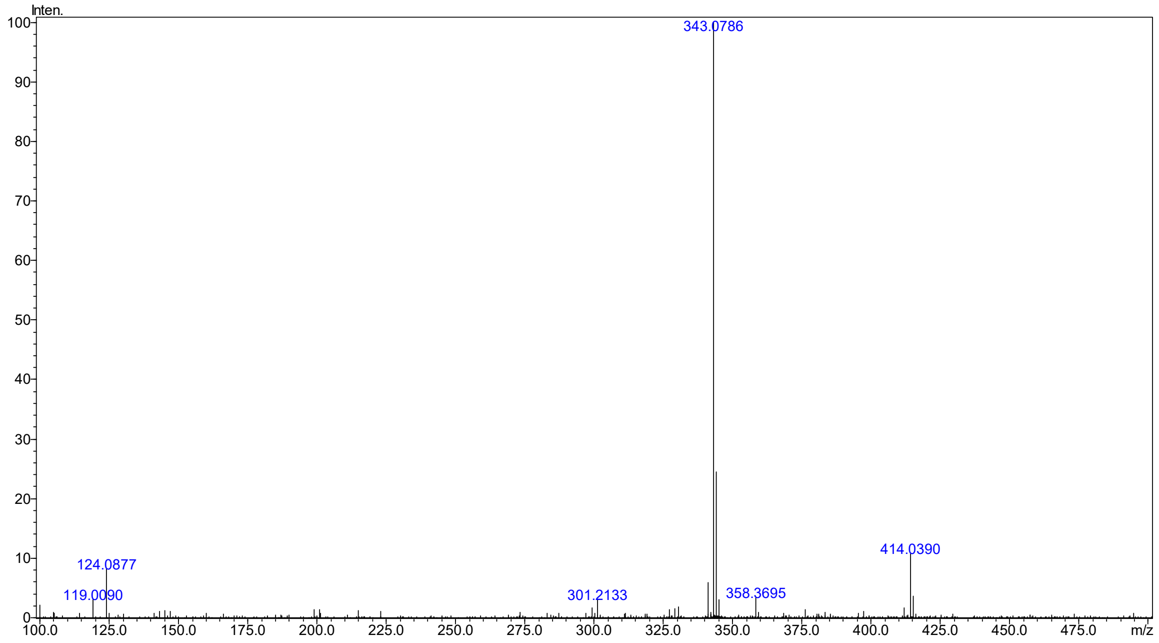

Figure S18. ESI-MS spectra (positive ions) of **6**.

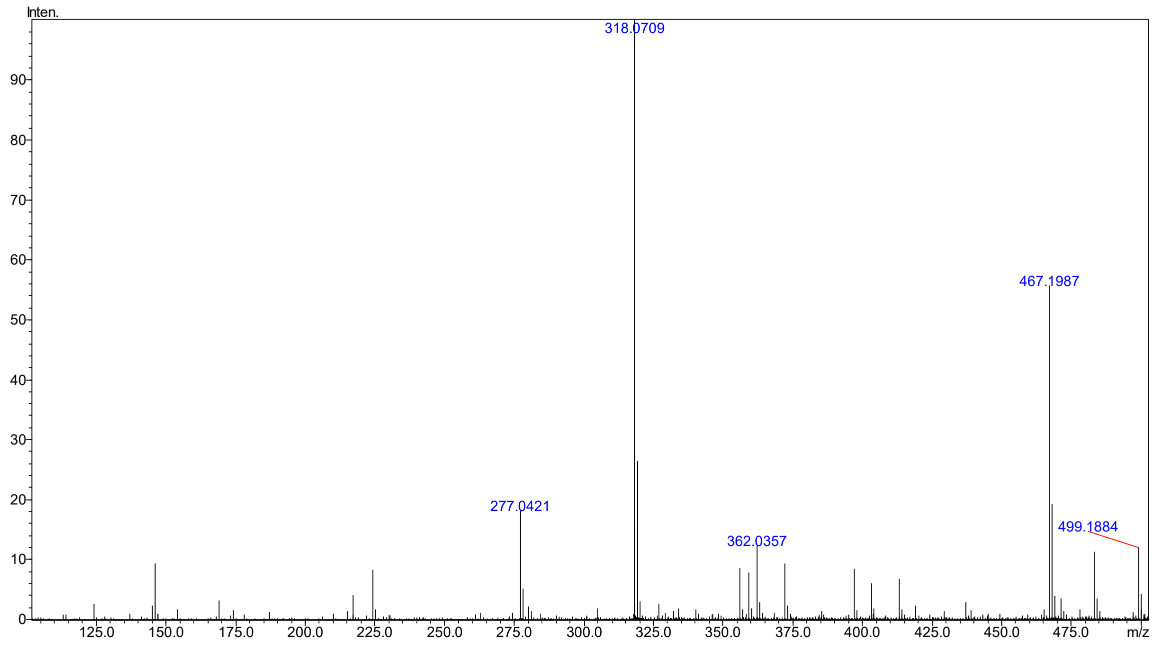

Figure S19. ESI-MS spectra (positive ions) of **3** after UV irradiation for 120 s.

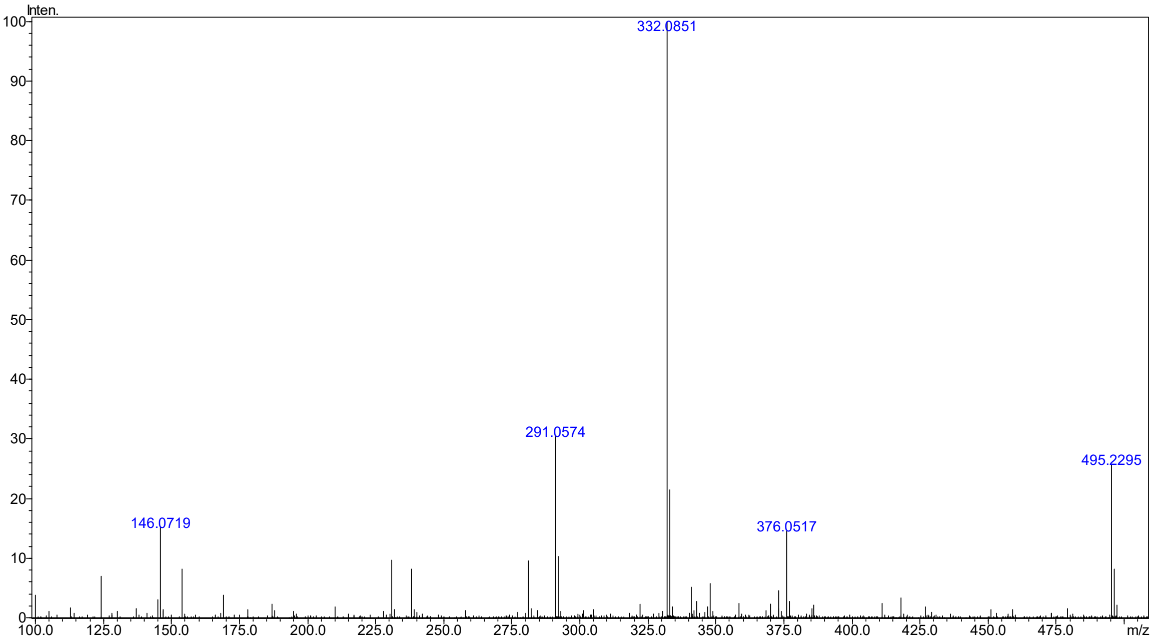

Figure S20. ESI-MS spectra (positive ions) of **4** after UV irradiation for 120 s.

Additional XRD Data

Table S1. The contribution (%) of different types of intermolecular interactions formed by the cation of **1**(**UCEDUE**) [45], **2**, **3** (**LAZDAU**) [46] and **6** (**UCEDOY**) [45] as obtained from the analysis of the Hirshfeld surfaces.

Interactions	**1**	**2**	**3**	**6**
H…H	52.5	58.0	25.3	60.5
O…H	-	-	12.5	-

C…H	17.7	15.2	11.5	12.7
N…H	8.5	7.7	7.7	5.5
H…C	13.9	11.4	8.9	9.6
H…N	7.4	6.1	6.9	4.6
C…C	-	1.3	2.1	2.5
C…N	-	0.1	0.4	2.3
N…C	-	0.1	0.3	2.2
N…O	-	-	0.1	-
O…O	-	-	5.0	-
O…N	-	-	0.1	-
O…C	-	-	4.2	-
C…O	-	-	5.0	-
H…O	-	-	10.0	-

Table S2. Crystallographic data and refinement parameters for **2**.

Parameter	**2**
Empirical formula	$C_{20}H_{19}FeN_3$
Formula weight	357.23
T (K)	120
Crystal system	Orthorhombic
Space group	$P2_12_12_1$
Z	4
a (Å)	35.0798(18)
b (Å)	8.3724(4)
c (Å)	24.3945(13)
a (°)	90
b (°)	96.019(2)

γ (°)	90
V (Å^3)	7125.2(6)
D_{calc} (g·cm^{-3})	1.332
Linear absorption, μ (cm^{-1})	5.3
F(000)	2976
$2\Theta_{max}$ (°)	52
Reflections measured	20816
Independent reflections	9377
Observed reflections [I>2σ(I)]	5623
Parameters	466
R1	0.0484
wR2	0.1343
GOF	1.036
$\Delta\rho_{max}$/ $\Delta\rho_{min}$ (e Å^{-3})	0.441/-0.844

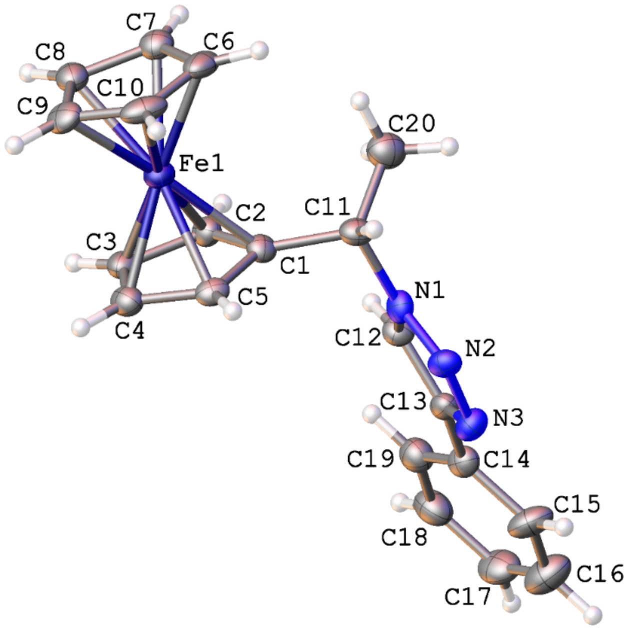

Figure S21. General view of the complex 2 from 2. Non-hydrogen atoms are shown as thermal ellipsoids (p = 50%).

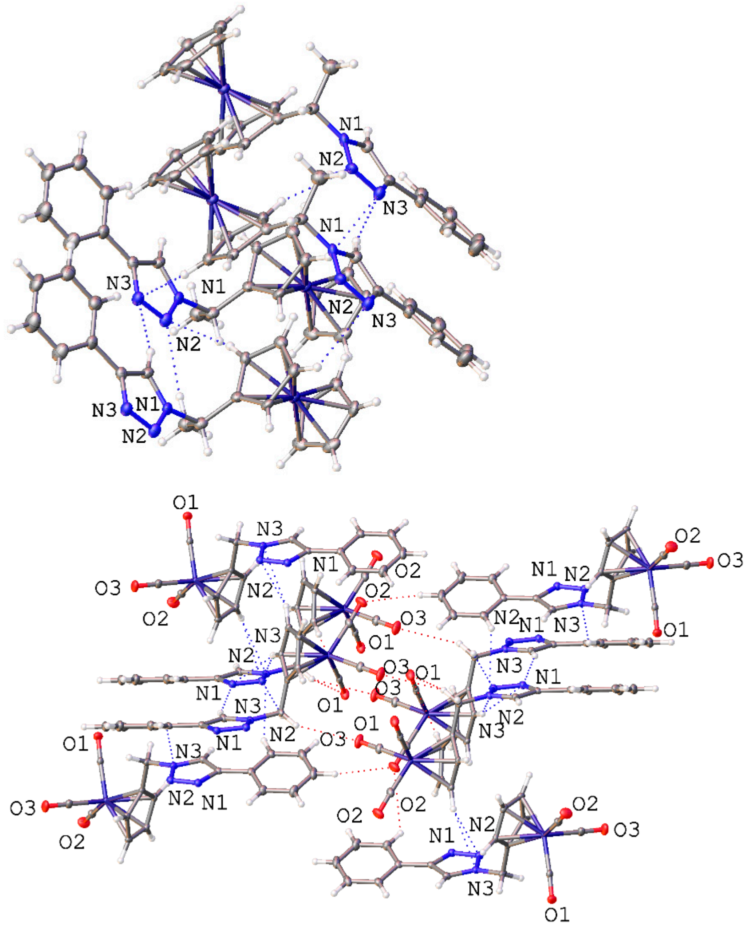

Figure S22. The fragment of crystal packing of compound **2** (top), **3**(**LAZDAU**) [2] (bottom). Hydrogen bonds are shown by dashed lines, only N and O atoms are named.

Additional Computational Data

Table S3. Selected bond lengths (Å) obtained from XRD and optimized structures (TPSSh-D3BJ/def2-TZVPP).

System	Bond	XRD value	Calculated value	Deviation
**3**	Mn–Cp	1.766	1.759	–0.007
**3**	Mn–CO1	1.797	1.793	–0.004
**3**	Mn–CO2	1.801	1.797	–0.004
**3**	Mn–CO3	1.802	1.796	–0.006
**di3**	Mn–Cp	1.765	1.740	–0.025
**di3**	Mn–CO1	1.775	1.788	0.013
**di3**	Mn–CO2	1.777	1.788	0.011
**di3**	Mn–N	1.983	1.965	–0.018

Cp stands for the centroid ofthe Cp ring carbons. Mean absolute deviations are 0.005 Å and 0.017 Å for **3** and **di3**, respectively.

Table S4. Energies of the highest occupied (HOMO) and lowest unoccupied (LUMO) molecular orbitals and HOMO-LUMO gaps (ΔE) in eV (TPSS/def2-TZVPP).

System	E_{HOMO}	E_{LUMO}	ΔE
**1**	–4.7368	–1.5643	3.1725
**2**	–4.7284	–1.5637	3.1647
**3**	–5.6728	–1.9574	3.7154
**4**	–5.6463	–1.9748	3.6715
**5**	–5.4271	–1.4119	4.0152
**6**	–4.5052	–1.5898	2.9154

Table S5. Electrostatic potential minima corresponding to the given triazolenitrogens, in a.u. (TPSS/def2-TZVPP).

Atom	**1**	**2**	**3**	**4**	**5**	**6**
N_{α}	–0.06374	–0.06439	–0.05518	–0.05651	–0.06301	–0.03576
N_{β}	–0.05795	–0.05827	–0.04834	–0.05060	–0.05771	–0.04590

The analysis was performed on the 0.001 a.u. isosurface of electron density of a molecule. The nitrogen designations are as follows:

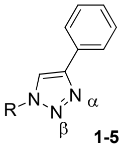

1-5

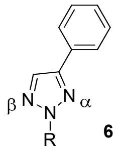

6

Computed Absorption Spectra

Table S6. Wavelength of the MLCT absorption of **di3** predicted by different methods (def2-TZVPP, benzene).

Method	MLCT wavelength, nm
Experimental value	467
TPSS	406
M06-L	395
r^{2}SCAN	392
DSD-PBEP86	392
SCAN	391
r^{2}SCAN-3c	390
M06	390
TPSSh	374
BHANDHLYP	369
CAM-B3LYP	364
B2BLYP	347
B3LYP	343
PBE0	329
mPW2PLYP	324
TPSS0	321
PBE0-DH	312
PBE-QIDH	304

Table S7. Absorption spectrum of **di3** (TPSS/def2-TZVPP, ethanol).

State	Energy, cm^{-1}	Wavelength, nm	Oscillatorstrength
1	23421.1	427.0	0.092445869
2	24306.4	411.4	0.001088163
3	25421.5	393.4	0.197981524
4	26098.6	383.2	0.000358842
5	26926.4	371.4	0.000714768
6	27029.6	370.0	0.000074252
7	28214.4	354.4	0.029484252
8	28961.0	345.3	0.000043185
9	29510.9	338.9	0.077094686
10	29643.7	337.3	0.000952184
11	31416.6	318.3	0.001145748
12	31717.0	315.3	0.000565605
13	31767.7	314.8	0.002459680
14	32073.4	311.8	0.000811320
15	34088.0	293.4	0.000219079
16	34352.0	291.1	0.001929127
17	35140.4	284.6	0.001662182
18	35726.2	279.9	0.001892277
19	35970.9	278.0	0.000249346
20	36075.0	277.2	0.000926966
21	36354.4	275.1	0.008212263
22	37195.7	268.8	0.006773443
23	37913.8	263.8	0.010332987
24	38161.7	262.0	0.003867582
25	38497.1	259.8	0.000387720
26	39405.9	253.8	0.035977195
27	39565.9	252.7	0.005171721
28	39691.0	251.9	0.011221912
29	39979.0	250.1	0.000265117
30	40220.9	248.6	0.001942372
31	40461.8	247.1	0.027317465
32	40525.1	246.8	0.118579482
33	40852.0	244.8	0.007303540
34	40884.6	244.6	0.313646149
35	41717.8	239.7	0.196217878
36	42338.9	236.2	0.001738306
37	42454.4	235.5	0.000784098
38	42576.9	234.9	0.000250650
39	42668.5	234.4	0.000002964
40	43107.7	232.0	0.010870284

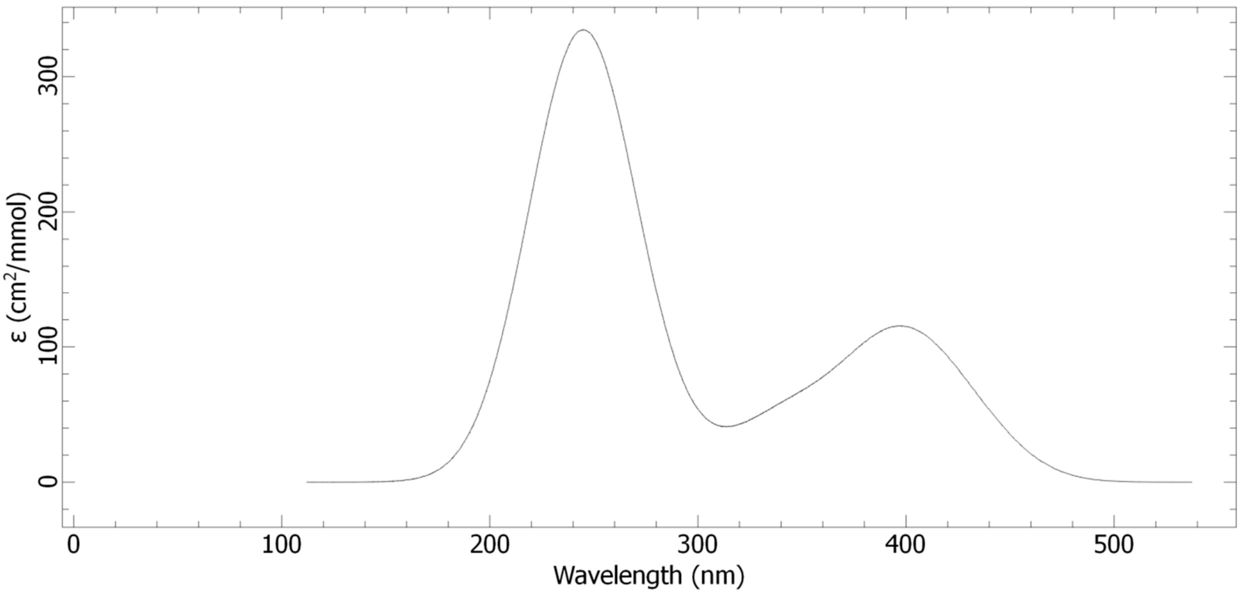

Figure S23. Visualized absorption spectrum of **di3** (TPSS/def2-TZVPP, ethanol). The spectral lines were convoluted with a Gaussian function with a full width at half maximum of 60 nm.

Table S8. Transitions between natural transition orbitals for state 3 of **di3** (TPSS/def2-TZVPP, ethanol).

Transition	Coefficient
84 => 85	0.96833888
83 => 86	0.01901099
82 => 87	0.00351221
81 => 88	0.00276745
80 => 89	0.00134555
79 => 90	0.00132612

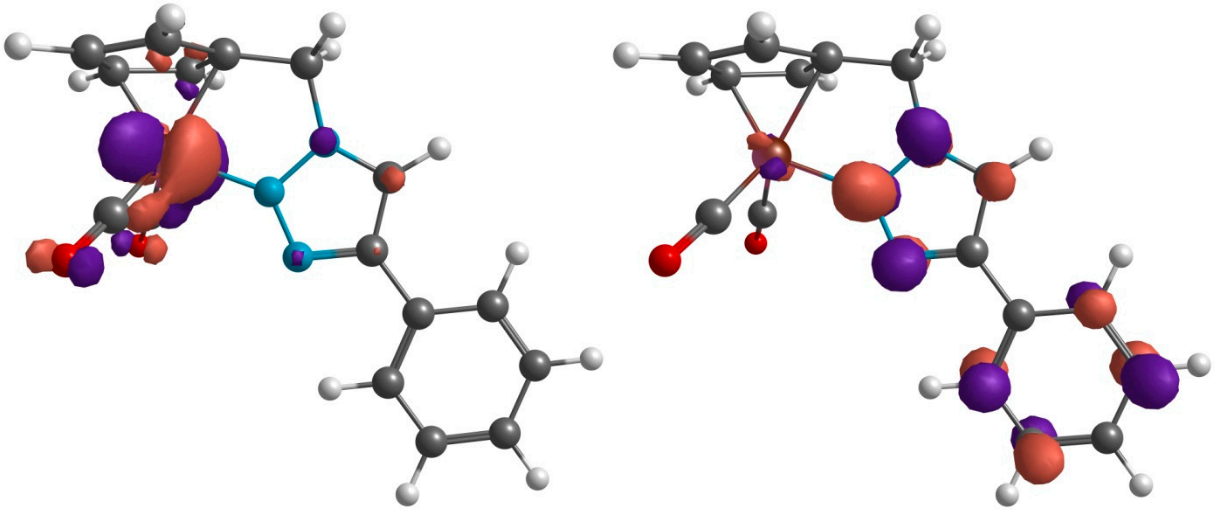

Figure S24. Depiction of the dominant natural transition orbitals pair for MLCT state (state 3) of **di3** (ρ = 0.065 a.u., TPSS/def2-TZVPP, ethanol).

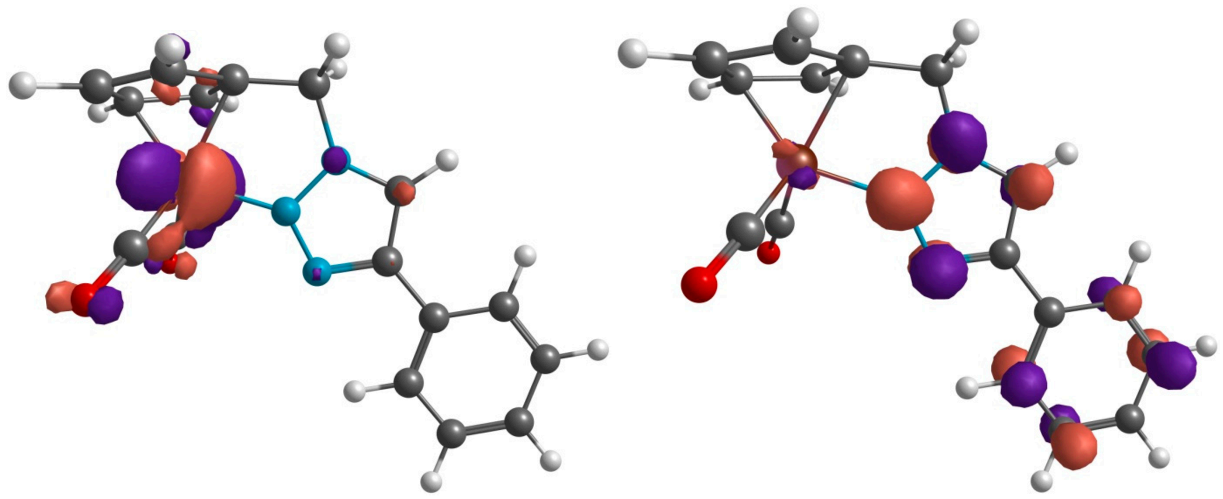

Figure S25. Depiction of the dominant natural transition orbitals pair for MLCT state (state 3) of **di3** (ρ = 0.065 a.u., M06-L/def2-TZVPP, ethanol).

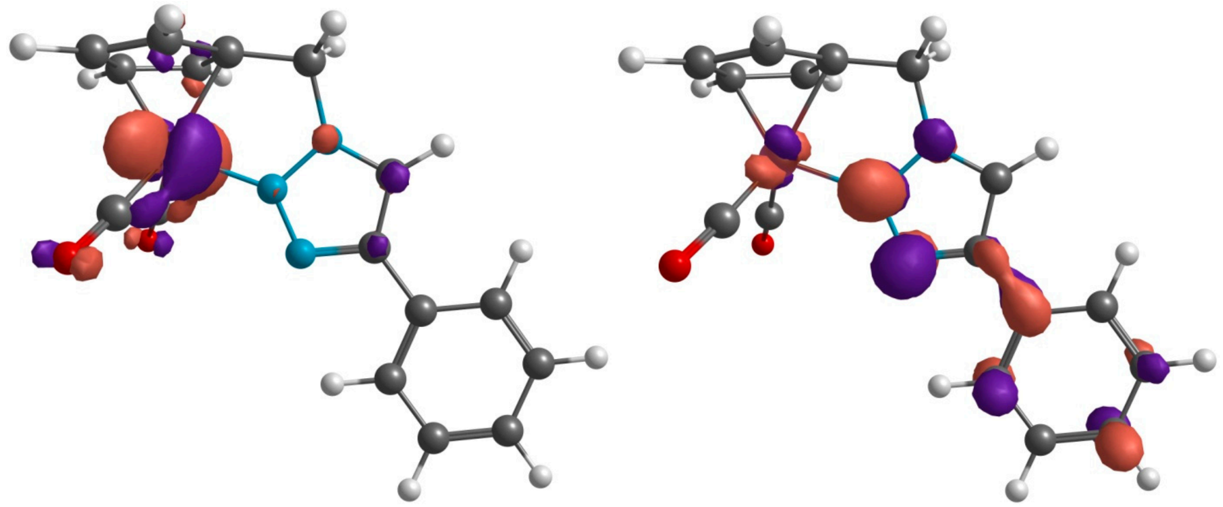

Figure S26. Depiction of the dominant natural transition orbitals pair for MLCT state (state 4) of **di3** (ρ = 0.065 a.u., B3LYP/def2-TZVPP, ethanol).

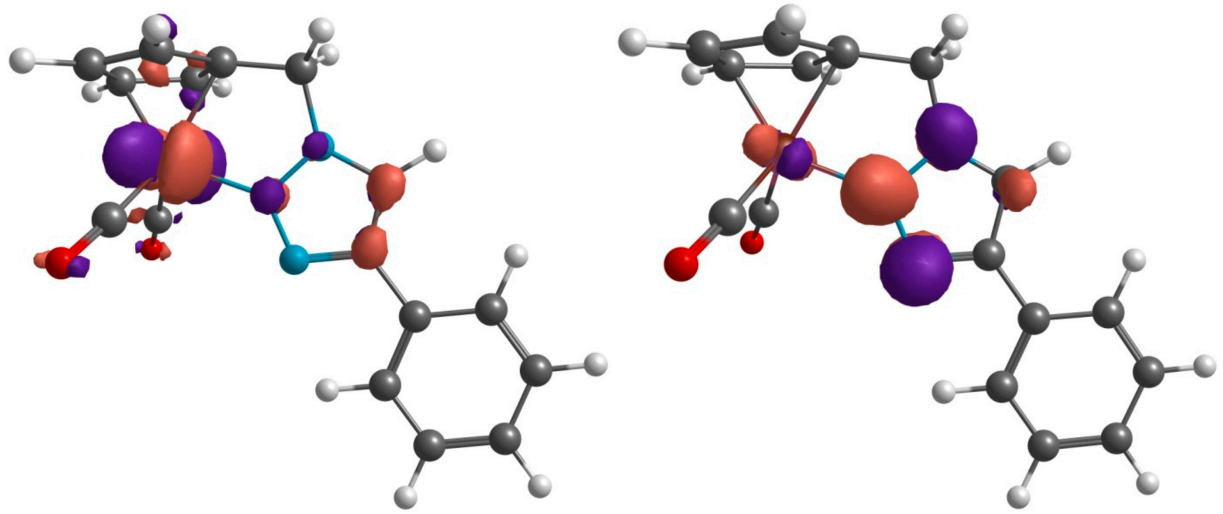

Figure S27. Depiction of the dominant natural transition orbitals pair for MLCT state (state 10) of **di3** (ρ = 0.065 a.u., PBE0-DH/def2-TZVPP, ethanol).

Table S9. Absorption spectrum of **3** (TPSS/def2-TZVPP, ethanol).

State	Energy, cm^{-1}	Wavelength, nm	Oscillatorstrength
1	31521.2	317.2	0.000365623
2	32180.5	310.7	0.023116256
3	32411.1	308.5	0.011898199
4	32542.7	307.3	0.016586149
5	32788.3	305.0	0.004422420
6	32859.1	304.3	0.006774196
7	33617.1	297.5	0.004137892
8	33618.5	297.5	0.002066945
9	34372.1	290.9	0.001218238
10	34435.6	290.4	0.008670133
11	35128.3	284.7	0.006580577
12	35546.5	281.3	0.001282096
13	35849.3	278.9	0.000918438
14	36736.3	272.2	0.011106999
15	37127.2	269.3	0.000019861
16	37341.6	267.8	0.021686365
17	37457.1	267.0	0.001221450
18	37738.7	265.0	0.000487150
19	38213.2	261.7	0.000029029
20	38405.2	260.4	0.024917153
21	38564.4	259.3	0.025023736
22	38876.4	257.2	0.000119087
23	39210.6	255.0	0.690475302
24	39603.5	252.5	0.006002710
25	39706.2	251.8	0.000685094
26	39806.0	251.2	0.003173081
27	39856.1	250.9	0.005532870
28	40160.0	249.0	0.000597014
29	40271.5	248.3	0.003014684
30	40364.2	247.7	0.000997134
31	40440.2	247.3	0.001998156
32	40469.3	247.1	0.000702662
33	40627.8	246.1	0.001652604
34	40710.8	245.6	0.000650041
35	40934.6	244.3	0.007961753
36	41147.6	243.0	0.004018982
37	41303.9	242.1	0.000542790
38	41577.0	240.5	0.000515675
39	41665.1	240.0	0.002794774
40	41764.4	239.4	0.139367121

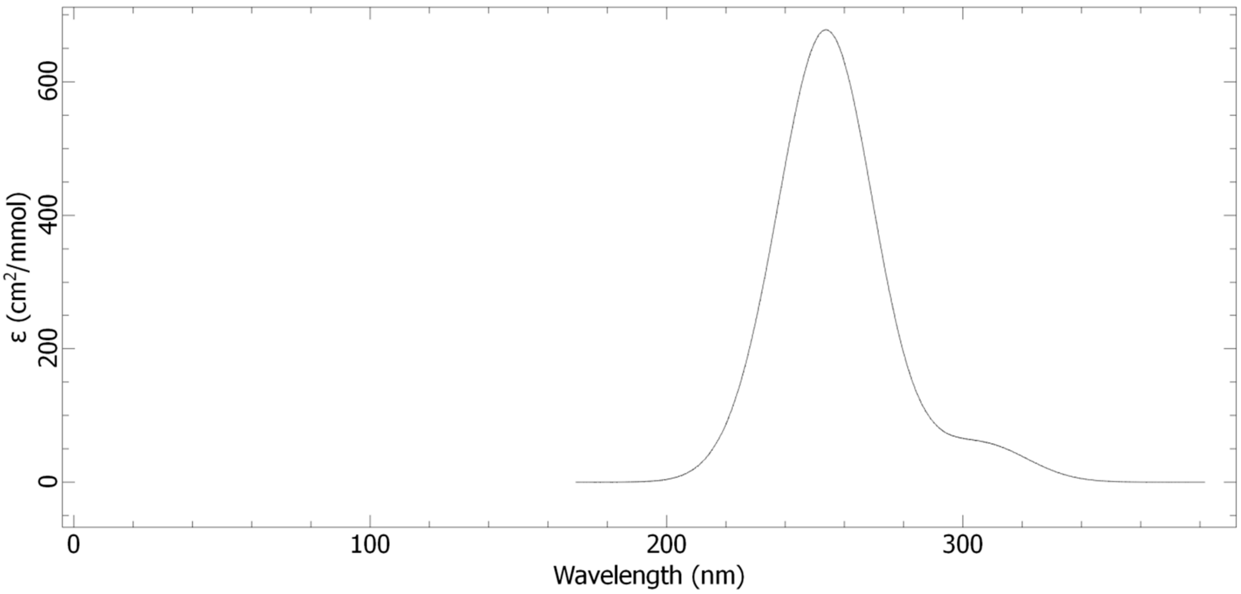

Figure S28. Visualized absorption spectrum of **3** (TPSS/def2-TZVPP, ethanol). The spectral lines were convoluted with a Gaussian function with a full width at half maximum of 35 nm.

Table S10. Transitions between natural transition orbitals for state 2 of **3** (TPSS/def2-TZVPP, ethanol).

Transition	Coefficient
91 => 92	0.81306819
90 => 93	0.16967807
89 => 94	0.01410727
88 => 95	0.00227313

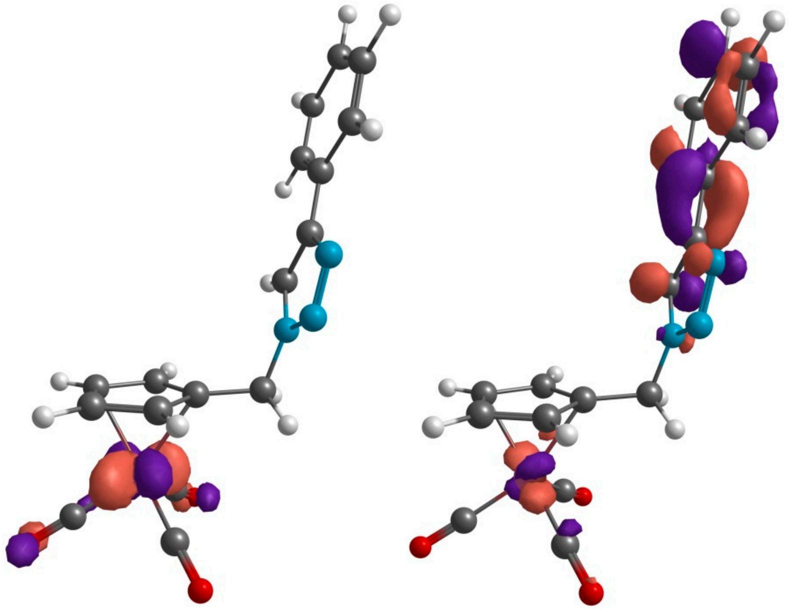

Figure S29. Depiction of 91–92 natural transition orbitals pair for state 2 of **3** (ρ = 0.08 a.u., TPSS/def2-TZVPP, ethanol).

Table S11. Transitions between natural transition orbitals for state 4 of **3** (TPSS/def2-TZVPP, ethanol).

Transition	Coefficient
91 => 92	0.63405980
90 => 93	0.31836263
89 => 94	0.03392527
88 => 95	0.01203118

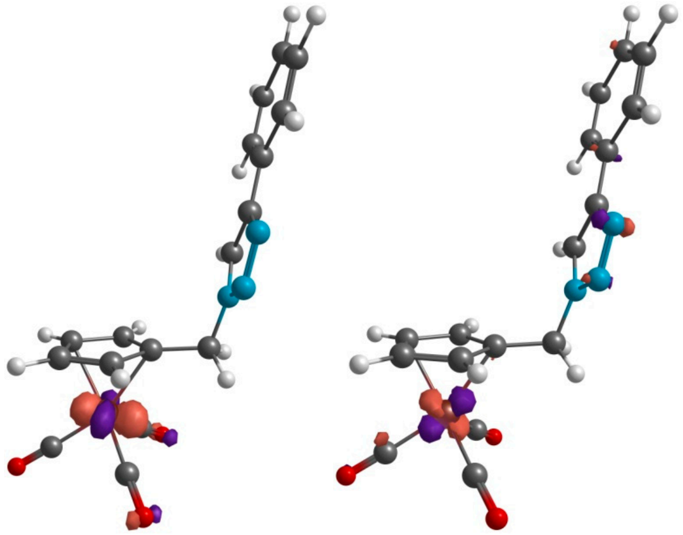

Figure S30. Depiction of 91–92 natural transition orbitals pair for state 4 of **3** (ρ = 0.1 a.u., TPSS/def2-TZVPP, ethanol).

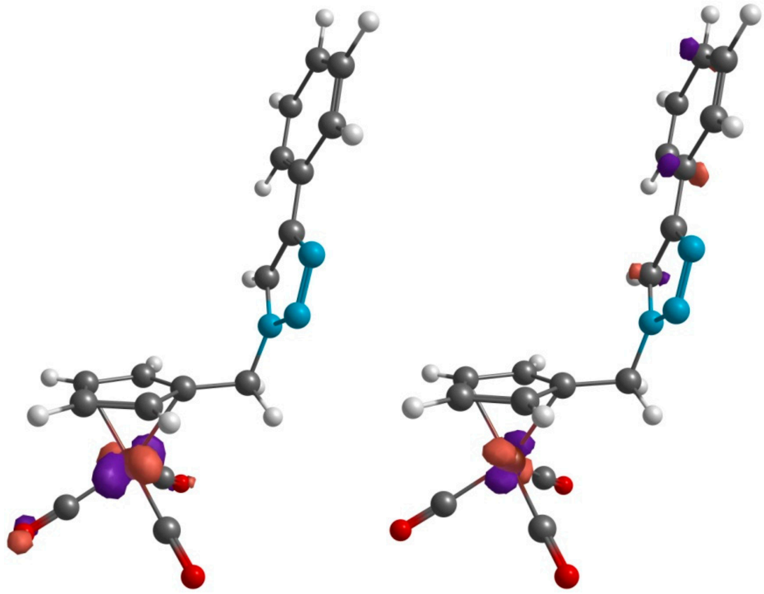

Figure S31. Depiction of 90–93 natural transition orbitals pair for state 4 of **3** (ρ = 0.1 a.u., TPSS/def2-TZVPP, ethanol).

Molecular Electrostatic Potential Maps

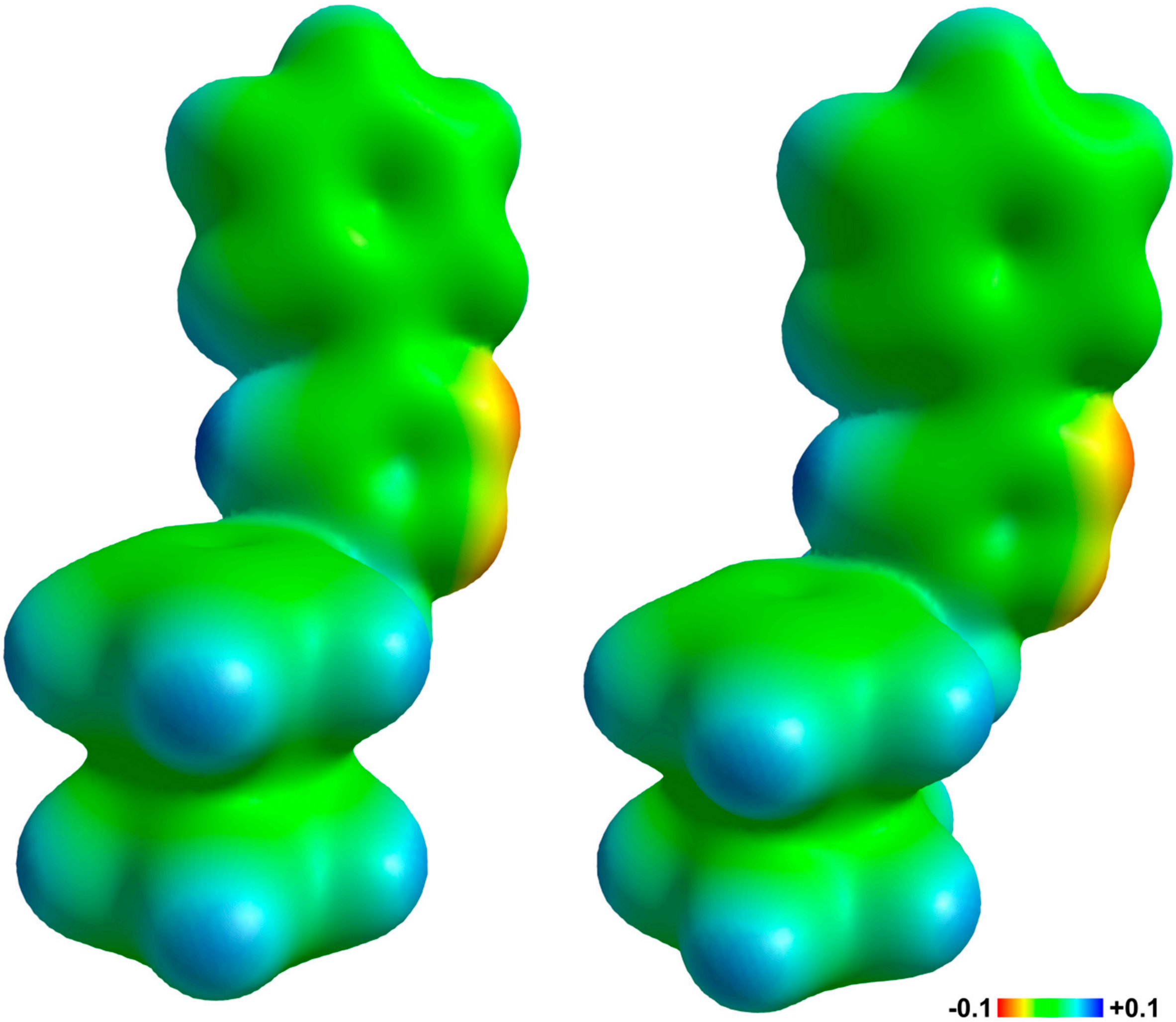

Figure S32. Molecular electrostatic potential maps of**1** (left) and **2** (right).

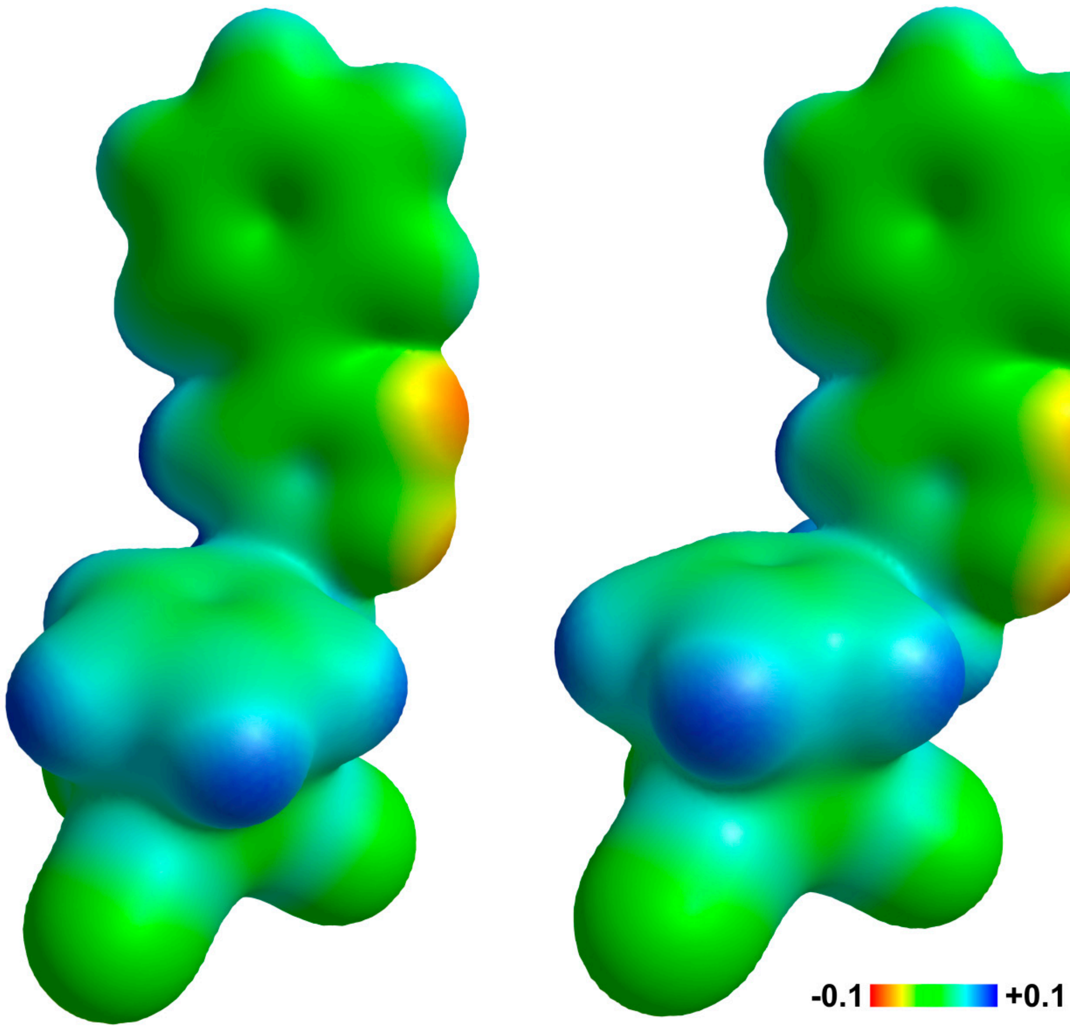

Figure S33. Molecular electrostatic potential maps of**3** (left) and **4** (right).

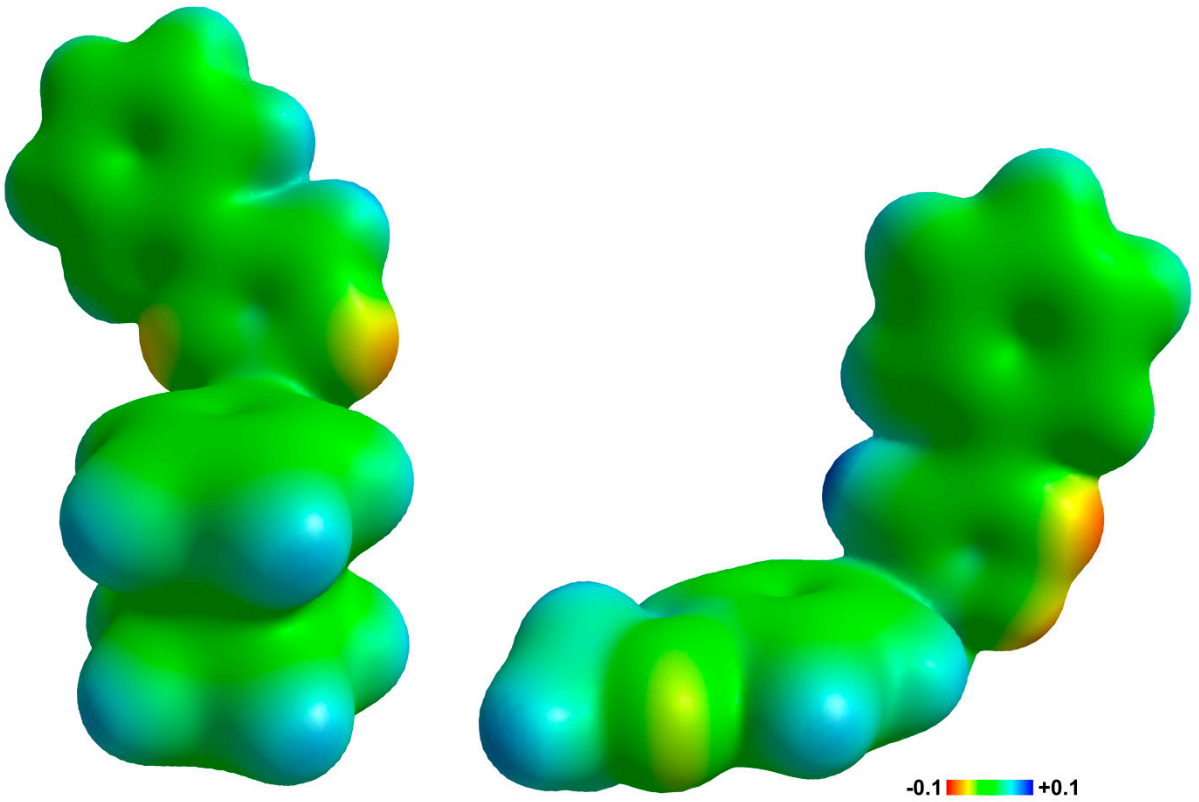

Figure S34. Molecular electrostatic potential maps of**6** (left) and **5** (right).

Cartesian Coordinates of Optimized Structures

The data below are given in multiple XYZ-file format: the first line represents the number of atoms (N), the second line is a comment presenting the system, and the following N lines contain atomic Cartesian coordinates in angstroms. Multiple structures are placed directly end-to-end.

```
40
structure of 1
Fe      -1.377033000       0.092687000      -0.006411000
C       -2.857655000       1.417714000      -0.374324000
C       -3.188411000       0.606380000       0.756750000
C       -2.679259000       0.544948000      -1.495687000
C       -3.200828000      -0.754430000       0.337606000
H       -3.371530000       0.968621000       1.756292000
C       -2.884855000      -0.793791000      -1.053055000
H       -2.401533000       0.852120000      -2.492506000
H       -3.383913000      -1.609154000       0.969219000
H       -2.787836000      -1.682632000      -1.656023000
C        0.281532000      -1.060359000      -0.278533000
C       -0.032556000      -0.955167000       1.108947000
C        0.501244000       0.255772000      -0.781909000
H        0.318107000      -1.972592000      -0.852610000
C       -0.006196000       0.426253000       1.463798000
H       -0.274756000      -1.773894000       1.767775000
C        0.323505000       1.174382000       0.295015000
H        0.735466000       0.513303000      -1.802707000
H       -0.223508000       0.835013000       2.437869000
H        0.405111000       2.248353000       0.232237000
C       -2.749972000       2.906666000      -0.389864000
```

H	-2.315496000	3.292789000	0.530405000
H	-2.132332000	3.237058000	-1.227671000
N	-4.065061000	3.544625000	-0.490652000
C	-5.124757000	3.199849000	-1.253573000
C	-6.080690000	4.158964000	-0.977330000
H	-5.112078000	2.329131000	-1.884210000
C	-7.440175000	4.315410000	-1.490449000
C	-8.222587000	5.401205000	-1.079891000
C	-7.986824000	3.395203000	-2.392373000
C	-9.515509000	5.558697000	-1.561172000
H	-7.799895000	6.111816000	-0.381817000
C	-9.280093000	3.555862000	-2.872121000
H	-7.398760000	2.545677000	-2.719975000
C	-10.051081000	4.638883000	-2.458879000
H	-10.108760000	6.404314000	-1.233960000
H	-9.687499000	2.833432000	-3.569343000
H	-11.059966000	4.763984000	-2.832885000
N	-4.330839000	4.653553000	0.226518000
N	-5.542388000	5.024893000	-0.065277000

43

structure of **2**

Fe	-1.406421000	0.101239000	-0.010119000
C	-2.811164000	1.473412000	-0.497739000
C	-3.238789000	0.698201000	0.626995000
C	-2.597752000	0.568990000	-1.587453000
C	-3.277839000	-0.670305000	0.236548000
H	-3.470211000	1.090123000	1.605212000
C	-2.879588000	-0.751072000	-1.130383000
H	-2.248035000	0.834122000	-2.572391000
H	-3.530409000	-1.503432000	0.873106000
H	-2.777957000	-1.655416000	-1.709207000
C	0.258932000	-1.043806000	-0.268849000
C	-0.157219000	-1.068672000	1.095136000
C	0.525313000	0.311610000	-0.623863000
H	0.331931000	-1.896693000	-0.924885000
C	-0.147854000	0.271206000	1.583860000
H	-0.454824000	-1.943582000	1.651094000
C	0.274019000	1.123943000	0.521851000
H	0.838727000	0.662181000	-1.594634000
H	-0.435618000	0.587367000	2.573986000
H	0.368463000	2.197491000	0.573358000
C	-2.667353000	2.966105000	-0.514985000
H	-2.267946000	3.304312000	0.441651000
N	-4.001598000	3.588011000	-0.586278000
C	-5.104847000	3.173383000	-1.245607000
C	-6.036708000	4.171065000	-1.028471000
H	-5.133663000	2.235751000	-1.770936000
C	-7.422528000	4.285229000	-1.478517000
C	-8.176092000	5.416939000	-1.145036000
C	-8.023102000	3.279228000	-2.244336000
C	-9.493290000	5.535276000	-1.568259000
H	-7.711722000	6.194155000	-0.552319000
C	-9.340623000	3.400775000	-2.666235000
H	-7.457720000	2.393723000	-2.511041000
C	-10.082584000	4.529818000	-2.330265000
H	-10.063452000	6.417410000	-1.301934000
H	-9.789553000	2.611867000	-3.257990000
H	-11.110357000	4.624495000	-2.659148000
N	-4.216539000	4.776517000	0.008488000
N	-5.441181000	5.128787000	-0.254582000
C	-1.789727000	3.492996000	-1.648167000
H	-0.792352000	3.058963000	-1.575762000
H	-2.213855000	3.232783000	-2.619176000

H	-1.714979000	4.578044000	-1.584075000

36

structure of **3**

Mn	-2.425050000	-0.250531000	0.433682000
C	-1.262982000	1.283793000	1.367911000
C	-2.174153000	0.740854000	2.303160000
C	-2.020919000	1.849527000	0.295255000
H	-0.187968000	1.266265000	1.446046000
C	-3.503564000	0.949454000	1.819803000
H	-1.911074000	0.231384000	3.217478000
C	-3.391513000	1.648535000	0.580637000
H	-1.619275000	2.337541000	-0.577822000
H	-4.222038000	1.959208000	-0.033749000
C	-3.683805000	-0.793338000	-0.726311000
O	-4.511789000	-1.114097000	-1.458500000
C	-2.420445000	-1.836435000	1.270319000
O	-2.429096000	-2.833018000	1.848197000
C	-1.090214000	-0.730557000	-0.669228000
O	-0.217598000	-1.004709000	-1.367619000
C	-4.781134000	0.601997000	2.520625000
H	-5.559284000	0.339886000	1.806704000
H	-4.638035000	-0.234136000	3.204497000
C	-4.948159000	2.185442000	4.506706000
N	-6.198735000	2.563360000	2.709462000
C	-5.684260000	3.341185000	4.681905000
H	-4.239776000	1.664064000	5.125920000
N	-5.302245000	1.735575000	3.281525000
N	-6.433034000	3.524862000	3.550501000
C	-5.744083000	4.266385000	5.811930000
C	-4.958844000	4.070444000	6.953684000
C	-6.598632000	5.373985000	5.763258000
C	-5.025806000	4.957668000	8.019827000
H	-4.289981000	3.219345000	7.010921000
C	-6.662721000	6.259514000	6.830864000
H	-7.205951000	5.524534000	4.880438000
C	-5.878359000	6.056754000	7.963218000
H	-4.411381000	4.791086000	8.896492000
H	-7.329102000	7.112242000	6.779251000
H	-5.930725000	6.748713000	8.794932000

34

structure of **di3**

Mn	2.459650000	6.830466000	6.524746000
O	3.662766000	6.448423000	3.866245000
O	1.948924000	9.692211000	6.066031000
N	4.144742000	7.049509000	7.512027000
N	5.209902000	7.768432000	7.317313000
N	4.308036000	6.337012000	8.653035000
C	6.090396000	7.533928000	8.335156000
C	7.388199000	8.202640000	8.394662000
C	2.002477000	4.790838000	6.912203000
H	2.620856000	3.955660000	6.624141000
C	5.515745000	6.616906000	9.193433000
H	5.848185000	6.157669000	10.107081000
C	2.187453000	8.574688000	6.243390000
C	3.218109000	6.622489000	4.919486000
C	0.949205000	5.378167000	6.145073000
H	0.626971000	5.058948000	5.167034000
C	1.100062000	6.542528000	8.132439000
H	0.920072000	7.254133000	8.922415000
C	0.398134000	6.448880000	6.891144000
H	-0.414695000	7.083672000	6.576875000
C	7.746433000	9.119446000	7.399739000
H	7.049386000	9.318401000	6.596397000

C	8.288567000	7.944221000	9.434132000
H	8.029177000	7.236393000	10.213065000
C	2.101219000	5.530161000	8.124000000
C	3.236709000	5.434335000	9.098847000
H	2.931219000	5.736787000	10.101438000
H	3.639563000	4.421532000	9.143827000
C	9.867843000	9.498135000	8.485771000
H	10.827344000	9.999432000	8.520954000
C	8.977358000	9.760180000	7.448227000
H	9.242499000	10.467754000	6.671999000
C	9.518226000	8.587088000	9.478919000
H	10.205006000	8.376593000	10.289843000

39

structure of **4**

Mn	-2.369370000	-0.187270000	0.494662000
C	-1.187963000	1.086502000	1.741439000
C	-2.213693000	0.474802000	2.511880000
C	-1.821377000	1.869915000	0.731456000
H	-0.126745000	0.979330000	1.895099000
C	-3.476994000	0.859506000	1.981944000
H	-2.056608000	-0.194340000	3.343027000
C	-3.220132000	1.734975000	0.876266000
H	-1.318836000	2.449479000	-0.026482000
H	-3.974086000	2.204961000	0.264680000
C	-3.806438000	-0.715613000	-0.438959000
O	-4.758607000	-1.027088000	-1.006806000
C	-2.006780000	-1.859313000	1.032290000
O	-1.777318000	-2.920025000	1.418184000
C	-1.275983000	-0.333681000	-0.924980000
O	-0.560874000	-0.397116000	-1.824072000
C	-4.842540000	0.520194000	2.515474000
H	-5.542229000	0.439069000	1.683570000
C	-4.879825000	2.240458000	4.403112000
N	-6.456624000	2.314408000	2.839796000
C	-5.743240000	3.289105000	4.655699000
H	-4.000397000	1.877535000	4.904444000
N	-5.359161000	1.665027000	3.277776000
N	-6.690300000	3.287654000	3.667929000
C	-5.745436000	4.275927000	5.733857000
C	-4.760112000	4.268435000	6.727638000
C	-6.746491000	5.253055000	5.784317000
C	-4.774208000	5.212603000	7.745805000
H	-3.975916000	3.520355000	6.706115000
C	-6.757615000	6.195701000	6.803749000
H	-7.507965000	5.257639000	5.015500000
C	-5.773446000	6.180987000	7.788546000
H	-4.003888000	5.192748000	8.507425000
H	-7.538871000	6.945932000	6.830146000
H	-5.784518000	6.917375000	8.582835000
C	-4.887743000	-0.755497000	3.349989000
H	-5.904875000	-0.933668000	3.697509000
H	-4.566309000	-1.602983000	2.744748000
H	-4.235211000	-0.686193000	4.222028000

35

structure of **5**

C	-2.658407000	3.037181000	-0.544196000
H	-2.150917000	3.611030000	0.231694000
H	-2.139750000	3.207026000	-1.489997000
N	-3.994508000	3.623343000	-0.623091000
C	-5.057015000	3.224018000	-1.355931000
C	-6.055159000	4.127766000	-1.047246000
H	-5.015558000	2.358415000	-1.992800000
C	-7.434272000	4.215499000	-1.523030000

C	-8.263416000	5.251503000	-1.077068000
C	-7.953427000	3.277976000	-2.423277000
C	-9.575401000	5.343629000	-1.522352000
H	-7.861387000	5.975586000	-0.380617000
C	-9.265885000	3.373256000	-2.866955000
H	-7.328531000	2.466753000	-2.778645000
C	-10.083517000	4.406964000	-2.418568000
H	-10.205079000	6.151198000	-1.168211000
H	-9.651662000	2.638538000	-3.563647000
H	-11.107330000	4.481097000	-2.764590000
N	-4.301560000	4.712100000	0.110382000
N	-5.538867000	5.017100000	-0.143960000
C	-2.702097000	1.565990000	-0.226549000
C	-2.159084000	0.631737000	-1.098574000
C	-3.298273000	1.109418000	0.955218000
C	-2.192032000	-0.733341000	-0.814926000
H	-1.696081000	0.965969000	-2.020992000
C	-3.344449000	-0.239207000	1.251174000
H	-3.736206000	1.822700000	1.644387000
C	-2.788594000	-1.171383000	0.366080000
H	-1.758153000	-1.433847000	-1.514543000
H	-3.804298000	-0.598446000	2.162826000
O	-2.880537000	-2.475253000	0.743096000
C	-2.325043000	-3.450797000	-0.129774000
H	-2.499403000	-4.410232000	0.350924000
H	-2.819813000	-3.432255000	-1.105467000
H	-1.250188000	-3.294019000	-0.260792000

40

structure of **6**

Fe	-1.459850000	0.110796000	0.079246000
C	-2.973507000	1.451184000	0.060922000
C	-2.828284000	0.887944000	1.367118000
C	-3.249571000	0.381652000	-0.846932000
C	-2.996775000	-0.521953000	1.260944000
H	-2.606665000	1.440754000	2.266576000
C	-3.257295000	-0.834613000	-0.106664000
H	-3.402371000	0.485758000	-1.909684000
H	-2.912281000	-1.232678000	2.067857000
H	-3.404134000	-1.822805000	-0.513217000
C	-0.047619000	-1.185658000	-0.609277000
C	0.215895000	-0.834664000	0.748033000
C	-0.044732000	0.012427000	-1.383337000
H	-0.242650000	-2.179308000	-0.980654000
C	0.381865000	0.580527000	0.813565000
H	0.255161000	-1.516460000	1.582846000
C	0.220797000	1.103846000	-0.503857000
H	-0.235144000	0.083376000	-2.442624000
H	0.570760000	1.156100000	1.705999000
H	0.273358000	2.144736000	-0.783217000
C	-2.880151000	2.901985000	-0.288694000
H	-2.222569000	3.429993000	0.399002000
H	-2.526118000	3.037284000	-1.309003000
N	-4.179179000	3.569820000	-0.208623000
C	-6.129001000	4.164031000	-0.729149000
C	-7.326599000	4.378463000	-1.540229000
C	-8.463371000	4.982270000	-0.991369000
C	-7.357965000	3.980704000	-2.882428000
C	-9.600974000	5.183590000	-1.762762000
H	-8.460055000	5.295085000	0.045895000
C	-8.496171000	4.182886000	-3.650712000
H	-6.480348000	3.513276000	-3.309775000
C	-9.622912000	4.785049000	-3.095890000
H	-10.472299000	5.652254000	-1.321173000

H	-8.504858000	3.869506000	-4.687953000
H	-10.510111000	4.942008000	-3.697270000
N	-4.620304000	4.116346000	0.917432000
N	-5.033316000	3.560245000	-1.219436000
C	-5.857319000	4.506720000	0.608935000
H	-6.468606000	5.009007000	1.339633000

References

45. Telegina, L.N.; Kelbysheva, E.S.; Strelkova, T.V.; Ezernitskaya, M.G., Borisov, Y.A.; Smol'yakov, A.F.; Peregudov, A.S.; Rodionov, A.N.; Ikonnikov, N.S.; Loim, N.M. Transalkylation and Migration of N-Substituent upon Alkylation of 1,2,3-Triazoles Containing Good Leaving N-Substituents. *Eur. J. Org. Chem.***2016**, *2016*, 5897-5906. https://doi.org/10.1002/ejoc.201601146.
46. Telegina, L. N.; Kelbysheva, E. S.; Strelkova, T. V.; Ezernitskaya, M. G.; Smol'yakov, A. F.; Borisov, Y. A.; Lokshin, B. V.; Loim, N. M.Synthesis, molecular structure and photochemical properties of tricarbonyl and dicarbonyl derivatives of 1N- and 2N-cymantrenylalkyl-1,2,3-triazoles. *J. Organomet. Chem.***2018**, *867*, 71-78. https://doi.org/10.1134/S0036023615110200
